# Bispecific DNA‐Peptide Probes for Targeting Receptor Pairs on Live Cells

**DOI:** 10.1002/anie.202514237

**Published:** 2025-08-11

**Authors:** Pritam Ghosh, Huyen Dinh, Alen Kocak, Amal K. Homer, Peter Bou‐Dip, Sophie Schlicht, Oliver Seitz

**Affiliations:** ^1^ Institute of Chemistry Humboldt‐Universität zu Berlin Brook‐Taylor‐Str. 2 D‐12489 Berlin Germany

**Keywords:** Bispecific, Cell targeting, Cyclopeptides, DNA nanotechnology, Multivalency

## Abstract

Chemical modification and nucleic acid self‐assembly can be used to make protein receptor ligands form specific arrangements. While this property has been extensively exploited for probing of homomultivalent interactions, there has been comparatively little attention paid to the exploration of heteromultivalent interactions. In this study, we investigated the use of readily assemblable DNA duplexes for programming bispecific targeting of specific cell types. In contrast to previous bispecific agents, we leverage the potential of peptide‐based high‐affinity binders of cell surface proteins used in diagnostics/therapeutics. Systematic spatial screening revealed the optimal distance between two (cyclo)peptides required for selectively recognizing cells expressing unique combinations of receptors. The VGFR2/α_V_β_3_ receptor system on HUVECs was tolerant to changes of the distance between two cyclopeptides (L and cyclo(‐RGDf(*N*‐Me)K‐)) and required that the distance exceeded the equivalent of 20 nucleotides distance. A different distance‐affinity landscape was observed for recognition of EGFR and MET on A549 cells (through GE11 and bicyclic peptide GE‐137). The DNA‐programmed bispecific binders demonstrated specificity and efficient internalization into target cells. Auristatin‐loaded DNA enabled a selective targeting of cytotoxic payload. Of note, the distance‐optimized bispecific DNA‐peptide probes have much lower molecular weight than previously used agents based on DNA nanostructures or antibodies.

## Graphical abstract

DNA duplex‐programmed presentation of two different (cyclo)peptides provides bispecific agents that recognize unique fingerprints of cells through improved avidity and specificity. Bispecific DNA‐cyclopeptide complexes enabled a cell‐specific targeting and internalization of cytotoxic payload.

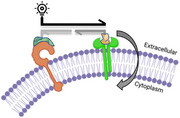

## Introduction

DNA, once viewed as a simple carrier of genetic information, has taken on new life as an advanced material in the rapidly growing field of DNA nanotechnology.^[^ ^1^, ^2^, ^3^, ^4^, ^5^ ^]^ Owing to the highly predictable and specific nature of its base‐pairing interactions, DNA can be uniquely programmed to direct the presentation of a carefully defined number of functional units with known positions and orientations. In a rapidly emerging field, DNA‐nanostructures are used to present ligands to regulate cell behavior or target cancer cells.^[^ ^6^, ^7^, ^8^, ^9^, ^10^, ^11^, ^12^, ^13^ ^]^ Recognition of cell surfaces mostly relies on aptamers. Additionally, cell‐targeted DNA nanostructures have been decorated with proteins and folate receptor ligands to enable targeting and trigger endocytosis. The resulting complexes have a large molecular weight, similar to or even greater than that of antibodies frequently used for tumor targeting.

Bearing in mind the importance of a low molecular weight for efficient penetration of tumor tissue, our aim was to explore cell targeting with DNA complexes much smaller than previously used DNA origami and DNA nanocage structures. We envisioned that by identifying two oncogenic receptors that are uniquely overexpressed in a cancer cell of interest and displaying their ligands on a DNA duplex scaffold, it should be possible to systematically probe the requirements for obtaining enhanced affinity and specificity of cancer cell recognition. The approach is inspired by the bispecific targeting through by‐specific aptamers^[^ ^14^, ^15^ ^]^ and bispecific antibodies,^[^ ^16^, ^17^ ^]^ which, however, mostly are employed for interactions in *trans* to bridge two different cell types.

Instead of aptamer‐ or antibody‐based ligands, we focus here on peptides as smaller ligand alternatives. Notably, there is an invaluable repertoire of peptide‐based high‐affinity binders of cell surface proteins used in diagnostics/therapeutics, which, perhaps surprisingly, has not yet been leveraged for the construction of DNA‐programmed ligand assemblies. Despite the numerous reports emphasizing the importance of optimal distances for probing DNA‐programmed homomultivalent interactions (via display of identical ligands^[^ ^18^, ^19^, ^20^, ^21^, ^22^, ^23^, ^24^ ^]^) with cells^[^ ^25^, ^26^, ^27^, ^28^, ^29^, ^30^ ^]^ and viruses,^[^ ^31^, ^32^, ^33^ ^]^ systematic spatial screening for heterobivalent recognition of cell surface receptors has not been undertaken.^[^ ^34^ ^]^

**Figure 1:**
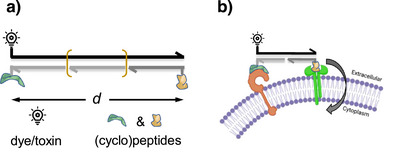
a) DNA‐programmed spatial screening of bispecific probes presenting two different (cyclo)peptides. b) Specificity‐enhanced recognition of cells expressing two different receptors through targeting with bispecific DNA‐peptide probes triggers cell uptake.

Herein we explore the DNA‐programmed heterobivalent display of binders for improved targeting of specific cells (Figure 1a). We show that distance‐optimized bispecific agents provide high specificity for cells expressing two receptors. The approach was tested in two systems: a) cells expressing the vascular endothelial growth factor receptor 2 (VEGFR2) and α_V_β_3_ integrin and b) cells expressing the epidermal growth factor receptor (EGFR) and the MET receptor. The synergistic action of both VEGFR2 and α_V_β_3_ receptors together are key drivers of neovascularization.^[^ ^35^ ^]^ EGFR and MET are commonly found at high levels in lung and breast cancer, making their interaction a focus in the treatment of non‐small cell lung cancer (NSCLC).^[^ ^36^, ^37^ ^]^ To selectively target the “receptor pairs”, we have conjugated cyclopeptide ligands with oligonucleotides. We show that DNA‐programmed spatial screening revealed assemblies with enhanced affinity for cells expressing both receptors, while recognition remained at the monovalent interaction level when cells expressed only one of the receptors. Toxic payload experiments indicate that the bispecific DNA‐peptide assemblies can be internalized (Figure 1b) and confer cell type‐specific cytotoxicity.

## Results and Discussion

**Figure 2:**
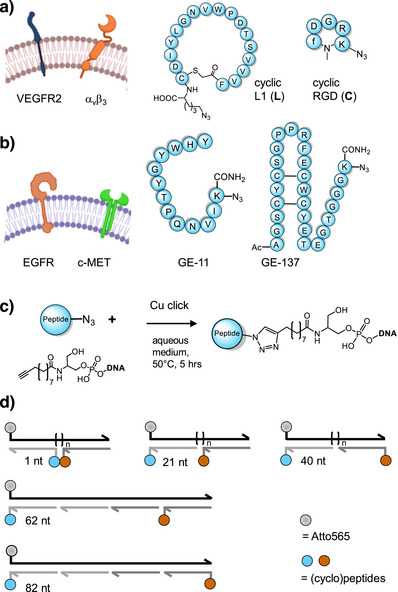
Peptide ligands used for targeting of cells expressing a) VEGFR2 and α_v_β_3_ integrin or b) EGFR and c‐MET. c) Conjugation of azide‐functionalized (cyclo)peptides with alkyne‐modified oligonucleotides. Conditions: 30 mM each of CuSO_4_ and THPTA mixed for 10 min followed by addition of 30 mM ascorbic acid and kept for further 10 min, 4 mM peptide in DMSO, 0.8 mM DNA in H_2_O, pH 6–7, 50 °C, 5 h. d) Configurations of bispecific probes used in this study.

With an aim to explore the potential for increases in binding affinity and specificity, we focused first on a selective targeting of cells overexpressing α_V_β_3_ and VEGFR2 (Figure 2a). For this purpose, two distinct peptide ligands were to be displayed from DNA scaffolds at varying distances. Based on a large body of data, we selected cyclo(‐RGDf(*N*‐Me)K‐) (**C**) as a selective binder of α_V_β_3_ integrins.^[^ ^38^ ^]^ Work from Murakami and coworkers inspired us to select the 17 amino acid long cyclopeptide **L** as binder of VEGFR2.^[^ ^39^ ^]^ The peptides were assembled by solid‐phase peptide synthesis. As previously reported,^[^ ^40^ ^]^ synthesis of cyclo(‐RGDf(*N*‐Me)K‐) involved solid‐phase assembly of Asp(OtBu)‐D‐Phe‐(*N*‐Me)Lys(N_3_)‐Arg(Pbf)‐Gly (α‐*N*‐methylated at Lys(N_3_)) and cyclization in solution, with the azido group at lysine allowing the conjugation with DNA strand (see Supporting Information for further information). For synthesis of **L**, the N‐terminal phenylalanine was capped with a chloroacetyl group, which allowed cyclization through thioether formation after cleavage from the synthesis resin. A different set of peptides was used to explore the heterobivalent presentation of ligands to the EGFR and the MET receptor (Figure 2b). Based on ample evidence for successful targeting of EGFR, the 12‐amino acid‐long peptide GE‐11^[^ ^41^, ^42^ ^]^ was selected and equipped with an additional azidolysine residue to enable conjugation with oligonucleotides. For recognition of MET, we settled on the 26 amino acid peptide GE‐137 introduced by Burggraaf et al. as probe for detection of polyps in colon.^[^ ^43^ ^]^ While the synthesis of GE‐11 was straightforward, preparation of GE‐137, which contains two disulfide bridges, in a form enabling conjugation with oligonucleotides proved more challenging. Adapting a method introduced by Brik, the disulfide bridges were installed in two consecutive steps.^[^ ^44^ ^]^ The first disulfide linkage was formed using disulfiram. After purification, treatment with PdCl_2_ removed acetamidomethyl protecting groups from the two remaining cysteine residues and established the second disulfide bridge.

Next, the azido‐modified peptides were conjugated with 20 or 21 nucleotide‐long DNA strands (Figure 2c) by Cu‐Click reactions. Nucleic acid hybridization with 41 or 83 nucleotide‐long, fluorescence‐labeled template strands and shorter filler strands was used to assemble complexes, which can display the two peptide ligands at distances of 1, 21, 40, 62, or 82 nucleotides (Figure 2d). These nicked duplex architectures are less rigid than the DNA nanostructures that have previously been used for interactions with cells. However, by using similar DNA scaffolds in homobivalent interactions, we have previously demonstrated that large binding enhancements can be obtained.^18^, ^19^, ^21^, ^25^, ^32^, ^34^ The integrity of the formed complexes was confirmed by native PAGE for two representative constructs (Figure S35).

**Figure 3:**
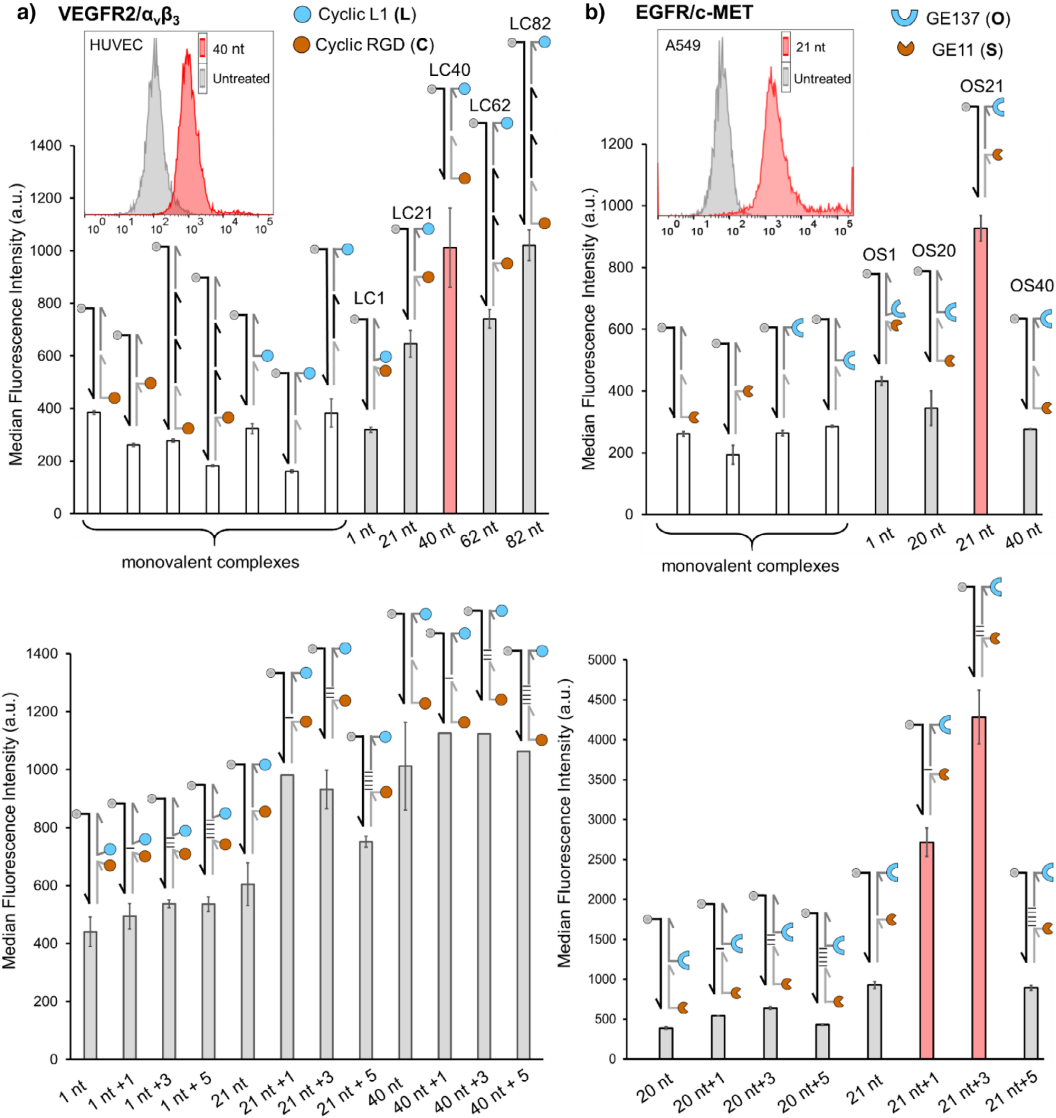
Staining of a) HUVECs or b) A549 cells with monovalent and heterobivalent DNA‐peptide complexes assessed by flow cytometry of live cells. Conditions: 200 nM probe for HUVEC cells and 750 nM for A549 cells, 10 min incubation in PBS at 37 °C, 5% CO_2_.

Human umbilical vein endothelial cells (HUVEC) express VEGFR2 and α_v_β_3_ integrin and are an accepted model for endothelial dysfunction and tumor‐associated angiogenesis. Live HUVECs were treated with complexes presenting both cyclopeptides **L** and **C**, or only one of the ligands (Figure 3a, upper). The staining intensity was determined by flow cytometry.

Increasing the distance between the two cyclopeptides resulted in increased staining intensity. Complexes presenting both ligands at 40 (LC40) and 82 (LC82) nucleotides distance stained HUVECs with much higher intensity than monovalent complexes. Concentration‐dependent measurements showed that heterobivalent complex LC40 had higher affinity than monovalent complexes (Figure S43). Target engagement was demonstrated in siRNA knockdowns (Figure S39).

A markedly different distance–affinity relationship was observed with A549 cells, which express both the EGFR and the MET receptor. In this case (Figure 3b, upper), staining intensity peaked when the respective ligands—the GE11 and GE‐137 peptides—were displayed at a 21‐nucleotide distance (OS21), which presents the ligands at a distance of approx. 66 Å, assuming structures of a B‐type DNA duplex. At larger or shorter distances, the affinity resembled that of monovalent complexes.

Next, unpaired nucleotides were introduced between the duplex segments of the nicked duplexes. The addition of 1, 3, or 5 unpaired spacer nucleotides in LC1 complexes presenting **L** and **C** had little impact on binding to HUVECs (Figure 3a, lower), suggesting that the gain in scaffold flexibility has no effect when the distance between the two ligands is too low to allow simultaneous recognition of the two receptors. However, greatly improved binding was observed when a single spacer nucleotide was inserted into complexes of the LC21 type. At ligand–ligand distances in the range of 41 paired nucleotides unpaired spacer nucleotides had, again, little effect. Dramatic improvements were observed with unpaired spacer nucleotides in complexes presenting EGFR and c‐MET ligands for recognition of A549 cells (Figure 3b, lower). A single nucleotide inserted between the duplex segments in the 21 nt + 1 complex increased the staining intensity by a threefold. Further improvements were obtained with an insertion of three unpaired spacer nucleotides. Remarkably, a spacer consisting of five unpaired nucleotides resulted in a marked decrease in binding affinity. The extended single‐strand region provides the DNA scaffold with high flexibility. It is conceivable that this leads to an entropic disadvantage of heterobivalent recognition. As an important result of this comparative study, we conclude that each of the 2‐receptor systems (i.e., VEGFR2/α_v_β_3_ on HUVECs, EGFR/c‐MET on A549 cells) has a characteristic distance–affinity profile.

In subsequent studies, we focused on the VEGFR2/α_v_β_3_ receptor pair. Exploring the potential for further affinity improvements, multiple copies of ligands were introduced. A large body of work has shown that the α_v_β_3_ integrin is amenable to cluster effect‐induced binding enhancements. Indeed, the staining intensity was found positively correlated with the number of C ligands units displayed from the DNA scaffold (Figure S40).

**Figure 4:**
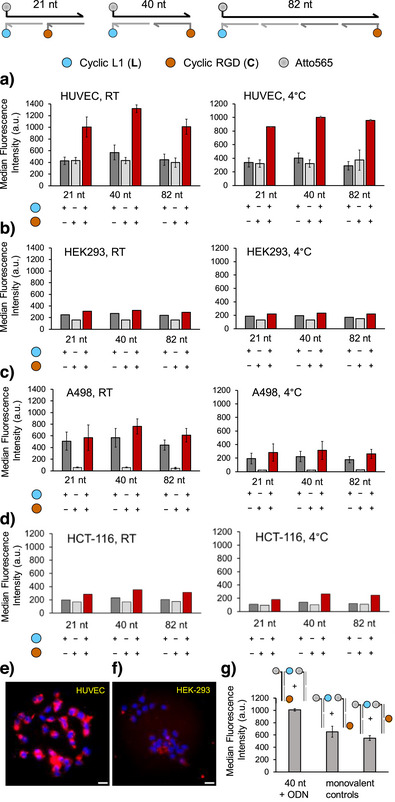
Specificity of staining a) HUVECs (VEGFR2+, α_v_β_3_+), b) HEK293 cells (VEGFR2−, α_v_β_3_−), c) A498 cells (VEGFR2−, α_v_β_3_+), and d) HCT‐116 cells (VEGFR2+, α_v_β_3_−) with heterobivalent and monovalent DNA‐peptide complexes assessed by flow cytometry. Fluorescence micrographs of e) HUVECs and f) HEK293 cells after treatment with 500 nM LC40. g) Comparison of staining intensity after treatment of HUVECs with a mixture of 250 nM LC40 and 250 nM “empty” duplex or mixtures of two monovalent complexes at 250 nM each. Conditions: a–d) 200 nM or e), f) 500 nM probe in HBSS. BB (1× HBSS, 1% BSA, 0.5 mg mL^−1^ DNA) for HUVEC cells and DMEM with (10% FBS and 1% penicillin/streptomycin) for HEK‐293, incubated for 1 h, 4 °C, 5% CO_2_. 50 000 cells seeded per well of HUVEC and HEK cells, blue: nuclear labeling Hoechst33342, *λ*_ex_  =  350 ± 50 nm, *λ*_em_ = 460 ± 50 nm; red: Atto‐565 in TRITC channel, *λ*_ex_ = 575 ± 25 nm, *λ*_em_ > 593 nm. Scale bar is 20 µm.

Next, we assessed the cell‐type specificity in experiments including HUVECs (VEGFR2+, α_v_β_3_+), HEK293 cells (VEGFR2−, α_v_β_3_−), A498 cells (VEGFR2−, α_v_β_3_+), and HCT‐116 cells (VEGFR2+, α_v_β_3_−) (Figure 4). LC40 and LC82 were included in this study due to the high HUVEC staining intensity (see Figure 3a). This comparison also served the purpose of exploring the potential for DNA length‐dependent unspecific binding, which is why LC21 was also included due to its small size and its increased affinity in comparison to monovalent binders (see Figure S43). Flow cytometry measurements showed that the intensity of HUVEC staining with heterobivalent complexes was higher than staining obtained by monovalent complexes of the same size (Figure 4a). This property was observed for incubation at both 25 °C and 4 °C indicating that receptor‐mediated internalization has little effect on signal intensity (Figure 4a, left versus right). Of note, staining with heterobivalent complexes remained in the range of monovalent complexes when one or both receptors were lacking in A498, HEK293, and HCT‐116 cells (Figure 4b–d). Fluorescence micrographs showed that LC40 was readily internalized by HUVECs (Figures 4e and S41). As expected from flow cytometry data, fluorescence micrographs of HEK293 cells showed very little contrast (Figure 4f). Interestingly, the uptake of LC40 into HUVECs was more efficient than uptake of LC82 (Figure S41) suggesting that the size and/or the negative charge of the internalized cargo does matter. To shine light on the synergism provided by the potential to bridge VEGFR2 and α_v_β_3_, HUVECs were incubated with a mixture of both monovalent complexes or a mixture comprised of heterobivalent LC40 and an “empty” complex (Figure 4g). Although the concentration of fluorescence dye and cyclopeptide ligands was identical in both experiments, LC40 clearly provided higher staining intensity suggesting that in addition to an increase in specificity heterobivalent ligand presentation can also enhance avidity.

**Figure 5:**
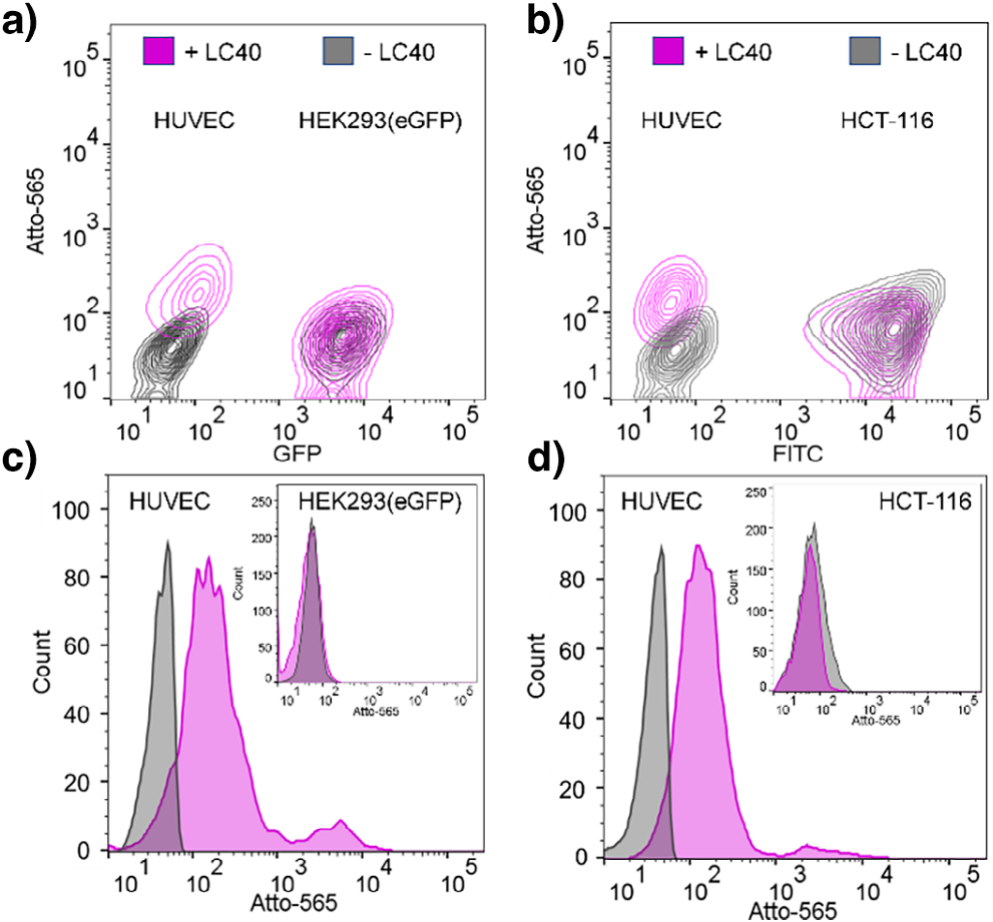
Specificity of HUVEC staining with Atto565‐labeled LC40 in mixtures including a) GFP‐expressing HEK293 cells (VEGFR2−, α_v_β_3_−) or b) anti‐EpCAM antibody‐stained HCT‐116 cells (VEGFR2+, α_v_β_3_−). Upper images show contour plots, lower images show histogram analyses. Conditions: 100 000 cells of HUVECs and 100 000 cells of HEK293(eGFP) or 100 000 cells of HCT‐116 cells seeded per well, incubated for 10 min, 37 °C, 5% CO_2_ in PBS. Staining: HCT‐116 cells were treated initially with anti‐EpCAM antibody, 200 nM LC40, and 200 nM ligand‐ and label‐free LC40, if added. GFP/FITC, excitation: 488 nm, BP 527/32 and LP 507/5; Atto‐565, excitation: 561 nm, BP 613/18 and 605/10. BP: Bandpass filters and LP: longpass filters.

Specificity was further examined in experiments involving mixed cell populations. HUVECs were mixed with HEK‐293(eGFP) cells that had been stably transfected with a construct leading to expression of an eGFP‐MS2BP fusion protein (see 3.2.8, Supporting Information). Flow cytometry analysis of the mixed cell sample showed that LC40 stained the HUVEC cell population, leaving the HEK cells unaffected (Figure 5a). Given the weak yet noticeable staining of HCT‐116 cells with LC40 (see Figure 4d), it was of interest to also include this cell line in mixed cell experiments. HCT‐116 cells were distinguished from HUVECs using an anti‐EpCAM antibody. Treatment with LC40, again, shifted HUVECs to higher Atto565 fluorescence intensities, whereas the Atto‐565 signal provided by EpCAM‐stained HCT‐116 cells remained low (Figure 5b).

**Figure 6:**
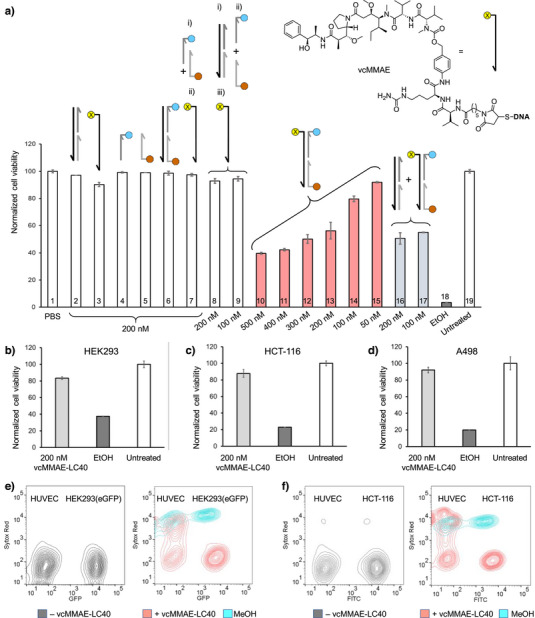
Cytotoxicity of cathepsin B‐cleavable vcMMAE‐DNA‐peptide complexes LC40 and components. a) Viability of HUVECs after treatment with unlabeled DNA complex 2), vcMMAE‐template DNA 3), single‐stranded monovalent DNA‐peptide conjugates 4 and 5), heterobivalent LC40 lacking cytotoxic payload 6), both i) single‐stranded monovalent DNA‐peptide conjugates followed by ii) addition of vcMMAE‐template 7), with i) unlabeled DNA complex followed by addition of ii) both single‐stranded monovalent DNA‐peptide conjugates and subsequently iii) vcMMAE‐template 8 and 9), vcMMAE‐LC40 at varied concentrations 10–15) or with unlabeled DNA complex prior to addition of vcMMAE‐LC40 16 and 17). Viability of b) HEK‐293 cells (VEGFR2−, α_v_β_3_−), c) HCT‐116 cells (VEGFR2+, α_v_β_3_−), and d) A498 cells (VEGFR2−, α_v_β_3_+). Conditions: 10^4^ cells well^−1^ in 100 µL medium, propagated 24 h to 85% confluency, 10 min incubation in PBS, 37 °C, 5% CO_2._ Columns 8–9 and 16–17: 20 min incubation over all steps. Column 1, 20 min incubation. All: 6 h incubation with 10% Alamar blue in full medium. Flow cytometric analysis of HUVECs mixed with e) eGFP‐expressing HEK‐293 cells or f) anti‐EpCAM antibody‐stained HCT‐116 cells assessed by dead cell staining. Left: untreated cell; right: cells treated with 200 nM vcMMAE‐LC40 or MeOH. Conditions: 100 000 cells each mixed in a sterilized Eppendorf, incubated for 10 min, 37 °C, 5% CO_2_ in PBS, followed by 4 h incubation in full medium at 37 °C, then 10 min incubation with 5 nM Sytox Red in PBS. GFP/FITC, excitation: 488 nm, BP 527/32 and LP 507/5; Sytox Red, excitation: 640 nm, BP 660/10. BP: bandpass filters and LP: longpass filters.

To inhibit proliferation of select cells, targeting molecules are frequently conjugated with cytotoxic payloads. Oligonucleotide hybridization offers a convenient means to equip the DNA‐programmed ligand assemblies with such cargo. Commonly used cytotoxic payloads such as the tubulin polymerization inhibitor monomethyl auristatin E (MMAE) exert their effects intracellularly and, therefore, require internalization of conjugates into cells. Furthermore, payload, in conjugated or cleaved form, must be able to escape endocytotic vesicles. Both receptor‐mediated endocytosis and endosomal escape could be problematic when cytotoxic agents are linked to large DNA assemblies. However, fluorescence microscopy of HUVECs incubated with fluorescence‐labeled LC40 indicated successful internalization (Figures 4e and S41). A thiolated template strand was equipped with vcMMAE via maleimide conjugation (Figure 6). The resulting thiol‐maleimide‐linked vcMMAE‐DNA conjugate was annealed with shorter strands to prepare the bispecific agent vcMMAE‐LC40. A lysosomal protease‐cleavable dipeptide, valine–citrulline, was inserted between the DNA and the MMAE to facilitate release of MMAE upon cleavage by cathepsin B.

HUVECs were incubated with the oligonucleotide assemblies for 10 min only. Cell viability was assessed 6 h later by means of an AlamarBlue assay (Figure 6a). After treatment with 200 nM vcMMAE‐LC40, only 56% of the cells remained viable (column 13). Combined annexin V and propidium iodide staining showed that a total of 77% of treated HUVECs were undergoing apoptosis or necrosis (Figure S45). For comparison, HUVECs were treated with a nonlabeled version of LC40, which lacked the MMAE payload (column 6). Cell viability remained high indicating that the cytotoxic activity is not due to simultaneous targeting of VEGFR2 and α_v_β_3_ per se. Likewise, incubation with cyclopeptide‐loaded single strands (columns 4 and 5) or a ligand‐free DNA complex (column 2) did not affect cell viability. A clear dose‐response was obtained upon escalation of the vcMMAE‐LC40 concentration (columns 10–15), which conforms with the increases in fluorescence intensity observed upon dose escalation of the fluorescence‐labeled LC40 (Figure S43). Of note, pre‐treatment of cells with a nonlabeled DNA complex was found to improve the cytotoxic activity of vcMMAE‐LC40 (compare column 17 with 14, Figure 6a). We speculate that this is due to the presence of scavenger receptors or unspecific cell surface binding, which is saturated upon treatment with the nonlabeled DNA, thereby increasing the concentration of vcMMAE‐LC40 available for targeting VEGFR2 and α_v_β_3_.

Next, we explored whether assemblies could be formed in situ on the cell surface. In this scenario, the small cyclopeptide–oligonucleotide conjugates would first bind their respective cell surface receptors. The bispecific assembly would then be formed upon addition of the template strand to the cell surface‐bound oligonucleotides. Such approaches might facilitate targeting of tumor tissue when the individual components penetrate tissue more effectively than the full‐size assembly. However, the decrease in cell viability was rather low when HUVECs were treated with the ligand–oligonucleotide conjugates prior to the addition of the vcMMAE‐template strand (columns 7–9). The minor—if not negligible—viability decrease was also observed in experiments, which involved incubations with the MMAE‐template strand in absence of ligand conjugates (column 3). This suggests that targeting of VEGFR2 and α_v_β_3_ with L‐ and C‐cyclopeptide conjugates may not be amenable to in situ assembly, probably because ligand‐induced receptor internalization is occurring rapidly prior to addition of the template strand.

Subsequently, we assessed the cytotoxic activity of vcMMAE‐LC40 on different cell lines expressing VEGFR2 and α_v_β_3_ in combination (HUVEC (VEGFR2+, α_v_β_3_+)), mutually exclusively (A498 (VEGFR2−, α_v_β_3_+)), HCT‐116 cells (VEGFR2+, α_v_β_3_−)), or not at all (HEK (VEGFR2−, α_v_β_3_−)). While only half of the HUVECs survived the treatment with 200 nM vcMMAE‐LC40 (Figure 6a, column 13), effects on A498 cells, HCT‐116 cells, and HEK‐293 cells were modest with ≥83% of cells remaining viable (Figure 6b–d). The HUVEC‐specific cytotoxicity was further verified in mixed cell experiments involving mixtures of HUVECs with either HEK293(eGFP) or HCT‐116 cells. While the HEK cells remained intact, 55% of the HUVECs were found to be dead (see Figures 6e and S48A). Similarly, 67% of HUVECs died upon treatment with vcMMAE‐LC40 in HUVEC/HCT‐116 mixtures, whereas HCT‐116 cells proved unresponsive (Figures 6f and S48B). The majority (≥70%) of HUVECs were undergoing apoptosis after a short treatment with vcMMAE‐LC40 (Figures S46 and S47). For comparison, the cell lines were incubated with 200 nM MMAE in free form (Figure S44). Due to its cell permeability, MMAE can exert cytotoxic effects. But in this case, HEK‐293 cells, which lack VEGFR2 and α_v_β_3_, proved most vulnerable. The different cytotoxicity profiles clearly indicate that cell type specificity is encoded by bispecific presentation of cyclopeptides on vcMMAE‐LC40.

## Conclusion

In summary, we demonstrated that DNA duplexes can enhance the targeting of cells that express specific combinations of surface receptors by displaying peptides and cyclopeptides in a heterobivalent manner. Compared to the DNA nanostructure‐aptamer and ‐protein complexes used previously,^[^ ^6^, ^7^, ^8^, ^9^, ^10^, ^11^, ^12^, ^13^ ^]^ the assemblies employed in this study have a much lower molecular weight. This could help to reduce costs and facilitate tissue penetration. Following the conjugation of (cyclo)peptides with oligonucleotides, bispecific agents were assembled by means of nucleic acid hybridization. Displaying an *N*‐methylated cyclic RGD peptide (cyclo(‐RGD(*N*‐Me)K‐)) for binding of α_v_β_3_ integrin and a thioether‐cyclized 17 amino acid peptide (L) for targeting of VEGFR2 enabled the enhanced recognition of human umbilical vein endothelial cells (HUVEC). The VEGFR2/α_v_β_3_ pair demonstrated tolerance to distance changes, provided that the distance exceeded the equivalent of 20 nucleotides of a DNA duplex. Of note, the affinity gain persisted until 270 Å and despite the incorporation of (5 nt long) single‐stranded segments. A key finding of this study is the distinct distance‐affinity profile that was observed when DNA scaffolds presented (cyclo)peptides specific for the epidermal growth factor receptor (EGFR) and the hepatocyte growth factor receptor (MET), which are expressed on A549 cells. In this instance, the affinity was observed to reach a maximum at an approximate distance of 70 Å, with a decline in affinity noted for displays presenting the two peptides at distances that were either larger or smaller than this. The measurements of four distinctive cell lines, which differed in their VEGFR2/α_v_β_3_ expression patterns, indicated that the bispecific binders exhibited a high degree of specificity in recognizing cells. The DNA‐programmed bispecific targeting of VEGFR2 and α_v_β_3_ allowed the efficient internalization of the DNA assemblies. Cell viability measurements combined with flow cytometry analysis of mixed cell populations demonstrate that auristatin‐loaded DNA complexes enabled a selective targeting of cytotoxic payload to a specific cell type.

DNA duplexes are vulnerable to degradation by nucleases. Therefore, for applications in animal studies, it would be necessary to enhance their stability through nucleic acid modifications known from antisense and related technologies. Our data shows that the nucleic acid toolbox offers promising avenues for enhanced targeting of specific cell types through the action of heteromultivalent peptide and cyclopeptide displays.

## Conflict of Interests

The authors declare no conflict of interest.

## Supporting information



Supporting Information

## Data Availability

The data that support the findings of this study are available in the Supporting Information of this article.
