# Supplementary material for: Bispecific DNA‐Peptide Probes for Targeting Receptor Pairs on Live Cells
Source: Angew Chem Int Ed Engl. 2025 Aug 11;64(39):e202514237. doi: 10.1002/anie.202514237 (PMC12455437; doi:10.1002/anie.202514237)
Supplement: Supplementary file 1 — Supporting Information [file ANIE-64-e202514237-s001.pdf]

Bispecific DNA-Peptide Probes for Targeting Receptor Pairs on Live Cells

Pritam Ghosh,[a] Huyen Dinh,[a] Alen Kocak,[a] Amal K Homer,[a] Peter Bou-Dip,[a] Sophie Schlicht,[a] Oliver Seitz*[a]

[a] Dr. Pritam Ghosh, Dr. Huyen T. Dinh, Alen Kocak, Amal K Homer, Peter Bou-Dip, Sophie Schlicht, Prof. Dr. Oliver Seitz

Institute of Chemistry

Humboldt-Universität zu Berlin

Brook-Taylor-Str. 2, D-12489 Berlin, Germany

E-mail: oliver.seitz@chemie.hu-berlin.de

Contents

1. Instrumentation **3**

2. Experimental Procedures Chemistry **3**

3. Cell Culture and Experiments with Cells **17**

4. UPLC Traces and Mass Spectra **32**

5. Gating Strategy in Flow cytometry **41**

6. Binding of DNA-free peptides to HUVEC and A549 cells **42**

7. Native PAGE for dsDNA **43**

8. Analyzing Cell Surface Receptors Using Antibodies against
VEGFR2 and $\alpha v\beta_3$ Integrin **44**

9. siRNA knock down **46**

10. Multiple cRGD on the DNA Scaffold: Multivalency **47**

11. Fluorescence Microscopy **48**

12. Concentration dependent flow cytometry **50**

13. Cytotoxicity of non-conjugated MMAE **50**

14. Characterization of Cell Death **51**

15. Flow Cytometry Analysis of Mixed Cell Populations **52**

1. Instrumentation

Automated SPPS was performed using a MultiPep RS peptide synthesizer (Intavis, Cologne, Germany) at a 25 µmol scale using fritted columns (10 mL) purchased from Carl Roth (Karlsruhe, Germany). RP-HPLC purifications were done using an Agilent Technologies 1100 Series HPLC system (Santa Clara, USA) with a Polaris C18 column (5µm, 250 x 10, pore size: 220 Å) from Varian at a flow rate of 6 ml/min. A multiple wavelength detector operating at three wavelengths (λ_1 = 210 nm, λ_2 = 260 nm, and λ_3 = 280 nm) was used with a binary mixture of mobile phase A (98.9 % H_2O, 1 % ACN, 0.1 % TFA) and B (98.9 % ACN, 1 % H_2O, 0.1 % TFA) in a linear gradient as described.

Ultra-high-performance liquid chromatography (UPLC) was performed for analytical data with an Acquity UPLC on an Acquity H-Class system (Waters, Milford, MA, USA) equipped with a PDA (λ = 210 nm) and QDa detector, using an Acquity UPLC CSH C18 (2.1 x 50 mm; 1.7 µm; 130Å) column. Analyses were performed using the same solvent system A/B as in the semi-preparative HPLC, and samples were eluted at 50°C. Mass analysis was conducted with a Waters XEVO G2-XS QTof analyzer, and the recorded data was subsequently analyzed using the provided built-in software. Agilent 1260 infinity II and infinity lab LC/MSD were also used for UPLC.

Semi preparative HPLC for DNA oligomers was carried out on a Gilson 1105 HPLC System (Gilson, Limburg, Germany). The mobile phase consisted of a binary mixture of A (0.1 M triethylammonium acetate buffer, pH = 7.4, aq.) and B (acetonitrile). For DNA-peptide conjugates, analysis was performed using Acquity UPLC Oligonucleotide BEH C18 Columns, (50/2.1 mm; 1.7µM; 130Å) at 50°C in both the cases and binary mixture of A (0.1M triethylammonium acetate pH: 7.5) and B (acetonitrile) was used.

To determine the peptide and DNA-peptide conjugate concentration, the optical density of the corresponding solutions was measured using a NanoDrop spectrophotometer (PeqLab, Erlangen, Germany) against the medium blank.

2. Experimental Procedures Chemistry

2.1 Reagents for Chemical Synthesis

Unless otherwise stated, all solvents and reagents were used as received from commercial suppliers. Fmoc-protected amino acids (AAs) building blocks [Fmoc-Gly-OH, Fmoc-Ala-OH, Fmoc-Val-OH, Fmoc-Leu-OH, Fmoc-Ile-OH, Fmoc-Phe-OH, Fmoc-His(Trt)-OH, Fmoc-

Asn(Trt)-OH, Fmoc-Gln(Trt)-OH, Fmoc-Arg(Pbf)-OH, Fmoc-Lys(Boc)-OH, Fmoc-Lys(N_3)-OH, Fmoc-Tyr(tBu)-OH, Fmoc-Ser(tBu)-OH, Fmoc-Thr(tBu)-OH, Fmoc-Asp(OtBu)OH, Fmoc-Glu(OtBu)-OH, Fmoc-Cys(trt)-OH, Fmoc-Cys(ACM)-OH, Fmoc-D-Phe-OH, Fmoc-Pro-OH, Fmoc-Trp(boc)-OH], O-(7-Azabenzotriazol-1-yl)-N,N,N′,N′-tetramethyluronium hexafluorophosphate (HATU), were purchased from Carbolution Chemicals (St. Ingbert, Germany). TentaGel® S TRT Cl and TentaGel® R RAM resins were obtained from Rapp Polymere GmbH (Tübingen, Germany) with a 0.22 µmol/mg and 0.19 µmol/mg loading, respectively. Ethyl (2Z)-2-cyano-2-(hydroxyimino)acetate (Oxyma) and O-(1H-6-Chlorobenzotriazole-1-yl)-1,1,3,3-tetramethyluronium hexafluorophosphate (HCTU) were purchased from Carbolution Chemicals GmbH (Saarbrücken, Germany). N,N-Diisopropylethylamine (DIPEA), N-Methylpyrrolidone (NMP) and Trifluoroacetic acid (TFA) were obtained from Carl Roth GmbH (Karlsruhe, Germany). 1H-1,2,3-Benzotriazol-1-ol (HOBt) was from abcr GmbH (Karlsruhe, Germany). N,N-Dimethylformamide (DMF) and acetonitrile (ACN) were purchased from VWR International GmbH (Darmstadt, Germany). Thionyl chloride, Chloro acetic acid (Cl-acetic acid), o-NBS-Cl (2-Nitrobenzenesulfonyl chloride), DBU (1,8-Diazabicyclo[5.4.0]undec-7-ene) and 2-ME (mercapto ethanol) were purchased from Sigma Aldrich. AF-568 was purchased from lumiprobe. 5(6)-Carboxytetramethylrhodamine was purchased from Sigma aldrich. THPTA ligand for copper click reaction was obtained from BLD pharma. $CuSO_4.5H_2O$ was purchased from Merck. Ascorbic acid was purchased from Carl Roth. TAMRA alkyne, 5-isomer was purchased from Lumiprobe. Maleimide functionalized vcMMAE was purchased from BLD pharma. Template strand gcgtaaggagatctggaatgagtcgagctccataataagcg with 5':Atto 565 and thiol, gcgtaaggagatctggaatgaatccagtcgagctccataataagcg (+5 spacer nucleotide) with 5':Atto 565, gcgtaaggagatctggaatgaatcgtcgagctccataataagcg (+3 spacer nucleotide)with 5':Atto 565, gcgtaaggagatctggaatgatgtcgagctccataataagcg (+1 spacer nucleotide) with 5':Atto 565, strand 1 5'-cgc tta tta tgg agc tcg ac-3' with alkyne modification on either position, strand 2 5'-tca ttc cag atc tcc tta cgc -3' with alkyne modification on either position are purchased from Biomers, Germany and used without further purification.

2.2 Automated solid phase peptide synthesis (SPPS)

Swell: The resin is allowed to swell for 10 minutes in DMF (800 µL).

Fmoc removal treatment of the resin with 20 % piperidine in DMF (800 µl) for 2x 5 min). Subsequently, the resin was washed with DMF (4x, 1000 µl).

Coupling: Fmoc-protected amino acids (8.25 eq.), dissolved in a solution of NMP containing OxymaPure (8 eq.) were activated with HCTU (8 eq.) and NMM (24 eq.) This solution (0.6. M in Fmoc-protected amino acid) was transferred to the resin [(double coupling for the first amino acid with 45 minutes each time, then single coupling for otheramino acids (coupling time 60 min)]. Subsequently, the resin was washed with DMF (3x, 1000 µl).
Capping: the resin was treated with DMF:Ac_2O:lutidine (89:5:6 v/v/v, 800 µl) for 10 min. Subsequently, the resin was washed with DMF (3x, 1000 µl).

2.3 Synthesis of Cyclic L1

To prepare cyclic L1 peptide (Fig. S1), a fritted syringe reactor was charged with the TentaGel® S TRT Cl resin required for 25 µmol scale. The resin was allowed to swell in anhydrous dichloromethane (DCM, 2x, 1 mL) for 30 min two times, followed by activation with 5% $SOCl_2$ (v/v) in dry DCM (2x, 1 mL) for 2 x 20 min at ambient temperature while shaking. The resin was washed five times with dry DCM (1 mL each time for 1 min) afterwards and twice with DMF (1mL each time for 1 min). Subsequently, the resin was functionalized using four eq. Fmoc-Lys (N_3)-OH with 8 eq. DIPEA in DMF (concentration: 0.2 M) for 2 x 1 hr at room temperature with shaking.The resin was washed thoroughly with DMF (5x, 1mL) and attached to the synthesizer for the following couplings via SPPS (as shown in section 2.2). After completion of the peptide assembly, removal of the Fmoc-group was performed by treatment of the resin with the deprotection solution (20% piperidine in DMF, 800 µl, 2x5 min). Subsequently, the resin was washed with DMF (4x, 1000 µl). Chloroacetic acid was coupled upon treatment of the resin with 4 eq. chloroacetic acid, 3.85 eq. HATU and 8 eq. DIPEA in DMF (concentration: 0.2M) for 2 hrs at room temperature, followed by washing with DMF (3x, 1 mL), then DCM (5x, 1 mL) and dried under a vacuum. The global deprotection was performed with the cleavage cocktail (2 ml) TFA/triisopropylsilane/H_2O 95:2.5:2.5 (v/v/v) at room temp. After 2 h the cleavage cocktail was collected by filtration in 15 mL falcon, the resin was washed with the cleavage cocktail (3x 1 mL) and the combined filtrates were concentrated (~ 0.5 mL) under compressed airflow. Et_2O (10-fold volume) was added to the remaining residue, the suspension was cooled (in dry ice for ~30 min) and centrifuged (4000 rpm, 15 min, 4ºC). Afterwards, the ether phase was decanted. The remaining peptide pellet was dissolved in 1 mL of ACN: water (1:1, v/v with 0.1% TFA) by vortexing. The solution in the falcon was frozen by liquid nitrogen in a Dewar flask and was lyophilized. The lyophilized powder was solubilized in DMSO (100 µM) followed by a dropwise addition (10µL at-a-time) of

water:triethylamine mixture (5:1, v/v, pH=8, final concentration: 50μM). After 6 hrs. the solvent was removed in rotary evaporator and dissolved in 1 mL of ACN: water (1:1, v/v with 0.1% TFA) by vortexing, followed by lyophilization and purification using RP-HPLC. The sequence is shown in Fig. S1. The UPLC trace, along with the mass spectrum, are shown in Fig. S13 and retention time, UPLC gradient, yield is tabulated in table S1.

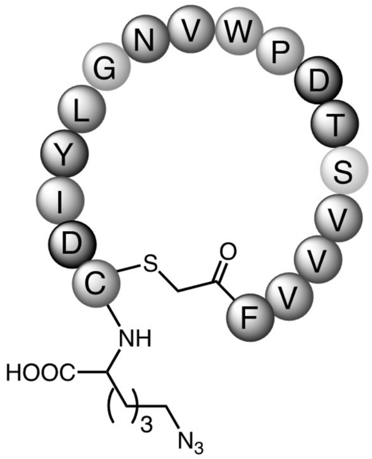

Fig. S1 Amino acid sequences in cyclic L1.

2.4 Synthesis of Cyclic RGD

To prepare cyclic RGD peptide (Fig. S2), a fritted syringe reactor was charged with the TentaGel® S TRT Cl resin required for a 25 μmol scale. The resin was allowed to swell in anhydrous dichloromethane (DCM, 2x, 1 mL) for 30 min two times, followed by activation with 5% $SOCl_2$ (v/v) in dry DCM (2x, 1 mL) for 2 x 20 min at ambient temperature while shaking. The resin was washed five times with dry DCM (1 mL each time for 1 min) afterwards and twice with DMF (1 mL each time for 1min). Subsequently, the resin was functionalized using four eq. Fmoc-Gly-OH with 8 eq. DIPEA in DMF (concentration: 0.2M) for 2 x 1 hr at room temperature with shaking.

a. Peptide Synthesis: Fmoc-Gly-loaded resin was treated with the deprotection solution (20% piperidine in DMF, 800 μl, 2x5 min). Subsequently, the resin was washed with DMF (4x, 1000 μl). After Fmoc cleavage, Fmoc-L-Arg(Pbf)-OH and Fmoc-L-Lys(N_3)-OH were coupled in double couplings by using 4 eq. respective AAs, 3.85 eq. HATU and 8 eq. DIPEA in DMF (concentration: 0.2M). Each coupling was performed at room temperature for 1 hr.

b. N-Methylation: After Fmoc cleavage (treatment with 20% piperidine in DMF (800 μl), 2x 5 min, followed by washes with DMF (4x, 1000 μl)), the resin was treated with 4 eq. of o-NBS-Cl (2-nitrobenzenesulfonyl chloride) and 10 eq. of lutidine in 2 mL of NMP for 30 minutes at

room temperature. The resin was washed with NMP two times. The procedure was repeated once. The resin was then treated with 3eq. DBU (1,8-Diazabicyclo[5.4.0]undec-7-ene) in 1 mL NMP for 30 min with shaking, followed by adding 10 eq. DMS in 1 mL NMP (total 2 mL NMP with the resin). After 30 min the resin was washed with NMP (6x, 1mL). The procedure was repeated once.

c. o-NBS Deprotection: The resin was treated with 10 eq. of 2-mercapto ethanol and 5 eq. of DBU (1,8-Diazabicyclo[5.4.0]undec-7-ene) in 2 mL of NMP for 30 minutes at room temperature. After washing with NMP (3x, 1 mL), the procedure was repeated once.

d. Amino Acid Coupling on N-Methylated Peptide:

(i) coupling of Fmoc-D-Phe-OH: The resin was allowed to react for two hours at room temperature with a mixture of 4 eq. of Fmoc-D-Phe-OH, 10 eq. of DIC and 10 eq. of Oxyma in NMP (concentration: 0.2M). After washing with NMP (3x, 1mL), the procedure was repeated once. Subsequently, the resin was washed thoroughly with NMP two times (1 mL each), followed by DMF three times (1mL each).

(ii) coupling of Fmoc-L-Asp(tBu)-OH: After Fmoc cleavage (20% piperidine in DMF, 800 µl, 2x 5 min) and washing with DMF (3x, 1 mL), the resin was treated with a mixture of 4 eq. of Fmoc-L-Asp(tBu)-OH, 3.85 eq. HATU, 4 eq. of Oxyma and 8 eq. DIPEA in DMF (concentration: 0.2M). After 2 hrs, the resin was washed thoroughly with DMF three times (1 mL each). The Fmoc group was removed using 20% piperidine in DMF, 800 µl, 2x 5 min, followed by washing with DMF (3x, 1 mL) and DCM (5x, 1 mL).

e. Cleavage of the Peptide: The resin was treated with 20% TFE in dry DCM for 1 hr. The cleaved mixture was collected. The resin was washed two times with 1 mL of 20% TFE in dry DCM for 1 minute each, the solution was collected and evaporated with rotary evaporator. The oily residue was solubilized with 1 mL of ACN: water (1:1, v/v with 0.1% TFA) by vortexing. The solution in the falcon was frozen by liquid nitrogen in a Dewar flask and was lyophilized.

f. Peptide Cyclization: The lyophilized powder from step e was solubilized in 0.5 mL DMF and 1.5 eq. each of HATU, HOBt, and 3 eq. of DIPEA (in 1mL DMF) was added to the peptide. The reaction was monitored by UPLC. After 48 hours, DMF was removed in vacuo. The peptide was extracted with EtOAc and was evaporated in rotary evaporator, followed by resolubilizing in 500µL ACN:Water (1:1, v/v with 0.1% TFA) and purified through prep-HPLC, followed by lyophilization.

g. Final Deprotection: The lyophilized cyclopeptide powder was dissolved in 2 mL TFA:TIS:water (95:2.5:2.5, v/v/v). After 2 hrs, the volume was reduced under airflow to

~0.5mL and 10-fold volume of cold ether was added, the suspension was cooled (in dry ice for ~30 min) and centrifuged (4000 rpm, 15 min, 4ºC). Afterwards, the ether phase was decanted. The remaining pellet was dissolved in 1 mL of ACN: water (1:1, v/v with 0.1% TFA) by vortexing. The solution in the falcon was frozen by liquid nitrogen in a Dewar flask and was lyophilized, followed by purification using prep-HPLC. The sequence is shown in Fig. S2. The UPLC trace, along with the mass data, is shown in Fig. S14, and retention time, UPLC gradient, yield is tabulated in table S1.

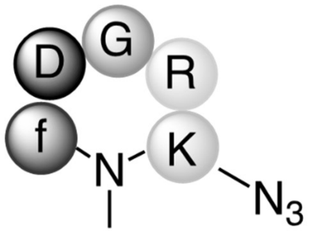

Fig. S2 Amino acid sequences in cyclic RGD.

2.5 Synthesis of GE 11

A fritted syringe reactor was charged with TentaGel® R RAM resin required for a 25 µmol scale . After Fmoc cleavage (20% piperidine in DMF, 800 µl, 2x 5 min) and washing with DMF (3x, 1 mL), the resin was treated with a mixture of 4 eq. of Fmoc-Lys (N_3)-OH, 3.85 eq. HATU and 8 eq. DIPEA in DMF (concentration: 0.2M).

Subsequently, the resin was washed thoroughly with DMF (5x) and attached to the synthesizer for further couplings via automated SPPS (as mentioned in sec 2.2). After completion of peptide assembly, the Fmoc cleavage (20% piperidine in DMF, 800 µl, 2x 5 min) was performed followed by washing with DMF (3x, 1 mL), DCM (5x, 1 mL). The resin was dried under a vacuum. The global deprotection was performed with the 2 mL cleavage cocktail TFA/triisopropylsilane/H_2O 95:2.5:2.5 (v/v/v) at room temp for 2 h. After 2 h the cleavage cocktail was collected by filtration in 15 mL falcon, the resin was washed with the cleavage cocktail (3x 1 mL) and the combined filtrates were concentrated (~ 0.5 mL) under airflow. Et_2O (10-fold volume) was added to the remaining residue, the suspension was cooled (in dry ice for ~30 min) and centrifuged (4000 rpm, 15 min). Afterwards, the ether phase was decanted. The remaining peptide pellet was dissolved in 1 mL of ACN: water (1:1, v/v with 0.1% TFA) by vortexing. The solution in the falcon was frozen by liquid nitrogen in a Dewar flask and was lyophilized. Afterwards, it was purified using RP-HPLC. The UPLC trace, along with the mass

data, is shown in Fig. S15 and retention time, UPLC gradient, yield is tabulated in table S1. The sequence is shown in fig. S3.

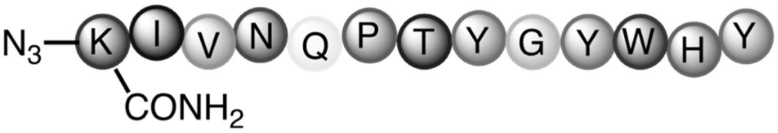

Fig. S3 Amino acid sequences in GE11.

2.6 Synthesis of GE 137

6M Gn.HCl (28.66 g) and 200 mM Na_2HPO_4 (1.4196 g) were solubilized in 50 mL milli q water under stirring. The pH was adjusted to 7 and degassed using argon for further use.

Stock solutions used for synthesizing GE137: (a) 23 mg $PdCl_2$ was dissolved in 200 µL (648.5 mM) 6 M Gn.HCl buffer, pH 7; (b) 22.5 mg dithiocarbamate was dissolved in 100 µL (1 M) water; (c) 30 mg disulfiram was dissolved in 300 µL (336.7 mM) ACN; (d) 15.36 mg glutathione was dissolved in 100 µL (0.5 M) water.

A fritted syringe reactor was charged with TentaGel® R RAM resin frequired for 25 µmol scale. After Fmoc cleavage (20% piperidine in DMF, 800 µl, 2x 5 min) and washing with DMF (3x, 1 mL), the resin was treated with a mixture of 4 eq. of Fmoc-Lys (N_3)-OH, 3.85 eq. HATU and 8 eq. DIPEA in DMF (concentration: 0.2M). Subsequently, the resin was washed thoroughly with DMF (5x) and attached to the synthesizer for further couplings via automated SPPS (as mentioned in sec 2.2). (Stage 1, Fig. S4). After completion of the chain assembly, Fmoc cleavage (20% piperidine in DMF, 800 µl, 2x 5 min) was performed followed by washing with DMF (3x, 1 mL).

The *N*-terminus was acetylated using acetic anhydride: lutidine: DMF (4:4:2, v/v/v, 1 mL) for 20 minutes two times. Subsequently, the resin was washed with DMF (3x, 1 mL), DCM (5x, 1 mL) and dried under vacuum. The global deprotection was performed with the 2 mL cleavage cocktail TFA/triisopropylsilane/H_2O 95:2.5:2.5 (v/v/v) at room temp for 2h. After 2 h the cleavage cocktail was collected by filtration in 15 mL falcon, the resin was washed with the cleavage cocktail (3x 1 mL) and the combined filtrates were concentrated (~ 0.5 mL) under airflow. Et_2O (10-fold volume) was added to the remaining residue, the suspension was cooled (in dry ice for ~30 min) and centrifuged (4000 rpm, 15 min). Afterwards, the ether phase was decanted. The remaining peptide pellet was dissolved in 1 mL of ACN: water (1:1, v/v with 0.1% TFA) by vortexing. The solution in the falcon was frozen by liquid nitrogen in a Dewar flask and was lyophilized. Afterwards, it was purified using RP-HPLC. (Stage 2, Fig. S4). The

lyophilized peptide (10 mg) was dissolved in 400 µL 6 M Gn.HCl buffer pH 7 (8.45 mM), and treated with DSF (75 µL from stock-c) for 5 minutes at 42°C. Then, it was purified via RP-HPLC and lyophilized (Stage 3, Fig. S4).

3 mg from the total lyophilized powder was again solubilized in 400 µL (2.54 mM) 6 M Gn.HCl buffer, pH 7, followed by addition of 10 eq. $PdCl_2$ (~ 16 µL from stock a) for 15 min at 42°C. Then, 50 eq. DTC (~51 µL from stock b) and 10 eq. GSH (~20 µL from stock d) was added and incubated for 15 minutes at 42°C—further, 10 eq. DSF (30 µL, stock c) was added and incubated for another 15 minutes at 42°C (Stage 3, Fig. S4). Subsequently, the peptide was purified using HPLC and lyophilized. The UPLC trace, along with the mass data, is shown in Fig. S16 and retention time, UPLC gradient, yield is tabulated in table S1. The sequence is shown in fig. S4.

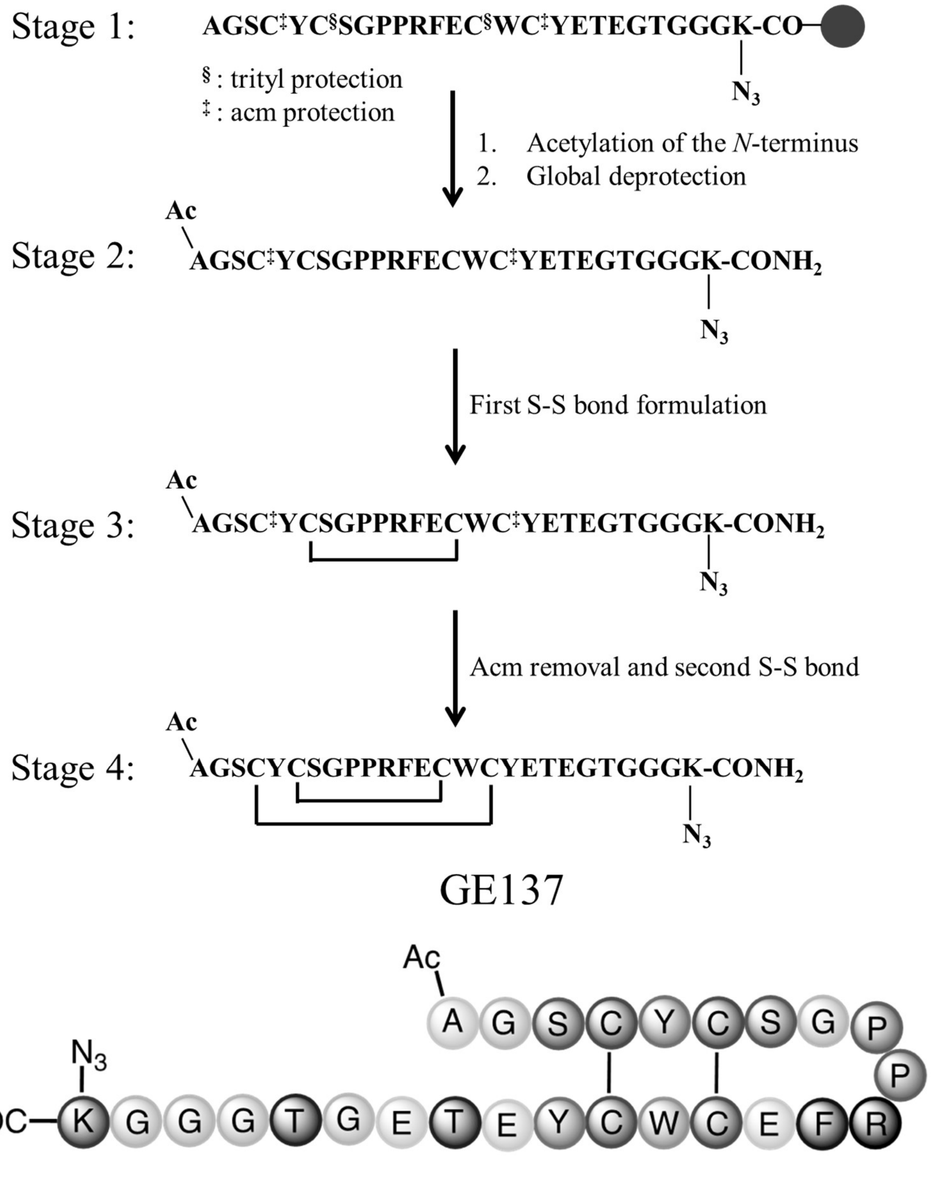

Fig. S4. Synthetic scheme for preparation of GE 137.

2.7 Conjugation of peptides GE 137 and GE11 with TAMRA

Stock solutions were prepared: (a) 32.6 mg THPTA was dissolved in 300 µL water (0.25M); (b) 37.44 mg $CuSO_4.5H_2O$ was dissolved in 300 µL (0.5 M) water; (c) 26.42 mg ascorbic acid was dissolved in 300 µL water (0.5M); (d) 2.25 mg GE 137 in 200 µL (4 mM); (e) 1.35 mg GE 11 in 200 µL (4 mM); (f) 1.40 mg TAMRA-Alkyne in 500 µL (6 mM) (Fig. S5 for the conjugate structure).

In a 100 µL eppendorf vial, 4.8 µL metal ion (from stock b) and 9.6 µL ligand (from stock a) were mixed and kept in shaker for 15 minutes at room temperature. Addition of the ligand immediately changed the color of the solution from light green to deep blue. After 15 minutes, a stoichiometric amount of ascorbic acid (4.8 µL from stock c) was added to this mixture to reduce the Cu^{2+} to Cu^{+}. The mixture was kept for 15 minutes at room temperature. Adding ascorbic acid to the mixture immediately reduces Cu^{2+}, making the solution colorless. In two individual reaction sets, 30 µL of peptide (either from stock d or e) and TAMRA (stock f) were mixed and kept at 50 °C for 5 hrs. Then, a concentrated solution of EDTA (200 µL, 1M, pH 7) was added to the reaction and kept at room temperature for 15 minutes. This solution was purified via RP HPLC and characterized via UPLC and MS analysis (Fig. S17 for GE11TAMRA and S18 for GE137TAMRA), and retention time, UPLC gradient, yield is tabulated in table S1. The TAMRA-alkyne, GE11TAMRA and GE137TAMRA conjugates are shown in fig. S5.

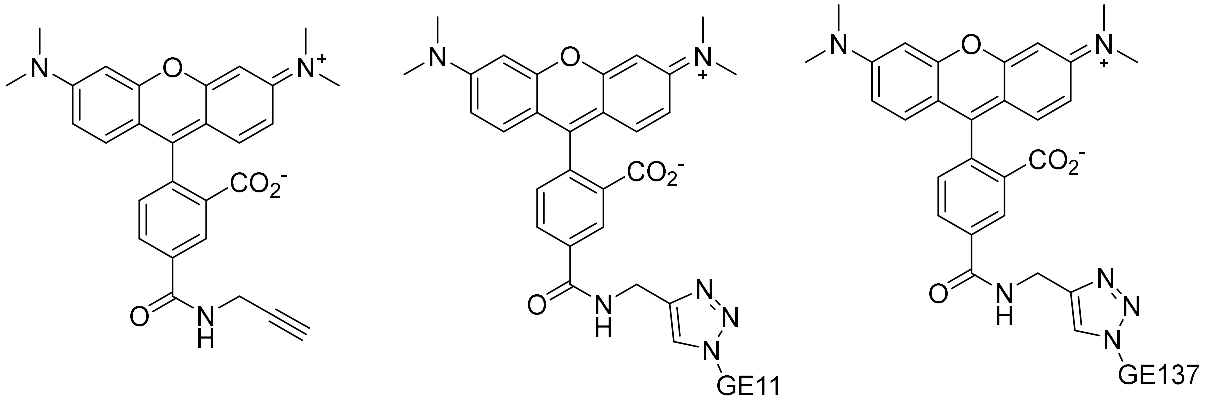

Fig. S5 TAMRA Alkyne (left), GE11TAMRA (middle) and GE137TAMRA (right).

2.8 Conjugation of peptides Cyclic L1 and Cyclic RGD with AF568

The peptides (cyclic L1 and cyclic RGD) were labeled with AF568 via copper click reaction. Reaction mixture (100 µL) included 200 nmol of AF568-alkyne, 200 nmol of peptide, 6 µmol sodium ascorbate, 600 nmol of copper II sulfate, and 3 µmol of THPTA. Initially the click reactants are mixed (copper II sulfate, THPTA and sodium ascorbate) followed by addition of the peptide and the dye. The reaction was incubated in the dark at 55 °C for 2h. AF568-Alkyne, AF568-Cyclic L1 and AF568-Cyclic RGD conjugates are shown in fig. S6. This solution was purified via RP HPLC and characterized via UPLC and MS analysis (Fig. S19 for AF568-Cyclic L1 and S20 for AF568-Cyclic RGD), and retention time, UPLC gradient, yield is tabulated in table S1.

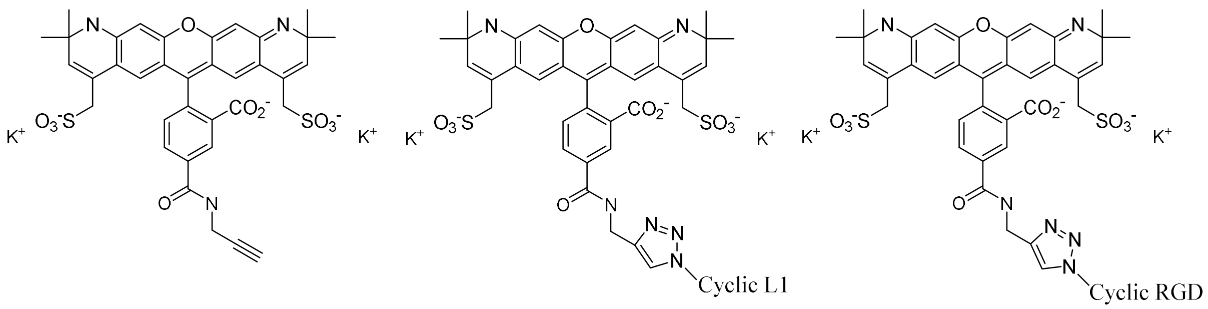

Fig. S6 AF568-Alkyne (left), AF568-Cyclic L1 (middle) and AF568-Cyclic RGD (right).

2.9 DNA modifications

2.9.1 Template strand with MMAE

0.1 M phosphate buffer (pH 7.4) was degassed by purging argon and freezing in liquid nitrogen. The thiol-modified template strand was incubated with 100 mM tris(2-carboxyethyl) phosphine (TCEP) for 30 min at 42°C in 0.1 M phosphate buffer (pH 7.4) to reduce possible disulfide bonds. Subsequently, the maleimide functionalized vcMMAE dissolved in DMSO (0.5 M) was added in 10-fold molar excess. The final concentration of the oligonucleotide in the reaction mixture was 200 µM. The reaction was allowed to proceed overnight at 37 °C. The product was purified by HPLC, freeze-dried, and stored at −20 °C until use. During use, the DNA was dissolved in water, and the final concentration was determined photometrically at 260 nm assuming extinction coefficients calculated by https://molbiotools.com/dnacalculator.php.

2.9.2 Conjugation of (cyclo)peptides with oligonucleotides

Stock solutions were prepared: (a) 32.6 mg THPTA was dissolved in 300 µL water (0.25M); (b) 37.44 mg $CuSO_4.5H_2O$ was dissolved in 300 µL (0.5 M) water; (c) 26.42 mg ascorbic acid was dissolved in 300 µL water (0.5M); (d) isopropanol 1.5mL, 3M ammonium acetate 100 µL and water 100 µL were mixed and kept in freezer for at least one hour prior to use.

A 100 µL Eppendorf vial was charged with 4.8 µL from stock (b) and 9.6 µL from stock (a). The mixture was kept in a shaker for 10 minutes at room temperature. Addition of the ligand immediately changed the color of the solution from light green to deep blue. After 15 minutes, a stoichiometric amount of ascorbic acid (4.8 µL from stock c) was added to this mixture to reduce the Cu^{2+} to Cu^{+} and kept for further 10 minutes at room temperature. Adding ascorbic acid to the mixture immediately reduces Cu^{2+}, making the solution colorless.

Into this copper-THPTA complex solution, 0.8mM oligonucleotide was added. Subsequently, the peptide (4 mM, dissolved in DMSO) was added in 5-fold molar excess. This mixture was kept at 50 °C for 5 hrs. Then, a concentrated solution of EDTA (200 µL, 1M, pH 7) was added to the reaction and kept at room temperature for 15 minutes. This mixture was transferred into the stock (d). The solution was kept at −20 °C overnight. The next day, the solution was centrifuged for 10 minutes at 10.000 RPM. The supernatant was removed. The pellet was dissolved in water and lyophilized. Then, 200 µL water was added and the mixture filtered through a 0.22 µm syringe filter. The filtrate was submitted to HPLC purification. Product containing fractions were freeze-dried and stored at −20 °C until use. During use, the conjugate was dissolved in water, and the final concentration was determined photometrically at 260 nm assuming extinction coefficients calculated by https://molbiotools.com/dnacalculator.php. The UPLC traces and Maldi data for respective DNA scaffolds are shown in Fig. S21-S31.

2.9.3 dsDNA complex formation

Template strand and shorter strands (conjugated with peptide) were mixed in equimolar stoichiometry with ratio 1:1:1, final conc. 10 µM in Milli-Q® water; annealed at 90 °C for 2 minutes then cooled down to RT. This stock solution was diluted with buffer used for the cell experiments. The complexation was verified in two instances by Native PAGE (Section 2.9.4).

2.9.4 Native Polyacrylamide Gel Electrophoresis (native PAGE)

Bispecific DNA complexes were characterized through native polyacrylamide gel electrophoresis (native PAGE) at a concentration of 20% acrylamide/bisacrylamide (19:1 v/v). All samples were loaded at a concentration of 100 ng/µl upon mixing with 6x purple gel loading

dye (New England Biolabs). The electrophoresis was performed in 1x TBE buffer at a constant voltage of 100 V for 1h at room temperature. Firstly, the gels were imaged via in-gel fluorescence detection based on the Atto565 emission. Subsequently, the gel was stained with SYBR Gold (1:10,000) in 1x TBE buffer for 15 min while gently rocking at room temperature and were imaged again (Gel Doc XR+, Bio-Rad), see Fig. S35.

2.9.5 MALDI-TOF mass spectrometry

MALDI-TOF mass spectra were measured on a Shimadzu Axima Confidence spectrometer (Shimadzu, Kyoto, Japan) in positive mode. Diammonium hydrogen citrate (AHC) in ultra-pure water (100 mg/mL) and 3-hydroxypicolinic acid (HPA) in ultra-pure water:ACN (1:1, v/v) with a concentration of 50 mg/mL were prepared. Then HPA: AHC=10:1, v/v was prepared and used further in matrix. Samples were measured in positive mode with an ion gate of 5000 Da and pulsed extraction adjusted to the respective analyte mass. The laser was operated at 50 Hz and a power of 90-120. Between 200 and 600 single shots were accumulated per mass spectrum. The recorded spectra were calibrated with the single protonated ion signals of the oligonucleotide calibration standard (Bruker Daltonics, Bremen, Germany).

2.9.6 Characterization data

Table S1. Characterization data for the peptides. Mobile phase A (98.9 % H_2O, 1 % ACN, 0.1 % TFA) and B (98.9 % ACN, 1 % H_2O, 0.1 % TFA), λ = 210 nm (3-80% solvent B in 6 min)

Peptide	Gradient	t_R	Calculated mass/mass obtained (m/z used)	yield
Cyclic L1	λ = 210 nm (3-80% solvent B in 6 min)	3.23 min	calculated mass: 2122.38 (+1), 1061.69 (+2); found: 1061.55	43%
cyclic RGD	λ = 210 nm (3-80% solvent B in 6 min)	2.33 min	calculated mass: 644.33 (+1); found: 644.4.	35%
GE11	λ = 210 nm (3-80% solvent B in 6 min)	2.39 min	calculated mass: 1694.89 (+1), 847.95 (+2); found: 848.06.	53%
GE137	λ = 210 nm (3-80% solvent B in 6 min)	2.47 min	calculated mass with two S-S bonds 2810.09 (+1), 1405.55 (+2), 937.37 (+3); found: 937.3 and 1405.5.	24%
GE11-TAMRA	λ = 210 nm (3-80% solvent B in 6 min)	2.67 min min	calculated mass 1081.21(+2), 721.14 (+3), 541.11 (+4); found: 1082.06, 721.65, 541.87.	76%
GE137-TAMRA	λ = 210 nm (3-80% solvent B in 6 min)	2.29 min	calculated mass 1093.03 (+3); found: 1092.93.	68%
L-AF568	λ = 280 nm (3-80% solvent B in 6 min)	2.64 min	calculated mass 951.72 (+3); found: 951.97.	n.d.
C-AF568	λ = 280 nm (3-80% solvent B in 6 min)	2.52 min	calculated mass 688.27 (+2); found: 688.79	n.d.

Table S2 Characterization data for the DNA-peptide conjugates. A (0.1M triethylammonium acetate pH: 7.5) and B (acetonitrile) were used. λ = 260 nm, (3-30% solvent B in 6 min). Conjugation sites are marked with an asterisk.

DNA-Peptide	Gradient	t_R	Calculated mass/mass obtained (Molecular mass is used)	yield
$3'_1L$<br>Alkyne modification for 3′ end	λ = 260 nm (3-30% solvent B in 6 min)	2.49 min	calculated mass: 8732, found: 8733.12	60%
DNA Strand 1:<br>3′-C*GCATTCCTCTAGACCTTACT-5′				
$5'_1L$<br>Alkyne modification for 5′ end	λ = 260 nm (3-30% solvent B in 6 min)	2.49 min	calculated mass: 8732, found: 8731.12	62%
DNA Strand 1:<br>3′-CGCATTCCTCTAGACCTTACT*-5′				
$3'_2C$<br>Alkyne modification for 3′ end	λ = 260 nm (3-30% solvent B in 6 min)	2.27 min	calculated mass: 7070, found: 7070.20	64%
DNA Strand 2:<br>3′-C*AGCTCGAGGTATTATTCGC-5′				
$5'_2C$<br>Alkyne modification for 5′ end	λ = 260 nm (3-30% solvent B in 6 min)	2.27 min	calculated mass: 7070, found: 7069.03	58%
DNA Strand 2:<br>3′-CAGCTCGAGGTATTATTCGC*-5′				
$3'_2$ S<br>Alkyne modification for 3′ end	λ = 260 nm (3-30% solvent B in 6 min)	2.10 min	calculated mass: 8121, found: 8122.45	65%
DNA Strand 2:<br>3′-C*AGCTCGAGGTATTATTCGC-5′				
$5'_2$ S<br>Alkyne modification for 5′ end	λ = 260 nm (3-30% solvent B in 6 min)	2.27 min	calculated mass: 8121, found: 8123.01	67%
DNA Strand 2:				

3′-CAGCTCGAGGTATTATTCGC*-5′				
$3'_1$ O Alkyne modification for 3′ end	λ = 260 nm (3-30% solvent B in 6 min)	1.17 min	calculated mass: 9420.09, found: 9420.89	60%
DNA Strand 1: 3′-CGCATTCCTCTAGACCTTACT-5′				
$5'_1$ O Alkyne modification for 5′ end	λ = 260 nm (3-30% solvent B in 6 min)	1.13 min	calculated mass: 9420.09, found: 9420.72	58%
DNA Strand 1: 3′-CGCATTCCTCTAGACCTTACT*-5′				
$3'_2$ C2 Alkyne modification for 3′ end	λ = 260 nm (3-30% solvent B in 6 min)	2.43 min	calculated mass: 7904.8, found: 7904.14	n.d.
DNA Strand 2: 3′-C*AGCTCGAGGTATTATTCGC-5′				
$3'_2$ C3 Alkyne modification for 3′ end	λ = 260 nm (3-30% solvent B in 6 min)	2.70 min	calculated mass: 8758.2, found: 8763.71	n.d.
DNA Strand 2: 3′-C*AGCTCGAGGTATTATTCGC-5′				
Template strand with vcMMAE at 5´ where 5´ was modified with Thiol-C6	λ = 260 nm (3-30% solvent B in 6 min)	3.30 min	Calculated mass: 14259.6, found: 14259.73	82%
DNA Template Strand 5´-GCGTAAGGAGATCTGGAATGA-GTCGAGCTCCATAATAAGCG-3´				

Alkyne modification for 3′ end:

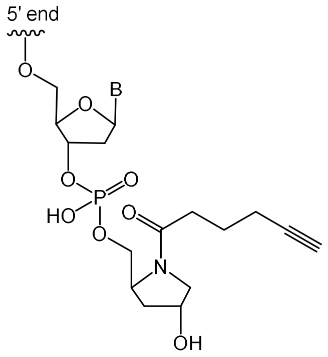

Alkyne modification for 5′ end:	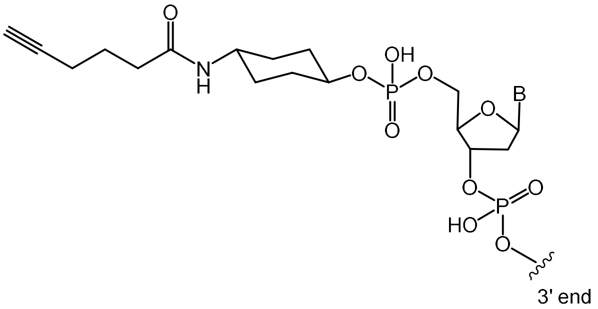
5′ Thiol-C6:	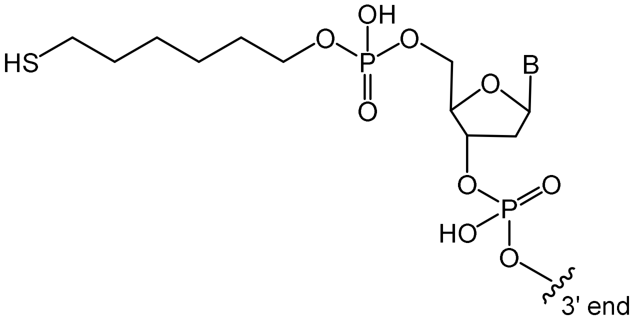

3. Cell Culture and Biological Experiments

3.1 Reagents, media for cell culture

Dulbecco's Modified Eagle medium (DMEM) High Glucose, Ham's F12 medium and RPMI 1640 medium were purchased from Biowest (Nuaillé, France). Fetal Bovine Serum (FBS), SYTOX™ Blue Dead Cell Stain, alamarBlue™ Cell Viability Reagent, StemPro™ Accutase™ Cell Dissociation Reagent, Poly-D-Lysine (0.1 mg/ml) and Hoechst 33342 (10 mg/ml), were obtained from Thermo Fisher Scientific (Waltham, USA). Integrin β3/ITGB3/CD61 siRNA was obtained from Santa Cruz (sc-29375). Opti-MEM™ reduced serum medium with GlutaMAX™ (Gibco™), Lipofectamine™ RNAiMAX (Invitrogen™) and Alexa Fluor® 488 anti-human CD51/61 antibody and APC anti-human CD309 (VEGFR2) antibody from Biolegend. McCoy's 5A was purchased from VWR (L0210-500, BioWest). Accutase® enzymes in Dulbecco's PBS containing 0.5 mM EDTA (without calcium and magnesium) was purchased from Biolegend, California, USA. Sterile 10x PBS pH 7.4 without Ca/Mg was purchased from Carl Roth GmbH (Karlsruhe, Germany). Endothelial cell growth medium (EGM) includes basal medium and supplement mix (catalogue number: C-22010) Promocell. SYBR gold was purchased from Thermo Fisher. A549 and HUVEC cells were obtained from ATCC (Virginia, USA) and pre-screened single donor C-12205 Promocell. Trypsin-EDTA 1X

in PBS without Ca/Mg without Phenol Red was purchased from VWR International GmbH (Darmstadt, Germany). PenicillinStreptomycin (10,000 U/mL) was obtained from MP Biomedicals Germany GmbH (Eschwege, Germany). µ-Slide 8 Well microscopy slides were purchased from ibidi GmbH (Gräfelfing, Germany). Perkin Elmer Victor and BMG plate readers were used in fluorescence channel for alamar blue^{TM} as suggested in the vendor protocol. HEPES was obtained from Sigma. NaCl and $CaCl_2$ were obtained from Sigma. FITC anti-human CD326 (EpCAM) antibody for HCT-116 cells was obtained from Biolegend and used as suggested in the vendor protocol. Sytox Red was obtained from ThermoFischer and used as suggested in the vendor protocol. Annexin-V-FITC and PI were obtained from ThermoFischer and used as suggested in the vendor protocol. Annexin-V-Alexa Fluor 647 was obtained from Biolegend and used as suggested in the vendor protocol.

3.2 Cell culture and experiments with cells

3.2.1 Preparing the medium

The EGM medium (supplemented with 2% FBS, ECGS, VEGF, EGF, FGF, IGF, ascorbic acid, heparin and hydrocortisone) containing no antibiotics was used for culturing HUVEC cells and was always prepared fresh. For culturing A549 cells, DMEM/Ham's F12 1:1 (v/v) with 10% FBS and 1% penicillin/streptomycin was prepared. McCoy's 5A, containing 10% FBS and 1% penicillin/streptomycin, was used for HCT-116 cells. HEK-293 and A498 cells were cultured in DMEM with 10% FBS and 1% penicillin/streptomycin. The medium was always preheated to 37°C before use.

3.2.2 Thawing cells

Before thawing the cells, a T75 culture flask was charged with 10 mL medium and warmed to 37°C in the incubator. The cryovial was taken from the nitrogen tank to room temperature and transferred to a prewarmed water bath at 37°C for thawing. The cell suspension was transferred to a centrifuge tube containing pre-warmed medium and spin down at 200xg for 5 minutes to wash off residual freezing medium containing DMSO. Then, the resuspended cells were transferred to the prepared T75 culture flask and kept in an incubator with 37°C and 5% CO_2. At a confluence between 80-90%, the cells were passaged.

3.2.3 Culture of HUVEC cells

Before starting, the EGM medium was warmed at 37°C. The medium was removed from the culture flask, and 5 mL PBS was added and aspirated to wash the cells. Trypsin/EDTA (0.04 %, 1 mL) was added, and the flask was kept in the incubator at 37°C for 5 minutes. Afterwards, trypsin inhibitor (1 mL) and EGM medium (3 mL) were added to the flask, and the cell suspension was transferred to a 15 mL falcon tube. The cells were spun down for 5 minutes at 200 x g. The pellet was carefully re-suspended in fresh medium. After counting the cells, one part of the suspension was seeded for further culturing, while another portion was used for experiments.

3.2.4 Culture of A549 cells

A549 cells were cultured in DMEM/Hams F12 1:1 (v/v) with 10% FBS and 1% penicillin/streptomycin at 37°C and 5% CO_2. Once confluency reached between 85-95%, the supernatant was aspirated, and cells were washed with PBS pH 7.4 to remove the residual medium. For cell detachment, 1 mL accutase was added, and the cells were incubated at 37°C with 5% CO_2 for five minutes. To stop the accutase activity, fresh medium was added, and the A549 cells were transferred to a 15 ml tube and centrifuged. Passages 6-8 were used during analysis. After counting the cells, one part of the suspension was seeded for further culturing, while another portion was used for experiments.

3.2.5 Culture of HCT-116 cells

HCT-116 cells were cultured in McCoy's 5A with 10% FBS and 1% penicillin/streptomycin (1x Pen/Strep; 100x stock contains 10.000U/ml Penicillin+ 10.000µg/ml Streptomycin) at 37°C and 5% CO_2. Once confluency was between 85- 95%, the supernatant was removed, and cells were washed once with 1x PBS pH 7.4. Trypsin/EDTA (0,25 %, 1 mL) was added, and the flask was kept in the incubator at 37°C for 5 minutes. Afterwards, 1 mL trypsin inhibitor and 3 mL medium were added. The cell suspension was centrifuged 200xg for 5min, and the pellet was resuspended in fresh culture media. After counting the cells, one part of the suspension was seeded for further culturing, while another portion was used for experiments.

3.2.6 Culture of HEK-293 and A498 cells

HEK-293 and A498 cells were cultured in DMEM with 10% FBS and 1% penicillin/streptomycin (1x Pen/Strep; 100x stock contains 10.000U/ml Penicillin+

10.000µg/ml Streptomycin) at 37°C and 5% CO_2. Once confluency was between 85- 95%, the supernatant was removed, and cells were washed once with 1x PBS pH 7.4. Trypsin/EDTA (0.25 %, 1 mL) was added, and the flask was kept in the incubator at 37°C for 5 minutes. Afterwards, 1mL trypsin inhibitor and 3mL medium were added. The cell suspension was centrifuged 200xg for 5min, and the pellet was resuspended in fresh culture media. After counting the cells, one part of the suspension was seeded for further culturing, while another portion was used for experiments.

3.2.7 Culture of Hek-Gly2Rα-GFP cells

Hek-Gly2Rα-GFP cells were cultured in DMEM with 10% FBS and 1% penicillin/streptomycin (1x Pen/Strep; 100x stock contains 10.000U/ml Penicillin+ 10.000µg/ml Streptomycin) at 37°C and 5% CO_2. Puromycin, 4 µg/ml concentration (stock 10mg/ml), was added to control that stable transfected cells still contain target gene after long time storage in liquid nitrogen.

Once confluency was between 85-95%, the supernatant was removed, and cells were washed once with 1x PBS pH 7.4. Trypsin/EDTA (0.25 %, 1 mL) was added, and the flask was kept in the incubator at 37°C for 5 minutes. Afterwards, 4ml culture medium was added to stop trypsin activity. The cell suspension was centrifuged 200xg for 5min, and the pellet was resuspended in fresh culture media. After counting the cells, one part of the suspension was seeded for further culturing, while another portion was used for experiments.

3.2.8 Permanent transfection of HEK293 cells with eGFP-MS2BP

A HEK-293 cell line was constructed that stably expresses a fusion protein comprising the enhanced Green Fluorescent Protein (eGFP) and the MS2 Binding Protein (MS2BP). This cell line was initially designed for testing mRNA imaging technologies, and was then repurposed for the investigations described herein. The DNA construct (Fig. S7) further contained a segment related to the sequence of Glycin Receptor Protein 2α (GLRP) termed 4NE.GLRP and tagged with 9 copies of the MS2 hairpin. The eGFP-MS2BP was connected to the GLRP-MS2 through a Nuclear Localization Sequence (NLS) and the "self-cleaving" P2A sequence. A stop codon in the GLRP sequence prevents translation of GLRP-MS2 (Fig. S7B). The eGFP-MS2BP-NLS-P2A protein localizes to the nucleus where it can bind the GLRP-MS2 mRNA via the MS2·MS2BP interaction. This interaction is, however, irrelevant for this study, which

required a HEK293 cell line that can be distinguished from HUVECs by fluorescence in the green channel.

To expedite the experimental workflow, the designed sequence (Fig. S8) eGFP-MS2BP-NLS-P2A-4NE.GLRP-MS2 (ID: U2537EL030-3)) was customized into a pBluescript II SK(-) vector by Genescript (Piscataway, USA). For later restriction and ligation into a pAAVS1-puro-DNR vector (GE100024, OriGene) for knock-in transfection, the customized sequence was equipped with overhangs compatible with KpnI and NotI restriction sites to enable transfer by restriction ligation into the pAAVS1-puro-DNR vector (Fig. S8), which provides the pAAVS1-eGFP-MS2BP-NLS-P2A-4NE.GLRP-MS2 vector (Fig. S9).

The stable cell line was generated using HEK293 cells by co-transfection (Fig. S10) of the previously designed transgene knock-in vector and pCas-Guide-AAVS1 (GE100023, OriGene) for targeted insertion into AAVS1 locus (Adeno-associated virus integration site 1, human Chromosome 19). For transfection of 1 μg of plasmid DNA, Lipofectamine LTX plus from Invitrogen (Carlsbad, USA) was used. Cells were incubated with the Lipofectamine-DNA complex for 24h. The transfected cells were maintained in Dulbecco's Modified Medium (DMEM) supplemented with 10% Fetal Bovine Serum (FBS) and 1x Penicillin/Streptomycin. After two weeks of antibiotic selection with 4μg/ml Puromycin (Invivogen, France), cells were sorted using BD FACS Melody (Becton Dickinson) measuring the GFP expression level. After sorting, a single-cell dilution was performed and cultured in several 96-well culture plates (CellStar, GreinerBioOne). Individual clones were isolated and observed via fluorescent microscopy (Olympus IX83 microscope). The GFP expression of six clones was quantified by quantitative real-time PCR (BioRad) from isolated mRNA (RNeasy Plus Mini Kit, QIAGEN, 74134), which was reverse- transcripted using iScript™ cDNA Synthesis Kit (BioRad, 1708890). qPCR was performed by using the FAST SYBR GREEN Master Mix (ThermoFisher, 4385612) and primers (forward primer: acgtaaacggccacaagttc, reverse primer: aagtcgtgctgcttcatgtg) targeting eGFP (Fig. S11). Based on the qPCR data, clone F12 was selected. Fluorescence microscopic analysis of this close is shown in Fig. S12.

A)

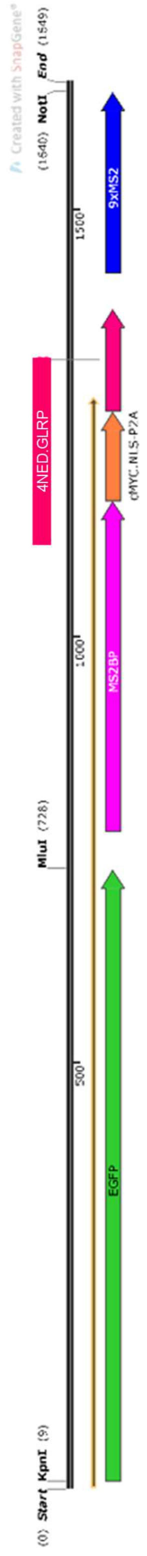

B)

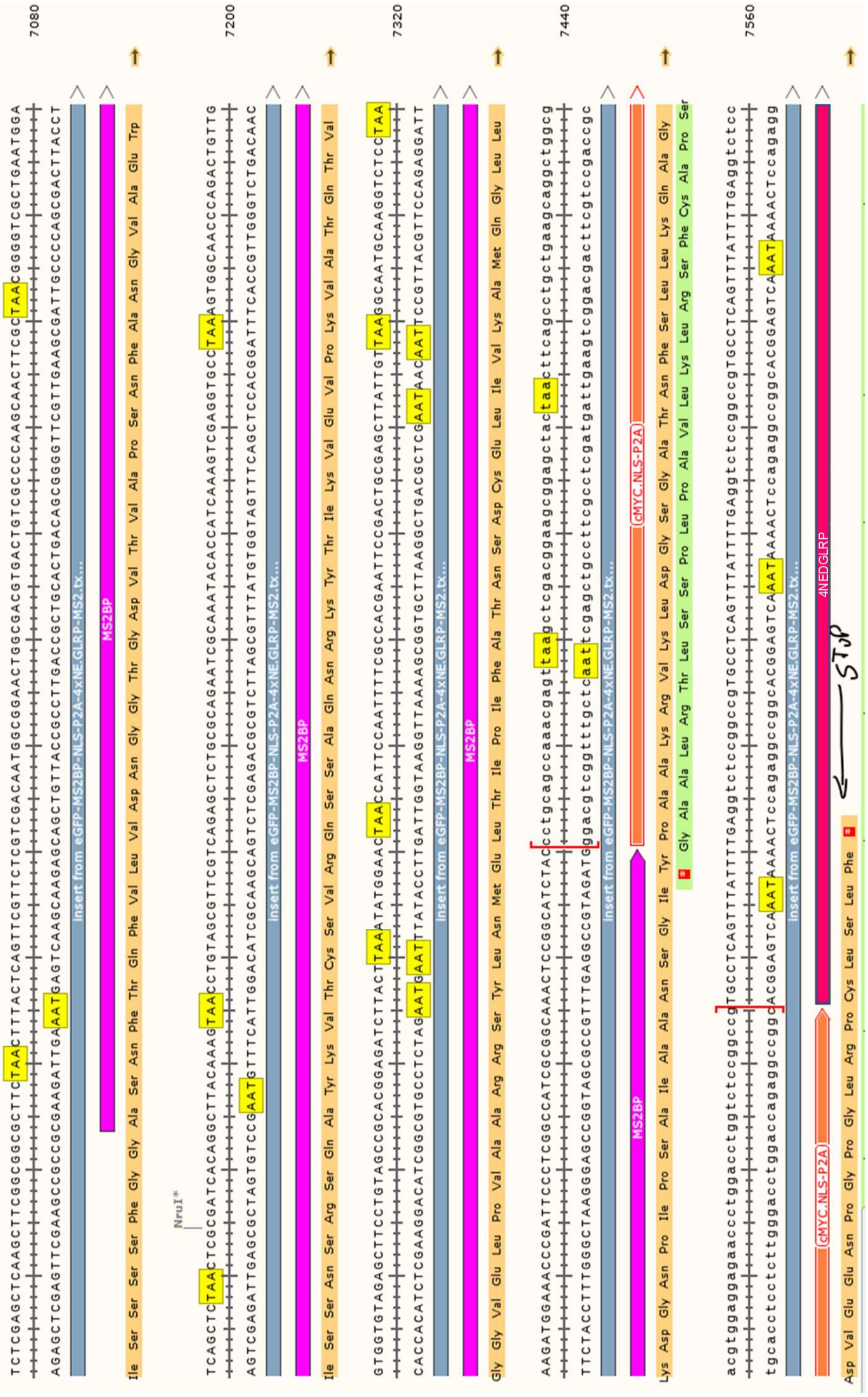

Fig. S7. A) DNA construct designed for creation of a HEK-293 cell line stably expressing the eGFP-MS2BP fusion along with MS2-tagged 4NE-GLRP mRNA. B) Sequence.

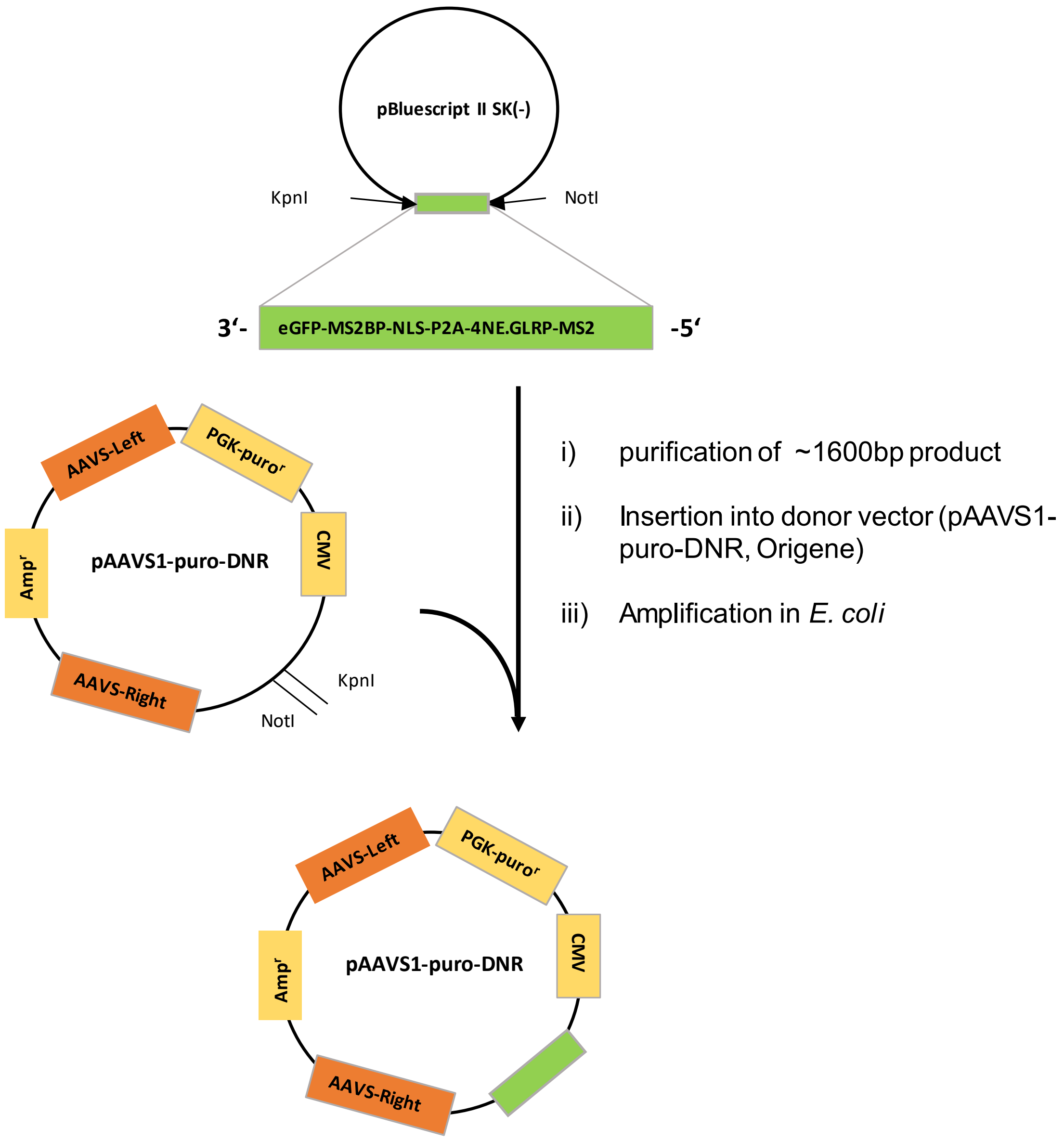

Fig. S8. Scheme for construction of a pAAVS1-eGFP-MS2BP-NLS-P2A-4NE.GLRP-MS2 donor vector for targeted insertion into an AAVS1 site.

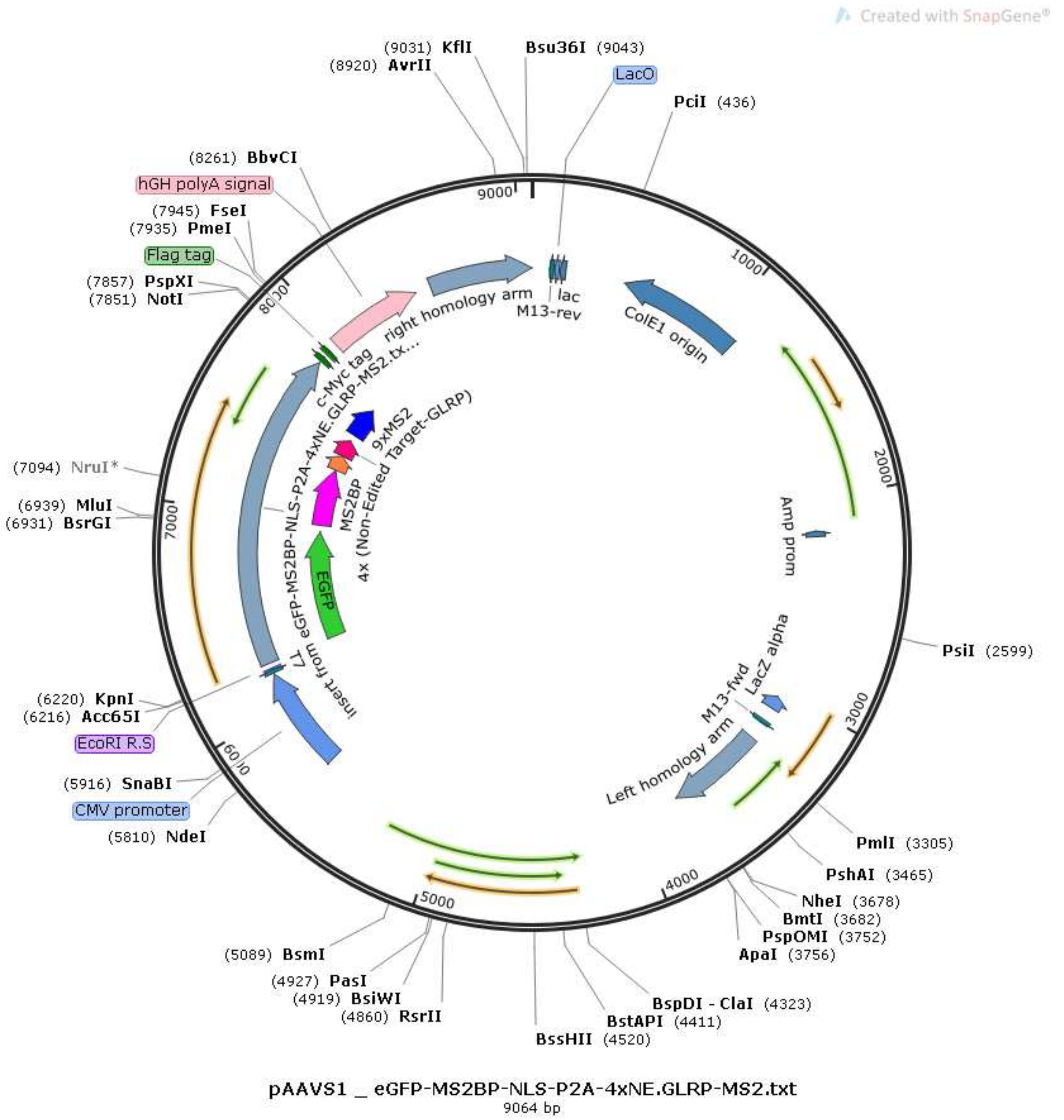

Fig. S9. Map of the plasmid used for transfection of HEK-293 cells.

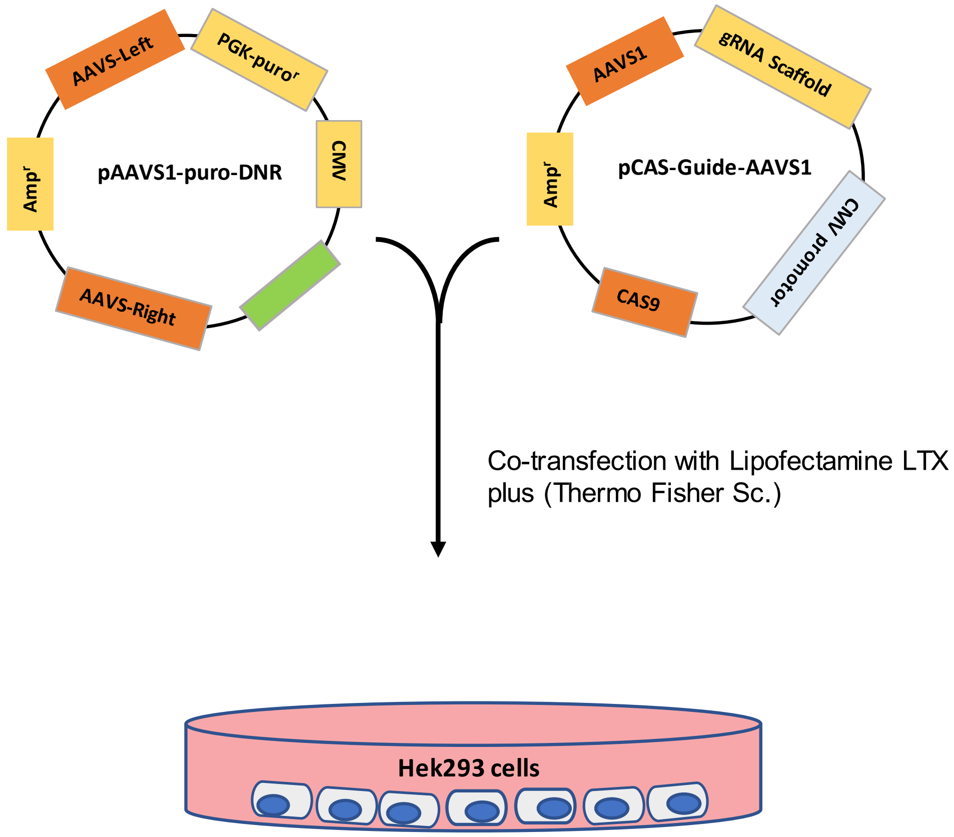

Fig. S10. Scheme for pCAS-Guide-mediated transfection of HEK293 cells. The green segment contains the sequence for the eGFP-MS2BP-NLS-P2A-4NE.GLRP-MS2 construct leading to expression of the eGFP-MS2BP-NLS-P2A fusion protein.

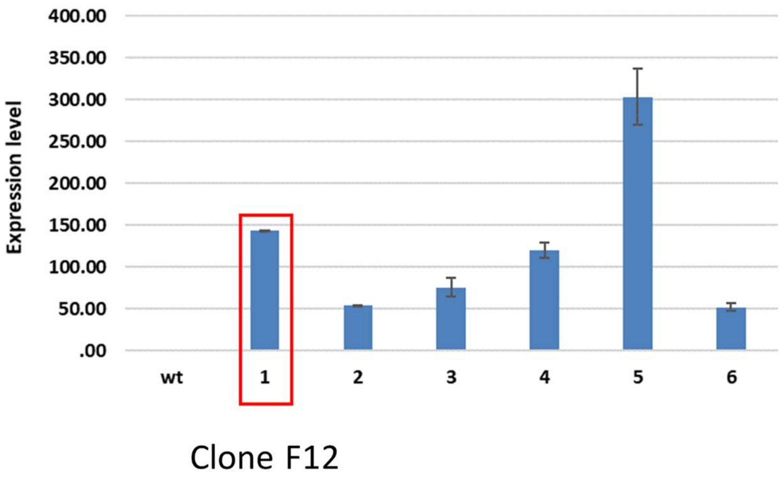

Fig. S11. Comparative characterization of eGFP expression levels for six different clones based on qPCR of total mRNA. Data is from three independent replicates at different days. Clone F12 was selected due to high eGFP expression levels and high stability of eGFP expression (estimated by low standard deviation).

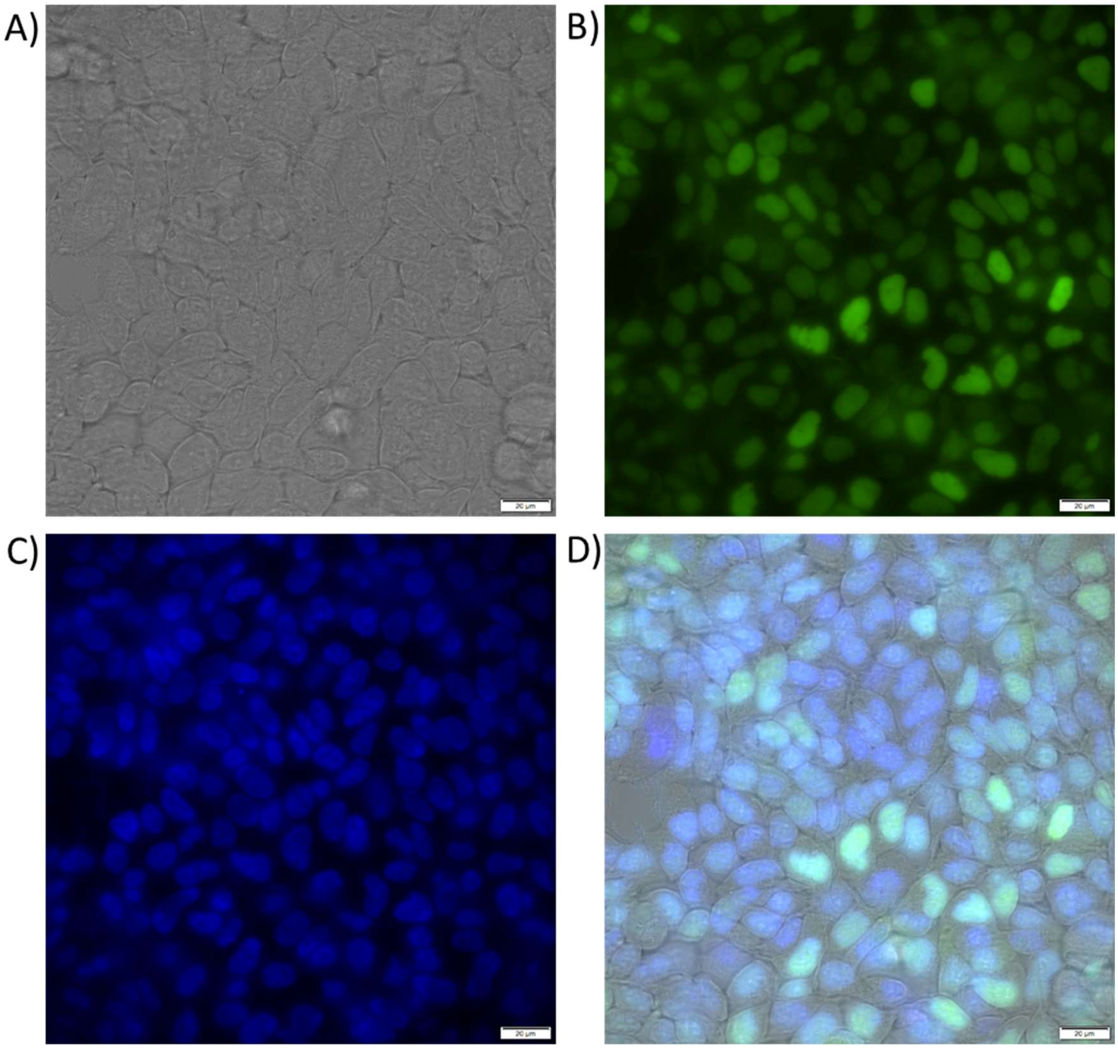

Fig. S12. Fluorescence microscopy image of HEK293 cells stably expressing eGFP-MS2BP (clone F12) with nuclear localization due to a NLS sequence; A) BF channel; B) green channel; C) blue channel; D) all channels merged; Blue: nuclear labelling with Hoechst33342, $\lambda_{ex} = 350 \pm 50$ nm, $\lambda_{em} = 460 \pm 50$ nm; Green: $\lambda_{ex} = 500 \pm 12$ nm $\lambda_{em} = 545 \pm 20$ nm; Scale bar is 20 µm.

3.3 Flow cytometry

As explained in sections 3.2.3-3.2.6, after counting the cells, one part of the suspension in respective full medium was seeded for further culturing, while another portion was used for experiments. This second portion (while the cells are in suspension in respective culture medium) was used in flow cytometry. Here this cell suspension was centrifuged 200xg for 5 min and the cell pellet was resuspended in DNA-peptide conjugate for incubation. Flow cytometry experiments were performed on a BD Accuri™ C6 (BD Biosciences). Excitation lasers: YFP, 488 nm; Atto647N, 640 nm. Emission filters: YFP, 533 ± 15 nm; Atto647N,

675 ± 12.5 nm. 10000 cells were counted in each case and the median value was taken from the instrument, which was used further for plotting data. The data was further processed using FlowJo™ software. The gating strategy is provided for all the cell lines in Fig. S32.

3.3.1 Flow Cytometry with DNA-free peptides

For Cyclic L1 (**L**) and Cyclic RGD (**C**): AF568-Cyclic L1/Cyclic-RGD (final conc. 250 nM) was added to the HUVEC cells at room temperature for 30 min. Competing peptides (without AF568) were added (80 µM for **L** and 40 µM for **C**), and the solution was incubated for another 30 min, then the cells were washed and measured in HBSS.BB buffer (Fig. S33).

For GE 137 (**O**) and GE 11 (**S**): TAMRA-labelled peptides (200 nM in PBS) were separately added in two independent experiments to A549 cells. After 10 minutes of incubation at 37°C the cells were centrifuged and measured in PBS. After in new experiments, dye-free peptides at 500 nM were added to the cells and incubated for 10 minutes at 37°C, then centrifuged, and the medium was removed. Afterwards, peptide-TAMRA (200 nM, PBS) was added and incubated for 10 minutes at 37°C, centrifuged and measured in PBS. As a control, cells were also incubated in PBS for 20 minutes and measured (Fig. S34).

For DNA-Peptide conjugate: Solutions of DNA-peptide conjugates are prepared at 10 µM concentration as stock in MilliQ water and subsequently diluted in PBS. Cells are taken as suspension in respective culture medium which was centrifuged 200xg for 5min and the cell pellet was resuspended with DNA-peptide conjugate solution in PBS, incubated at 37°C, 5%CO_2 for 10 minutes. After 10 minutes, the cells are centrifuged 200xg for 5min, washed carefully with PBS once by resuspension. Further centrifuged 200xg for 5min and resuspended in PBS for the experiments.

Experiments with Antibody using flow cytometry: Alexa Fluor® 488 anti-human CD51/61 antibody for integrins and APC anti-human CD309 (VEGFR2) antibody were used for experiments. For Alexa Fluor® 488 anti-human CD51/61 antibody, the signal was measured at FITC channel (Excitation lasers: YFP, 488 nm; Emission filters: YFP, 533 ± 15 nm) whereas for anti-human CD309 (VEGFR2) antibody the signal was measured in APC channel (Excitation lasers: Atto647N, 640 nm; Atto647N, 675 ± 12.5 nm). 5 µL per million cells in 100 µL of (a) APC anti-human CD309 (VEGFR2) Antibody and (b) Alexa

Fluor® 488 anti-human CD51/61 Antibody (targeting $\alpha_v\beta_3$) were incubated on ice in HBSS.BB buffer for 15 min in the dark (both antibodies were from BioLegend®).

3.4 Fluorescence Microscopy and Image Characterizations

Widefield fluorescence microscopy experiments were performed by using an IX83 microscope from Olympus using a 60x magnification lens. Cells were seeded in µ-slides (ibidi). 50000 cells per well were seeded and used in 48 hours. The channels used were: Blue (Hoechst33342): λex = 350 ± 25 nm, λem = 460 ± 25 nm; Red (TAMRA and Atto-565): λex = 575 ± 12 nm, λem 628 ± 20 nm. For Fig. S42, the image was deconvoluted using the cellSens dimension software and the 2D deconvolution function. Cell Sens Dimension V1.17 (Olympus) software was used for image analysis.

3.4.1. HUVEC and HEK-293 with LC21, 40 and 82

Cells (50.000 per well) were incubated for 48 hrs on µ-slides (ibidi). The medium was removed and the cells were washed with PBS (preheated at 37°C) twice and incubated in HBSS.BB (1x HBSS, 1% BSA, 0,5 mg/mL DNA) for 1h in the fridge (4°C). Probes (500 nM in 200 µL) were added and incubated further for 1 h in the fridge. Subsequently, cells were washed with HBSS buffer for three times. Nucleus staining was done with Hoechst 33342 (2 µg/ml, 200 µL) at room temperature for 10 min. Cells were washed with PBS once and used for microscopy (Fig. S41).

3.4.2. A549 with OS21

A549 cells (50.000 per well) were seeded and grown for 48 hrs in [DMEM/Hams F12 1:1 (v/v) with 10% FBS and 1% penicillin/streptomycin] on µ-slides (ibidi). The medium [DMEM/Hams F12 1:1 (v/v) with 10% FBS and 1% penicillin/streptomycin] was removed and the cells were washed with PBS (preheated at 37°C) twice. Probe OS21 (1 µM, 200 µL) was added and incubated for 30 minutes at 37°C and 5% CO_2. Cells were washed with PBS for three times. Nucleus staining was done with Hoechst 33342 (2 µg/ml, 200 µL) at 37°C and 5% CO_2 for 10 min. Cells were washed with PBS once and used for microscopy (Fig. S42).

3.5 Transfection of HUVEC cells with siRNA

Two days before transfection, 0.7×10^6 HUVEC cells were seeded in two T25 flasks in EGM (Endothelial Cell Growth Medium, from PromoCell) with 2% serum at 37°C, 5% CO_2.

For each sample, 60 µL of 10 µM siRNA solution was diluted with 240 µL OptiMEM, and 15 µL lipofectamine RNAiMAX was diluted with 285 µL OptiMEM. The prepared siRNA and

lipofectamine solutions were prepared separately and stabilized at room temperature for 10 min before gently mixing the two solutions and incubating for another 30 min at room temperature. The collected HUVEC cells were washed twice with OptiMEM and added to 2.4 mL OptiMEM in T25 flasks.The siRNA-lipofectamine complex (600 µL) was added to 2.4 mL HUVEC cells, and the mixture was incubated at 37°C, 5% CO_2 for 4 h. Then, the medium was changed to EGM, and the cells were incubated for 48h. The mock transfection was performed in the same way but with lipofectamine and OptiMEM only. To assess the transfection efficiency, untreated HUVEC cells, HUVEC cells + lipofectamine only and HUVEC cells with siRNA + lipofectamine were stained with two specific antibodies for VEGFR2 (APC anti-human CD309 Antibody from BioLegend) and $\alpha_v\beta_3$ (Alexa Fluor® 488 anti-human CD51/61 Antibody from BioLegend). The transfection efficiency was calculated by flow cytometry and comparing the signal of anti-integrin antibody (A488) from HUVEC cells transfected with siRNA + lipofectamine with that from the cells with mock transfection (Fig. S39).

3.6 Cell viability

HUVEC, A-498, HCT-116, and HEK-293 cells were seeded into wells of a 96 well-microtiter plate (100 µL in respective medium , see section 3.2.3 to 3.2.6 for the medium) at a density of 10^4 cells/well. Cells were allowed to grow for 24 h to a confluency of ca. 85%. The medium was removed and wells carefully washed once with PBS (preheated at 37°C). The probes were added at specific concentrations. The cells were incubated at 37°C, 5% CO_2 for 10 minutes. After one careful wash with PBS, 10% alamar blue was added, and the fluorescence signal (590 nm) was recorded after 6 hours.

3.7 Mix cell analysis

I). Selected Constructs:

a. LC40 (the bi-specific DNA construct conjugated with Atto-565),

b. vcMMAE-LC40 (the bi-specific DNA construct linked with vcMMAE)

c. uDNA (ligand free DNA complex LC40)

Concentration used: 200 nM, incubation time: 10 minutes, temperature: 37 °C, 5% CO_2 medium.

II). Cell Lines Under Consideration: HUVEC, eGFP-MS2BP expressing HEK293 cells (see 3.2.8), and HCT-116 cells.

III). Employed Antibodies: FITC anti-human CD326 (EpCAM) Antibody for HCT-116 cells.[1]

IV). Stains for Assessing Apoptosis and Cell Death: Sytox Red[2] and Propidium Iodide (PI) for evaluating membrane integrity; Annexin-V-FITC, and Annexin-V-Alexa Fluor 647 for detecting phosphatidylserine as marker of apoptosis.[3]

V). Differentiation between cells in cell mixtures: A combination of equivalent quantities of cells was prepared, focusing on two cell mixes: HUVEC / HEK293/eGFP-MS2BP) and HUVEC / HCT116. 100,000 cells of each in suspension is first centrifuged, followed by the addition of 100 µL PBS, then the cells are mixed. Within a mixture with HUVECs, HEK293(eGFP-MS2BP) cells were identified based on GFP signal, and thus the untreated cell mixture will exhibit two populations in flow cytometry, one GFP↓ for HUVEC cells, and the other GFP↑ for HEK293(eGFP-MS2BP) cells. In HUVEC/HCT116 mixtures, HCT-116 cells were tagged with the anti-EpCAM$_{FITC}$ antibody. Flow cytometry showed a population with FITC↑ for HCT-116 cells and FITC↓ for the HUVECs.

VI). Staining of mixed cell populations with Atto565-LC40: 100,000 cells were centrifuged individually first and then mixed together in PBS. Then, the mixed cell populations were treated with unlabelled, ligand-free DNA, i.e. LC40 lacking ligands and label (200 nM, 10 minutes, 100 µL PBS, 37°C, 5% CO_2). Subsequently, the cells were centrifuged and washed with 100 µL PBS (1x, 37°C). Then the cells were treated with LC40 (200 nM, 10 minutes, 100 µL PBS, 37°C, 5% CO_2). After treatment, the cells were centrifuged and washed with 100 µL PBS (2x, 37°C) and analysed by flow cytometry in 400 µL PBS (Fig. 5).

VI). Treatment of mixed cell populations with vcMMAE-LC40: 100,000 cells were centrifuged individually first and then mixed together in PBS. Then, the mixed cell populations were treated with unlabelled, ligand-free DNA, i.e. LC40 lacking ligands and label (200 nM, 10 minutes, 100 µL PBS, 37°C, 5% CO_2). Subsequently, the cells were centrifuged and washed with 100 µL PBS (1x, 37°C). Then the cells were treated with vcMMAE-LC40 (200 nM, 10

[1] Regarding EpCAM for HCT-116 cells, detailed information can be obtained: (a) Roshan et al, Molecular Biotechnology (2023) 65:637–644, (b) Cohan et al, Molecular Immunology 129 (2021) 70–77, (c) Lee et al. Journal of Translational Medicine (2023) 21:530 and (d) https://www.thermofisher.com/antibody/product/CD326-EpCAM-Antibody-clone-G8-8-Monoclonal/11-5791-82

[2] https://www.thermofisher.com/de/de/home/references/protocols/cell-and-tissue-analysis/protocols/sytox-dead-cell-stains-protocol.html

[3] https://www.biolegend.com/ja-jp/products/alexa-fluor-647-annexin-v-5276?GroupID=BLG6046

minutes, 100 µL PBS, 37°C, 5% CO_2). After treatment, the cells were centrifuged and washed with 100 µL PBS (2x, 37°C) and kept in antibiotic-free full medium at 37°C.

After four hours, the cells were centrifuged and washed with 100 µL 1X Annexin V binding buffer (kept at RT) twice, and kept in suspension for the next 5 minutes. Meanwhile, 5µL Annexin-V_{AF647} (as obtained from the vendor) was taken in 100 µL binding buffer (kept at RT) and added to the cell mixture, followed by 15 minutes incubation at RT. Then, the cells were centrifuged and washed once with the 100 µL binding buffer at RT. In case of staining with both Annexin V-FITC and PI, 5 µL Propidium Iodide Staining Solution (as obtained from the vendor) was added to binding buffer (100 µL) Flow cytometry was performed in 400 µL binding buffer.

Regarding untreated cells, the cells were kept for four hours in suspension, following the same procedure as mentioned above.

In experiments involving exclusive Sytox Red staining, treated and non-treated cell populations kept in antibiotic-free medium for 4 h at 37°C were centrifuged and stained with 5 nM Sytox Red in PBS, followed by 10 minutes incubation at RT. After 10 minutes, cells were measured using flow cytometry in 400 µL PBS.

Regarding Annexin V-FITC and PI treatment, 10X Binding Buffer: 0.1 M HEPES, pH 7.4; 1.4 M NaCl; 25 mM $CaCl_2$. Dilute to 1X prior to use. Prepare 1X binding buffer by mixing 1 part of 10X binding buffer with 9 parts of milliQ sterilized water and kept at RT for further use. Buffer was filtered using 0.1µm and 0.2µm filters two times before use.

4. UPLC Traces and Mass Spectra

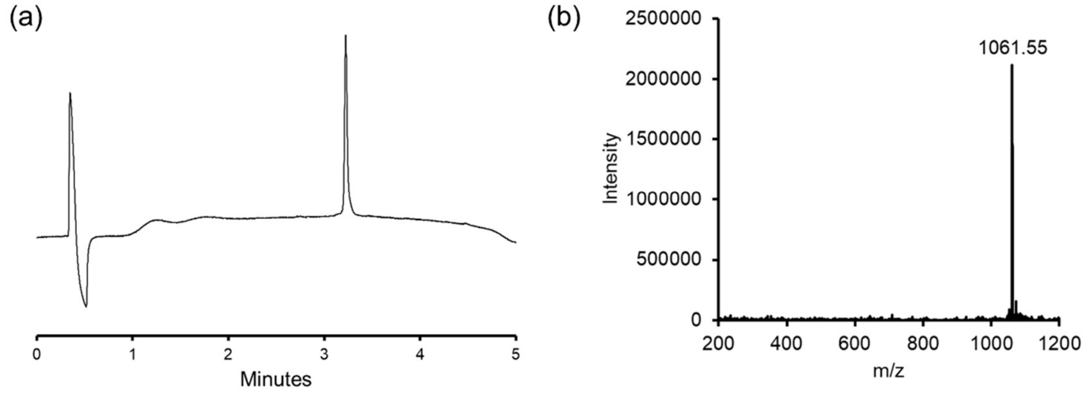

Fig. S13 (a) UPLC trace of cyclic L1 at λ = 210 nm (3-80% solvent B in 6 min); (b) calculated mass: 2122.38 [(+1); $M+H^+$], 1061.69 [(+2); $(M+2H^+)/2$]; found: 1061.55.

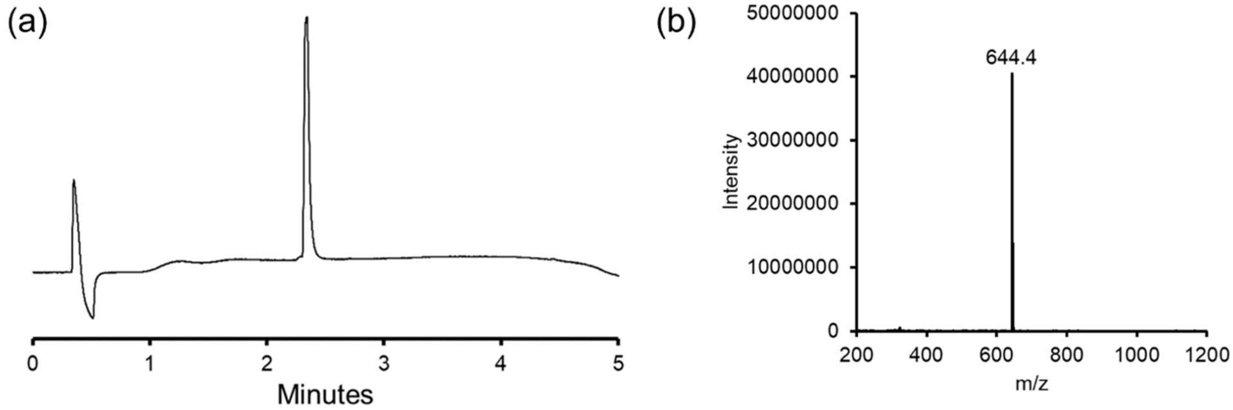

Fig. S14 (a) UPLC trace of cyclic RGD at λ = 210 nm (3-80% solvent B in 6 min); (b) calculated mass: 644.33 [(+1); $M+H^+$]; found: 644.4.

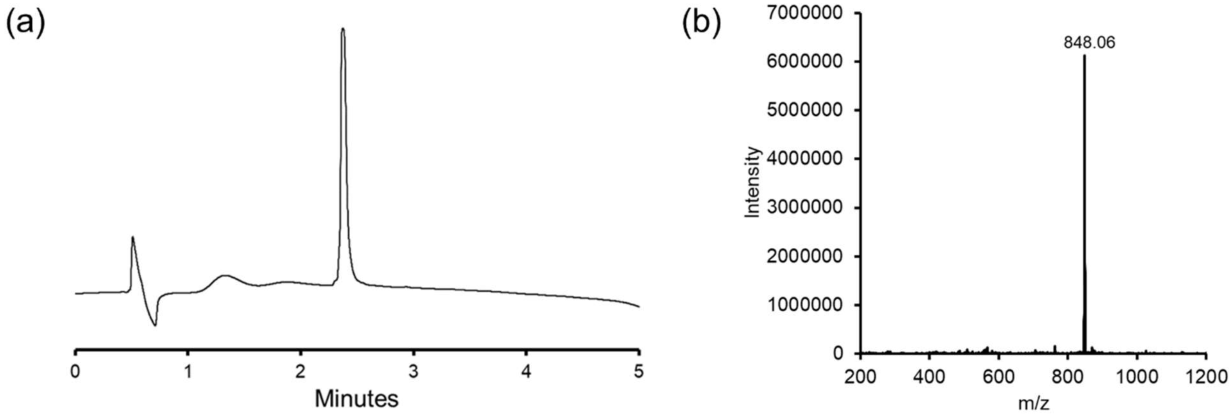

Fig. S15 (a) UPLC trace of GE11 (S) at λ = 210 nm (3-80% solvent B in 6 min); (b) calculated mass: 1694.89 [(+1); $M+H^+$], 847.95 [(+2); $(M+2H^+)/2$]; found: 848.06.

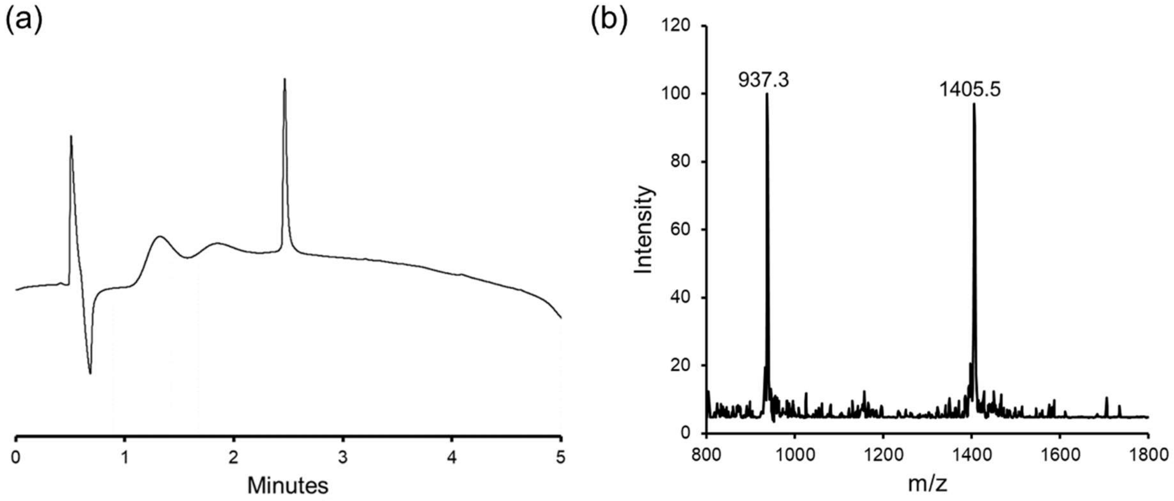

Fig. S16 (a) UPLC trace of GE137 (O) at $\lambda = 210$ nm (3-80% solvent B in 6 min); (b) calculated mass with two S-S bonds 2810.09 [(+1); $M+H^+$], 1405.55[(+2); $(M+2H^+)/2$], 937.37 [(+3); $(M+3H^+)/3$]; found: 937.3 and 1405.5.

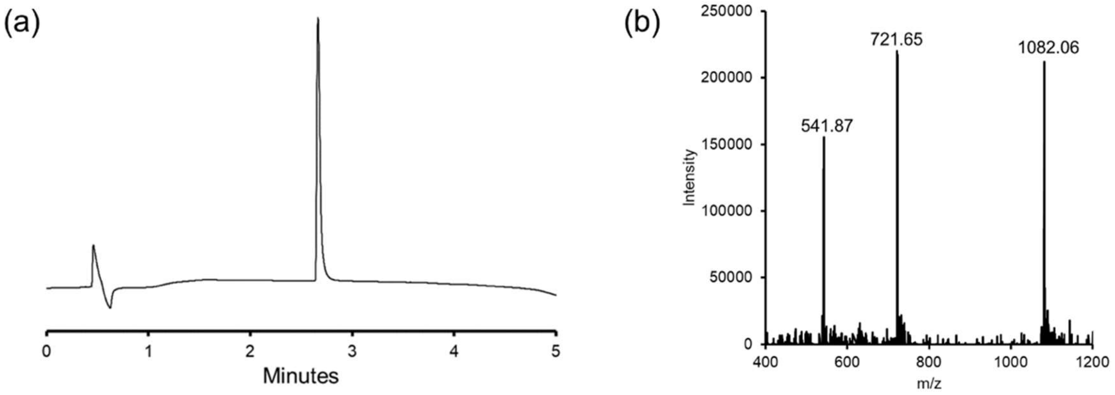

Fig. S17 (a) UPLC trace of GE11TAMRA at $\lambda = 210$ nm (3-80% solvent B in 6 min); (b) calculated mass 1082.21[(+2); $(M+2H^+)/2$], 721.80 [(+3); $(M+3H^+)/3$], 541.60 [(+4); $(M+4H^+)/4$]; found: 1082.06, 721.65, 541.87.

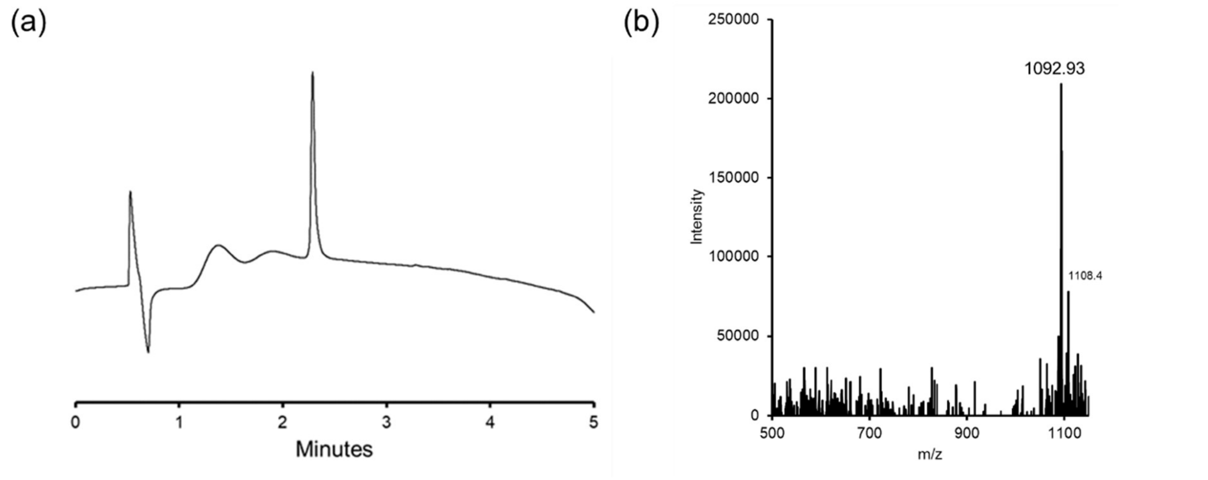

Fig. S18 (a) UPLC trace of GE137TAMRA at λ = 210 nm (3-80% solvent B in 6 min); (b) calculated mass 1093.03 [(+3); (M+3H$^+$)/3]; found: 1092.93.

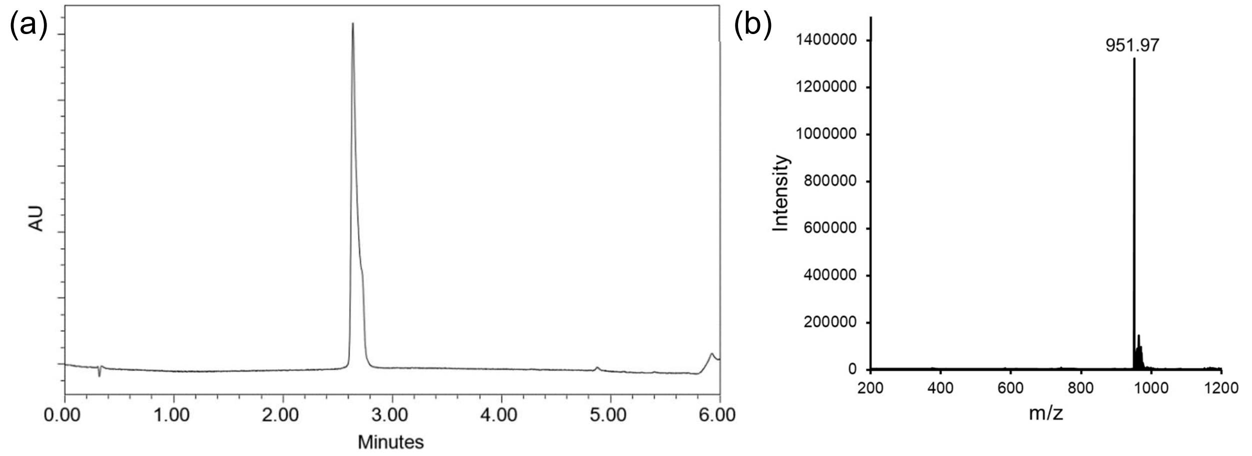

Fig. S19 (a) UPLC trace of AF568-Cyclic L1 at λ = 280 nm (3-80% solvent B in 6 min); (b) calculated mass 951.72 [(+3); (M+3H$^+$)/3]; found: 951.97.

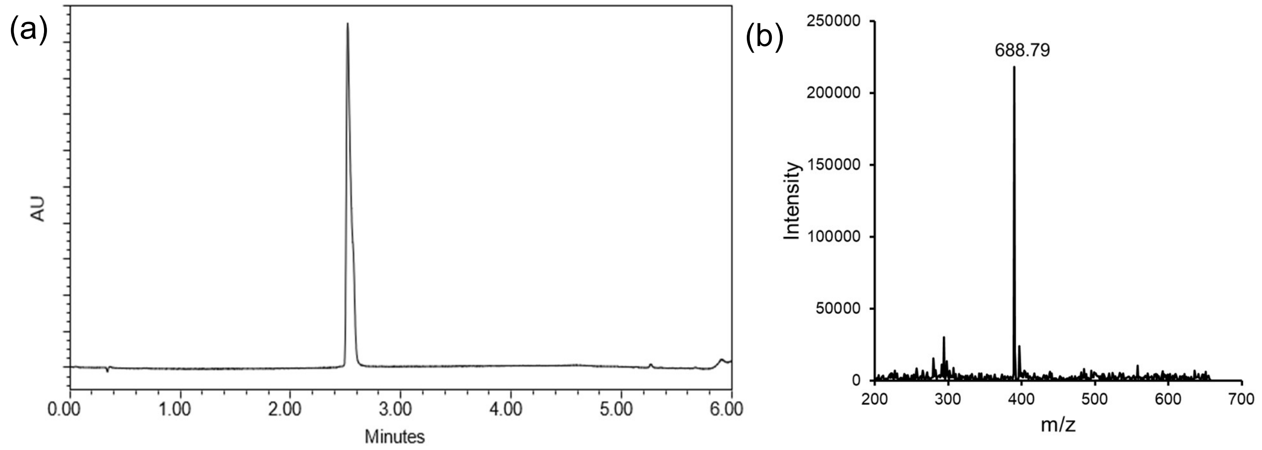

Fig. S20 (a) UPLC trace of AF568-Cyclic RGD at λ = 280 nm (3-80% solvent B in 6 min); (b) calculated mass 688.27 [(+2); (M+2H$^+$)/2]; found: 688.79.

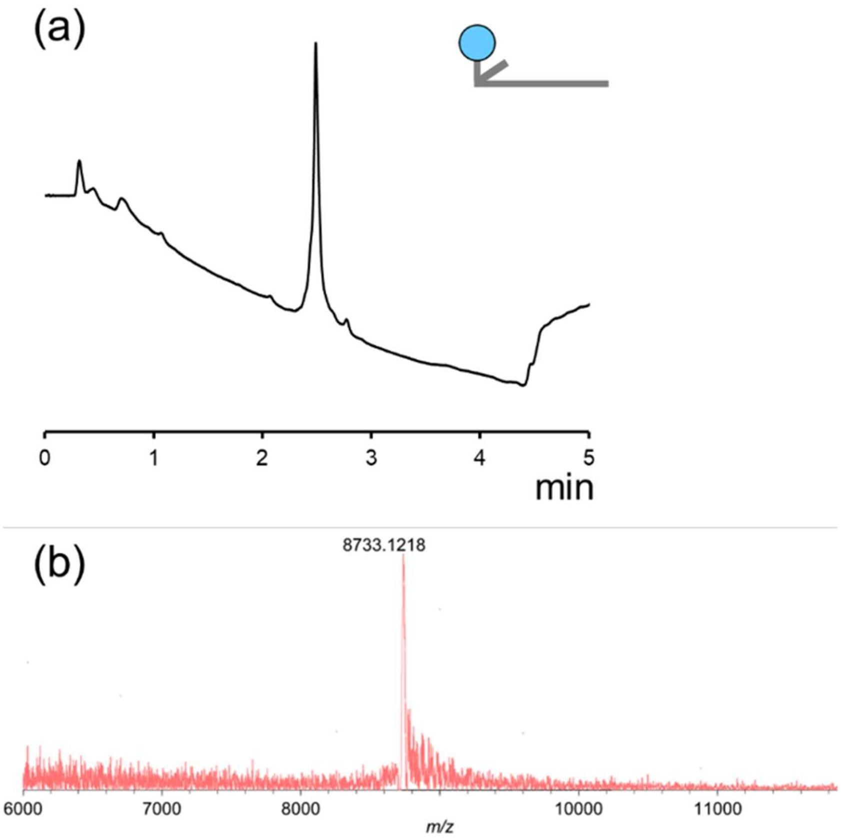

Fig. S21 UPLC traces at λ = 260 nm (t_R: 2.49 min, 3-30% solvent B in 6 min) of (a) 3′$_1$L and (b) calculated mass: 8732, found: 8733.12 in MALDI.

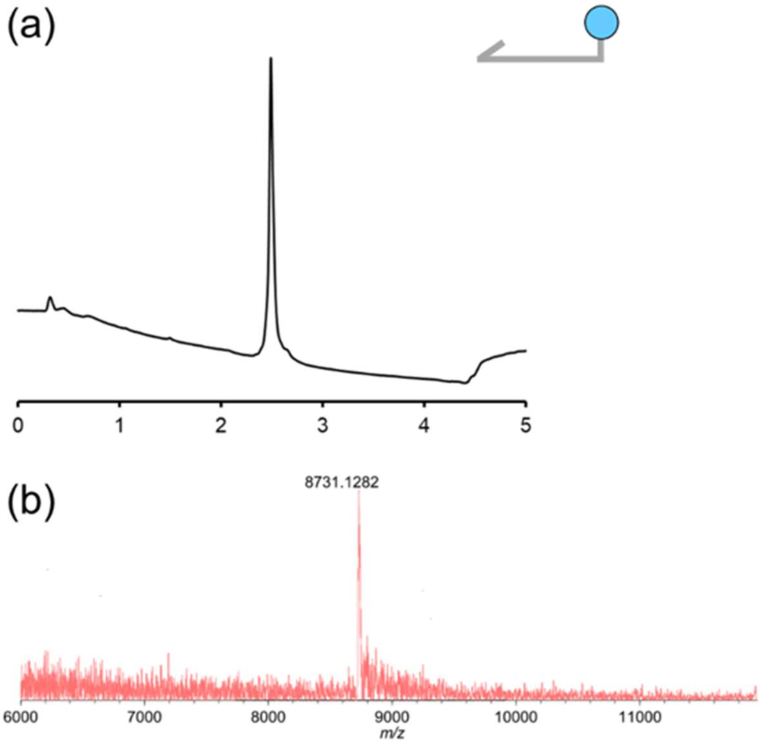

Fig. S22 UPLC traces at λ = 260 nm (t_R: 2.49 min, 3-30% solvent B in 6 min) of (a) 5′$_1$L DNA and (b) calculated mass: 8732, found: 8731.12 in MALDI.

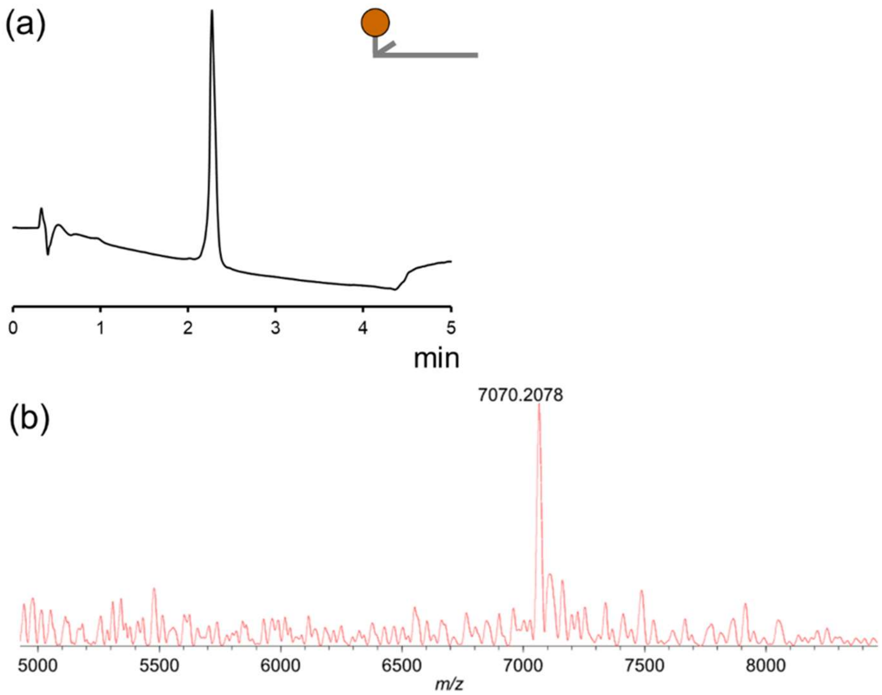

Fig. S23 UPLC traces at λ = 260 nm (t_R: 2.27 min, 3-30% solvent B in 6 min) of (a) 3′$_2$C and (b) calculated mass: 7070, found: 7070.20 in MALDI.

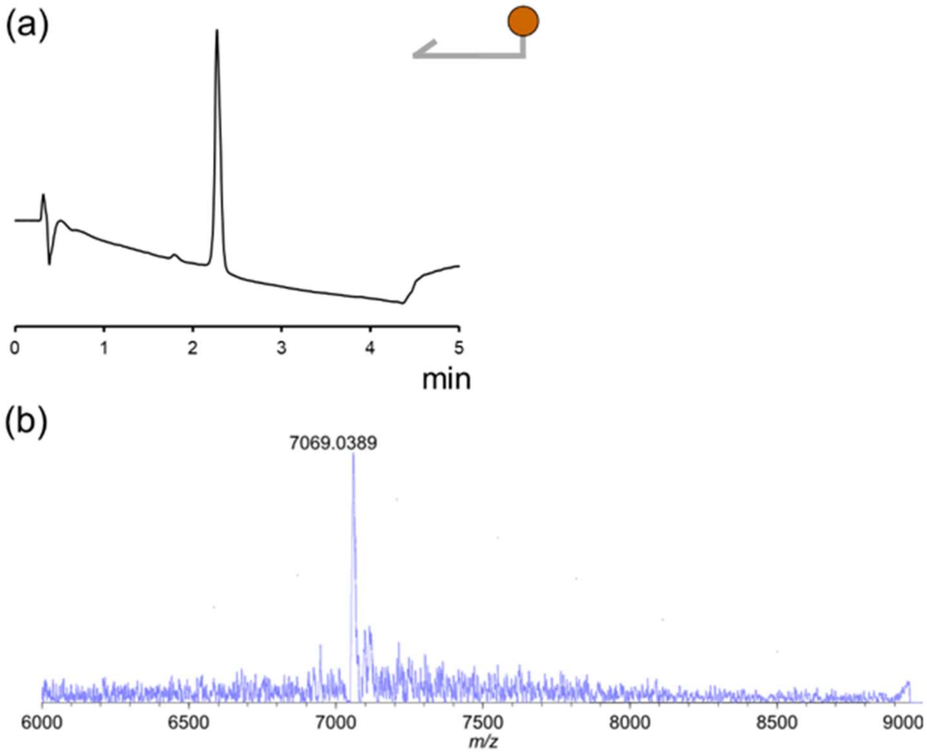

Fig. S24 UPLC traces at λ = 260 nm (t_R: 2.27 min, 3-30% solvent B in 6 min) of (a) 5′$_2$C DNA(b) calculated mass: 7070, found: 7069.03 in MALDI.

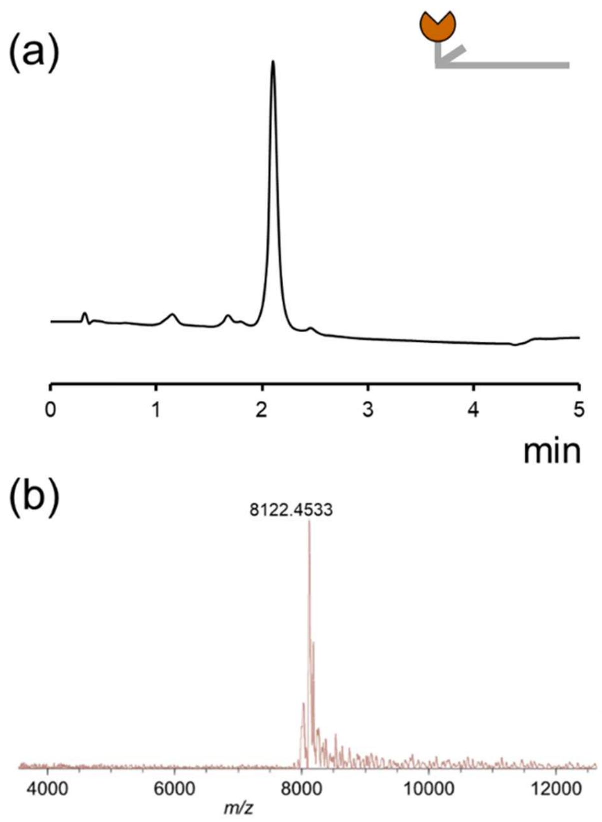

Fig. S25 UPLC traces at λ = 260 nm (t_R: 2.10 min, 3-30% solvent B in 6 min) of (a) 3'$_2$ S and (b) calculated mass: 8121, found: 8122.45 in MALDI.

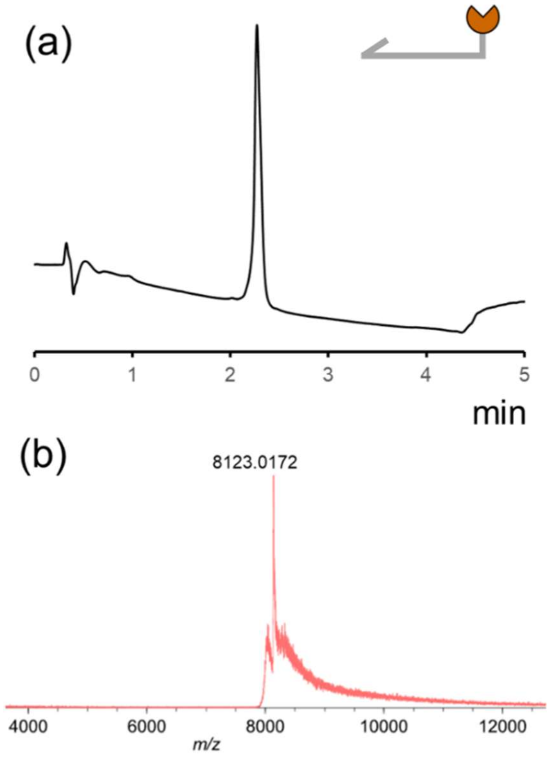

Fig. S26 UPLC traces at λ = 260 nm (t_R: 2.27 min, 3-30% solvent B in 6 min) of (a) 5'$_2$ S and (b) calculated mass: 8121, found: 8123.01 in MALDI.

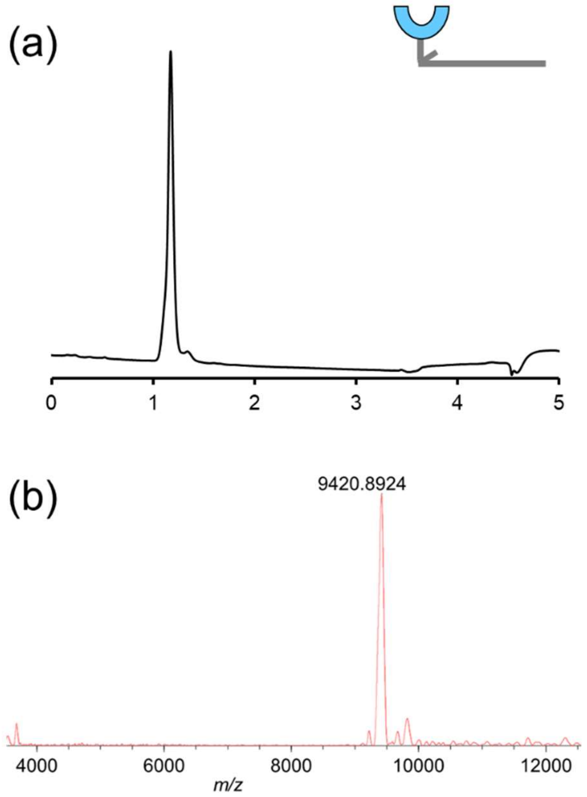

Fig. S27 UPLC traces at λ = 260 nm(t_R: 1.17 min, 3-30% solvent B in 6 min) of (a) 3'$_1$ O and (b) calculated mass: 9420.09, found: 9420.89 in MALDI.

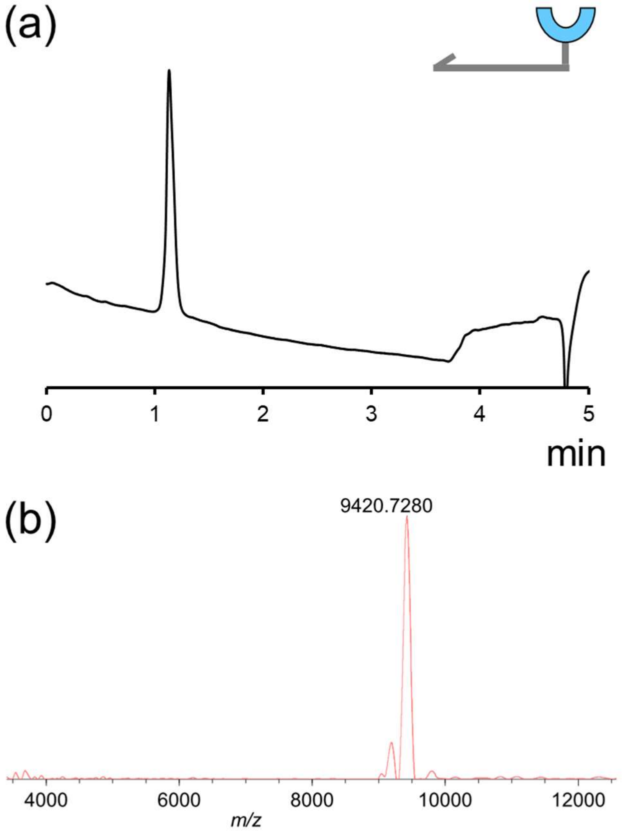

Fig. S28 UPLC traces at λ = 260 nm (t_R: 1.13 min, 3-30% solvent B in 6 min) of (a) 5'$_1$ O and (b) calculated mass: 9420.09, found: 9420.72 in MALDI.

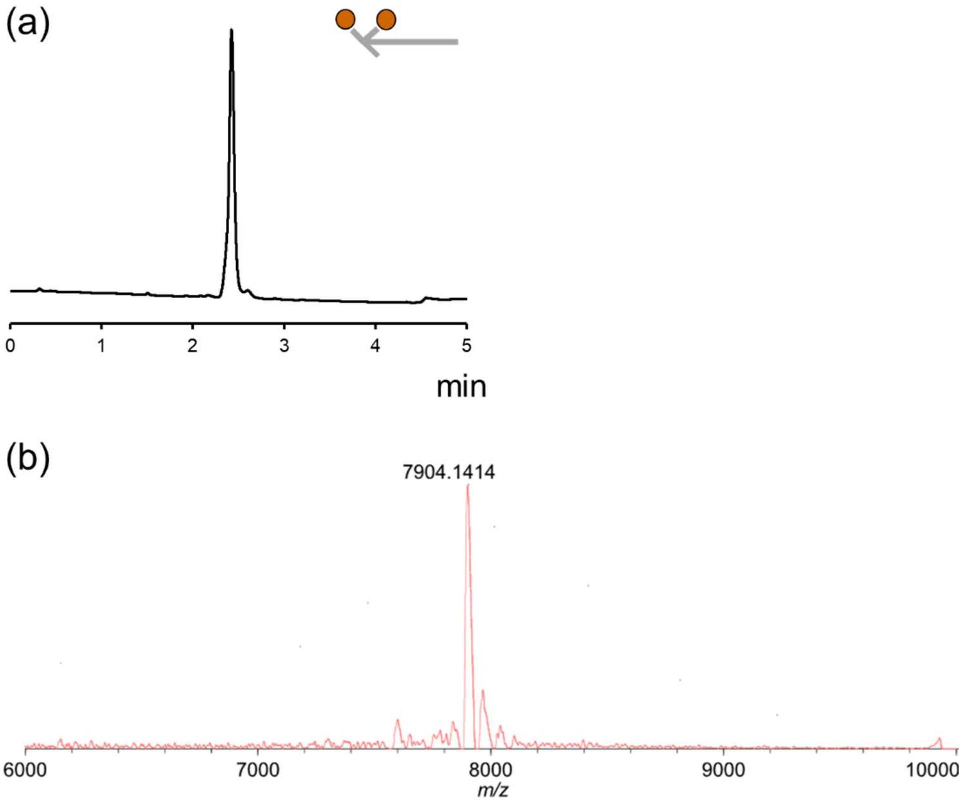

Fig. S29 UPLC traces at λ = 260 nm (t_R: 2.43 min, 3-30% solvent B in 6 min) of (a) 3'$_2$ C2 and (b) calculated mass: 7904.8, found: 7904.14 in MALDI.

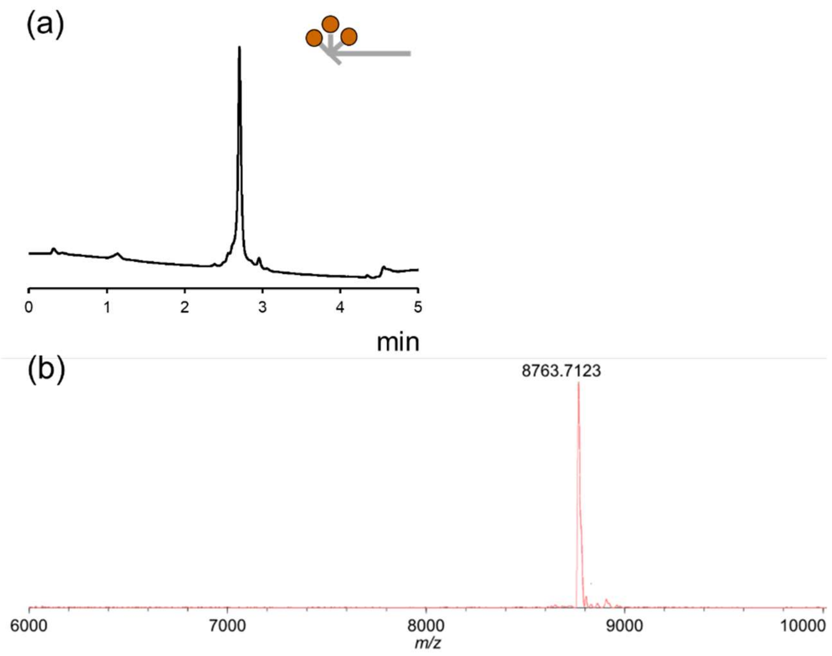

Fig. S30 UPLC traces at λ = 260 nm (t_R: 2.70 min, 3-30% solvent B in 6 min) of (a) 3'$_2$ C3 and (b) calculated mass: 8758.2, found: 8763.71 in MALDI.

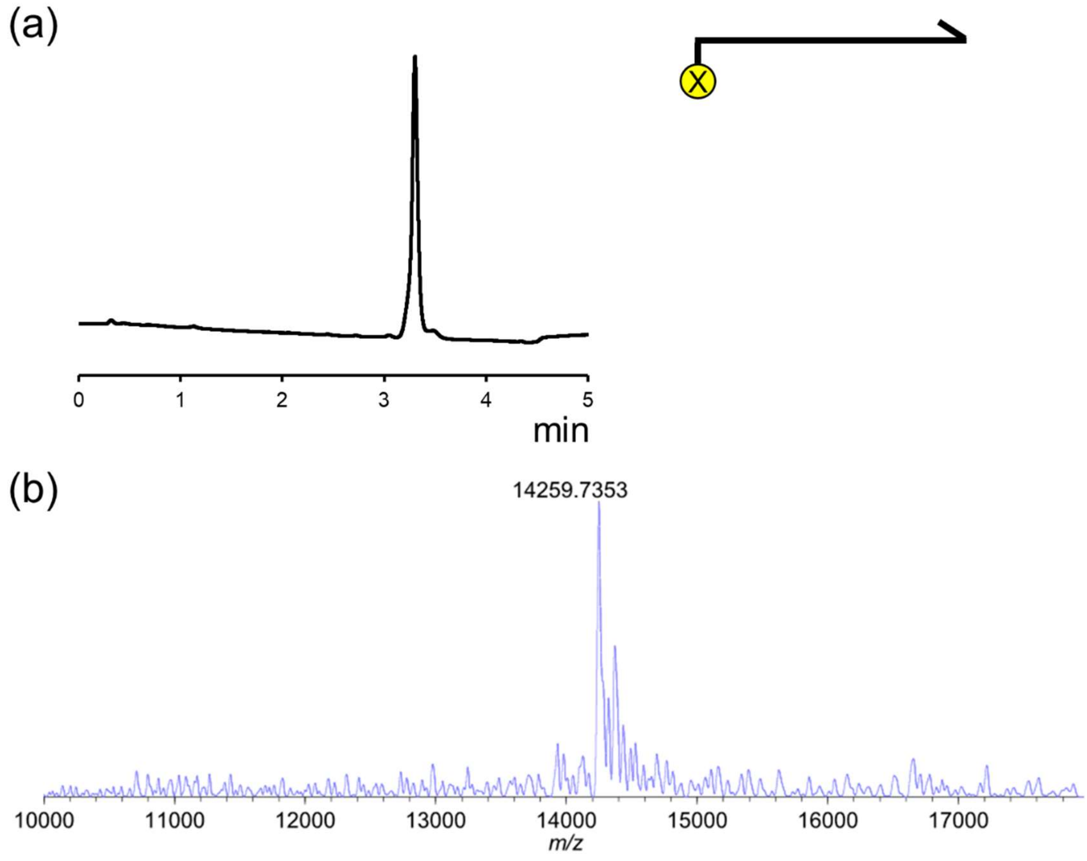

Fig. S31 (a) UPLC traces at λ = 260 nm (t_R: 3.30 min, 3-30% solvent B in 6 min) of Template strand (41mer) with MMAE; (b) Calculated mass: 14259.6, found: 14259.73 in MALDI.

5. Gating Strategy in Flow cytometry

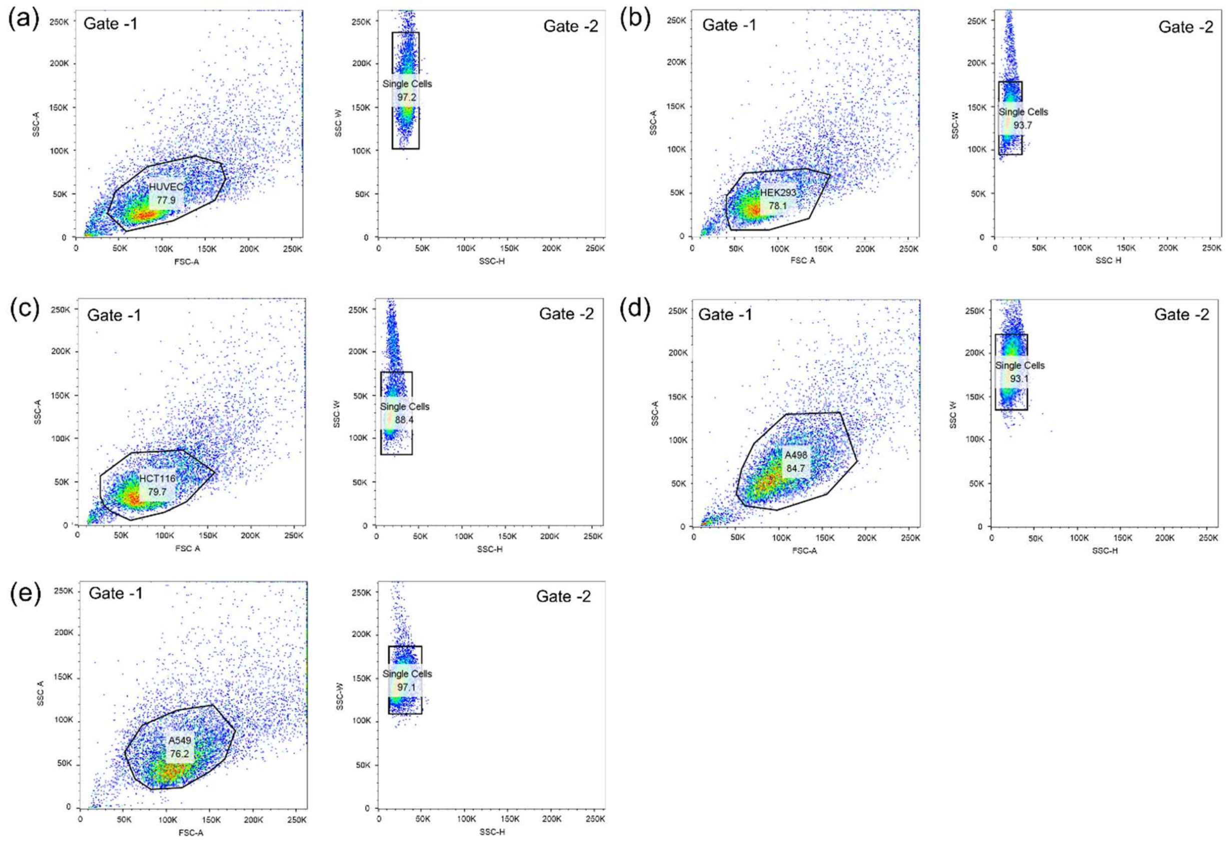

Fig. S32 Exemplified gating strategy for a) HUVECs, b) HEK293 cells, c) HCT116 cells, d) A496 cells and e) A549 cells applied in flow cytometry analysis excluding cells debris (FSC-A, SSC-A; Gate 1) and doublets (SSC-H, SSC-W; Gate 2). Approx. 10,000 cells per sample were analysed. Channels used are: Atto-565 fluorescence intensity for experiments with DNA-peptides or only peptide-TAMRA probes, and FITC channel for antibody experiment.

6. Binding of DNA-free peptides to HUVEC and A549 cells

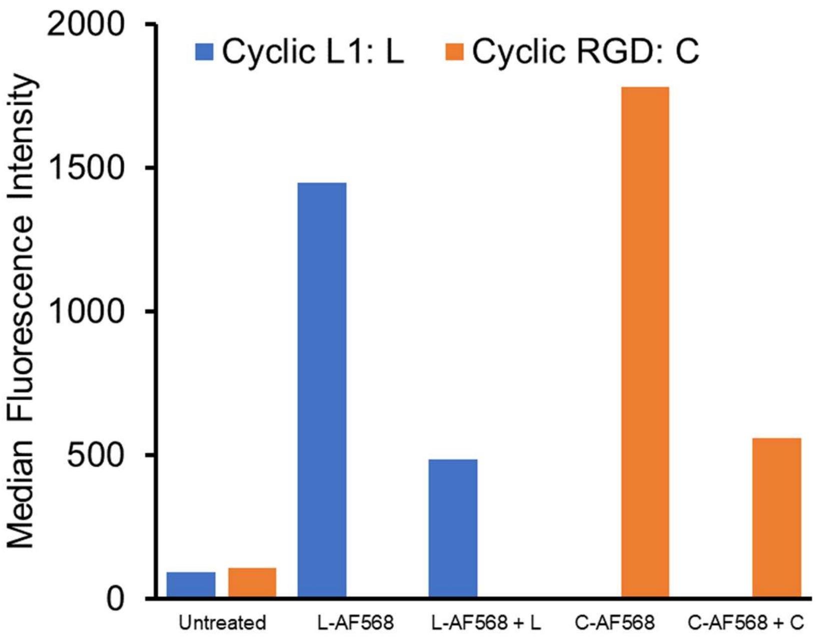

Fig. S33 Testing the specificity of binding of DNA-free peptide ligands labelled with AF-568 to HUVEC cells. incubation time: 10 minutes, temp: RT medium: HBSS.BB, flow cytometry was performed with suspended cells, 10000 cells were counted in each case and the median value was plotted. (See section 3.3.1 for detailed protocol).

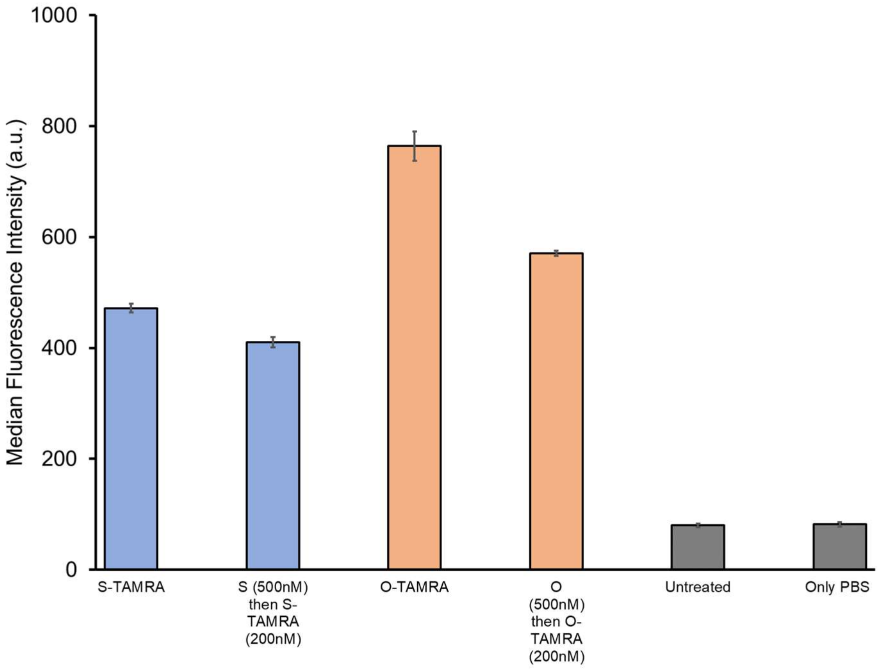

Fig. S34 Binding of TAMRA-labelled DNA-free peptides GE11 (**S**) and GE137 (**O**) to A549 cells. Incubation time: 10 minutes in PBS, temp: 37 °C, 5 % CO_2, flow cytometry was performed with suspended cells, 10000 cells were counted in each case and the median value was plotted. (See section 3.3.1 for detailed protocol).

7. Native PAGE for dsDNA

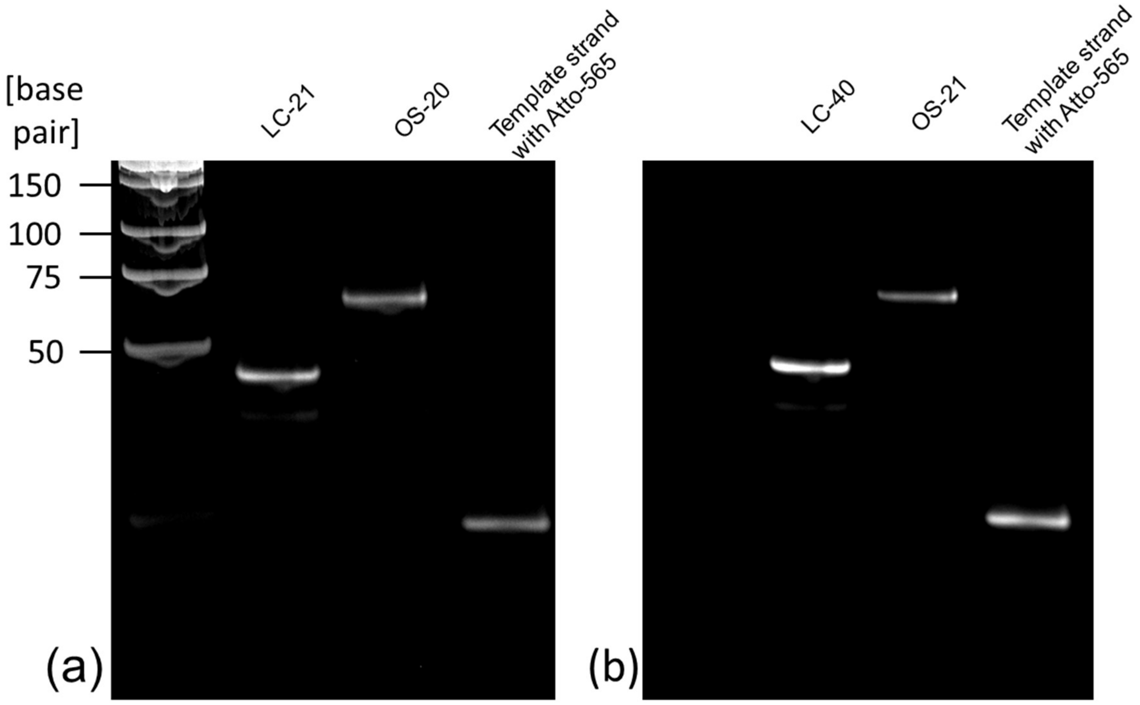

Fig. S35 Native PAGE for the characterizing duplex formation of the bispecific DNA probes visualized by (a) SYBR gold staining and (b) in-gel fluorescence of Atto565. Both channels of SYBR gold and in gel fluorescence showed single band for the dsDNA (see section: 2.9.4 for detailed protocol).

8. Analyzing the cell surface receptors using antibodies for VEGFR2 and $\alpha_V\beta_3$ integrin

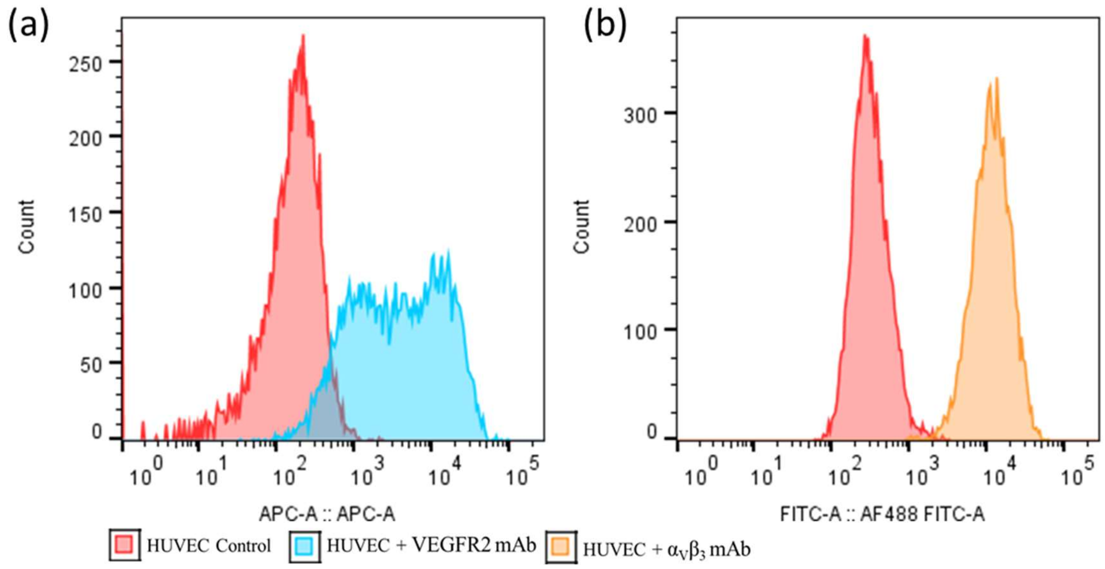

Fig. S36 Analyzing the cell surface receptors for HUVEC cells with antibody for (a) VEGFR2 and (b) $\alpha_V\beta_3$ integrin. Flow cytometry was performed with suspended cells, 10000 cells were counted in each case.

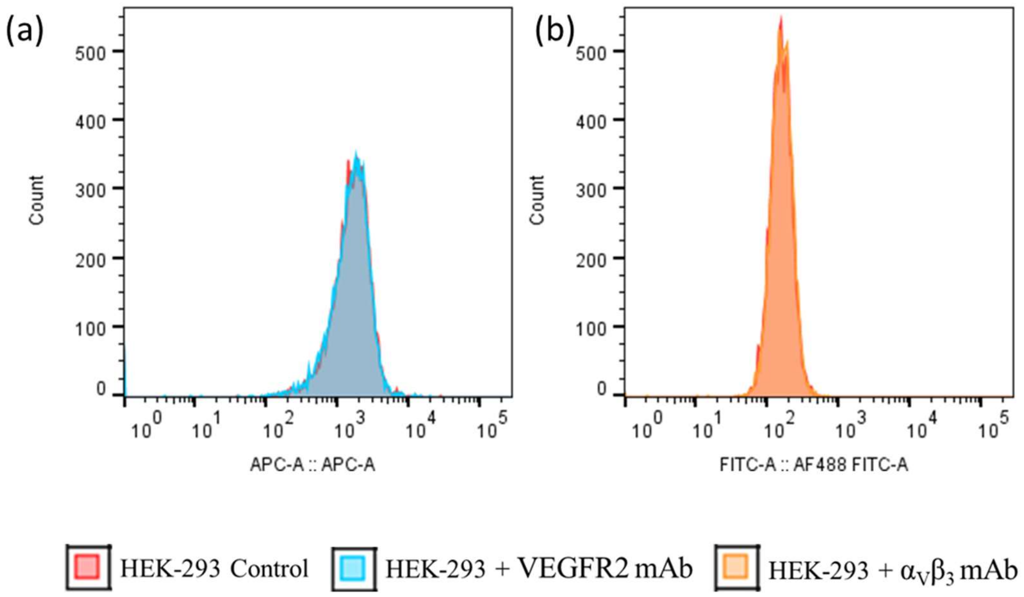

Fig. S37 Analyzing the cell surface receptors for HEK-293 cells with antibody for (a) VEGFR2 and (b) $\alpha_V\beta_3$ integrin. Flow cytometry was performed with suspended cells, 10000 cells were counted in each case.

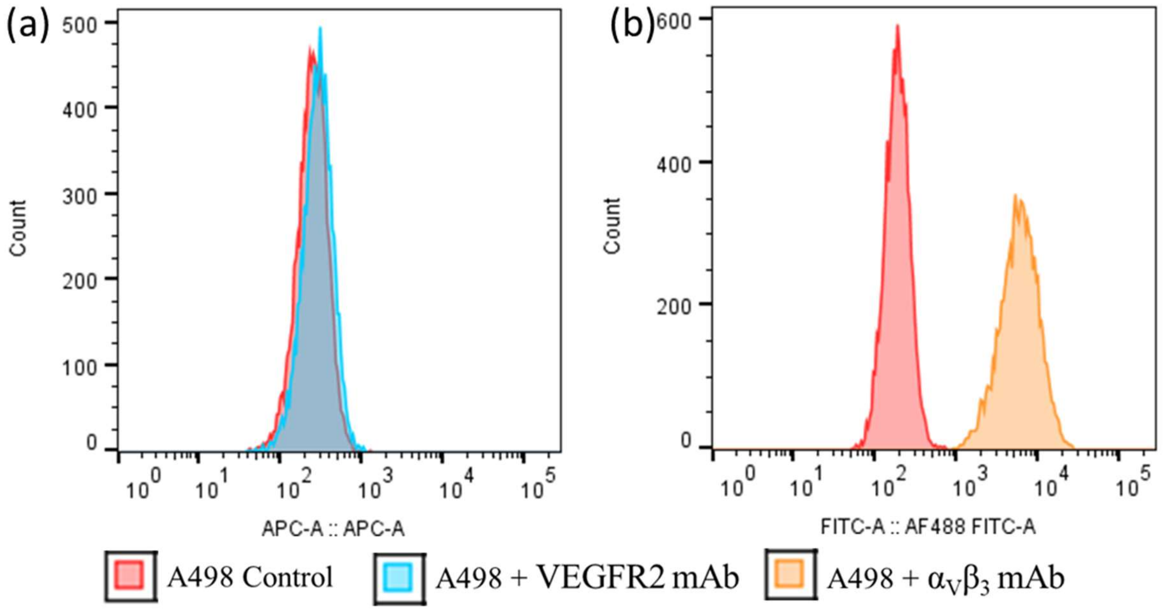

Fig. S38 Analyzing the cell surface receptors for A498 cells with antibody for (a) VEGFR2 and (b) $\alpha_V\beta_3$ integrin. Flow cytometry was performed with suspended cells, 10000 cells were counted in each case.

9. siRNA knock down

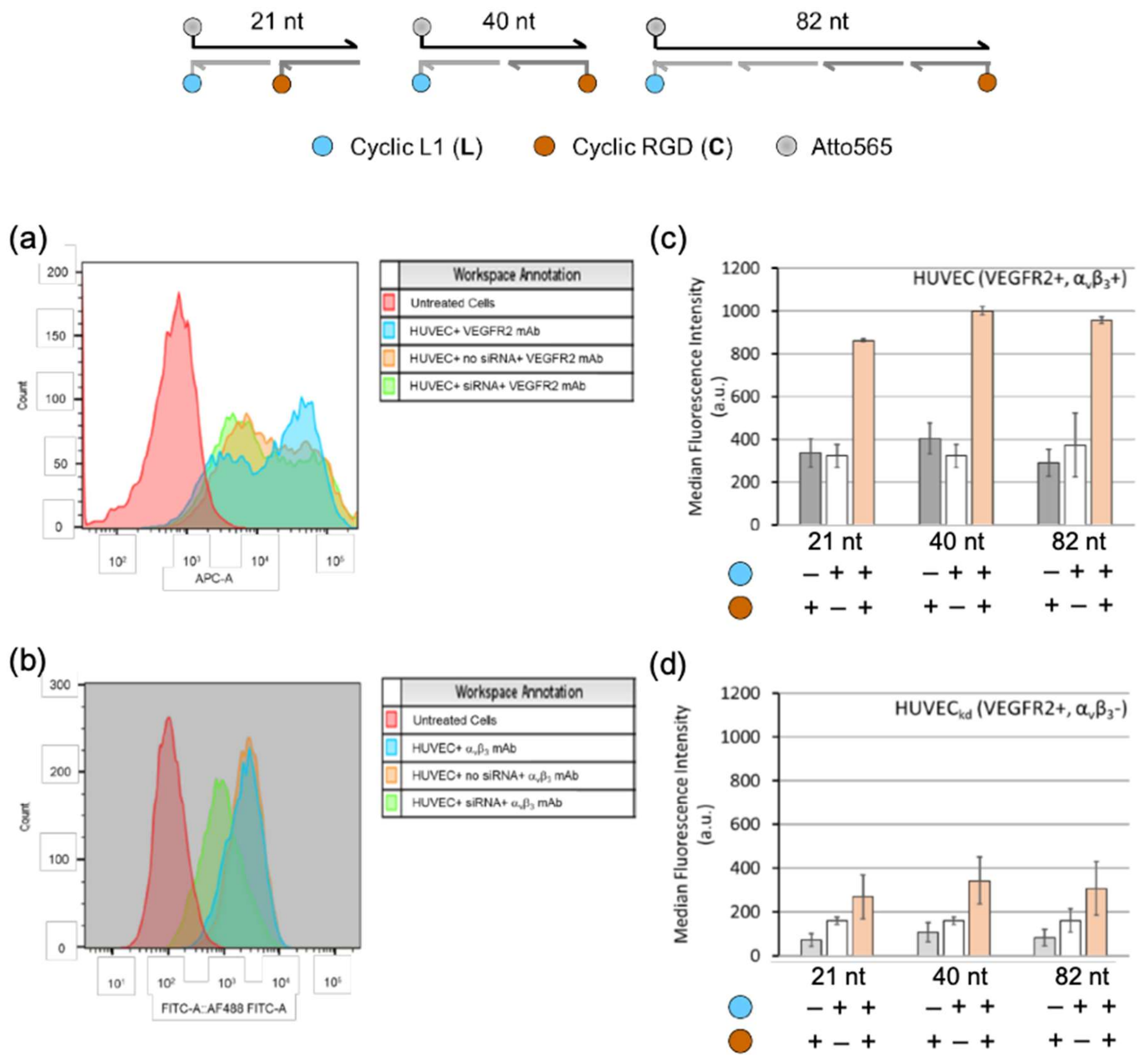

Fig. S39 Integrin β_3/ITGB3/CD61 siRNA knockdown in HUVEC cells. To check for the efficiency of the transfection, HUVEC cells were stained with (a) APC anti-human CD309 (VEGFR2) Antibody and (b) Alexa Fluor® 488 anti-human CD51/61 Antibody (targeting $\alpha_v\beta_3$). The transfection efficiency was calculated by the signal of anti-integrin antibody (A488) from HUVEC cells transfected with siRNA + lipofectamine over that from the cells with mock transfection (HUVEC + no siRNA) (that are 31%, 34%, and 47% for 3 replicates). siRNA-induced down-regulation of β3 not only reduces expression of $\alpha_v\beta_3$ integrin (see (b)) but also of VEGFR2 (see (a)). This might be due to the lack of cross-activation between VEGFR2 and $\alpha_v\beta_3$ during cell growth.

Staining of (c) mock-transfected HUVECs and (d) siRNA-transfected (β3/ITGB3/CD61 siRNA from Santa Cruz (sc-29375)) HUVECs with monovalent and bispecific DNA-peptide complexes at 4 °C assessed by flow cytometry. (See section 3.5 for detailed protocol).

10. Multiple cRGD on the DNA scaffold: Multivalency

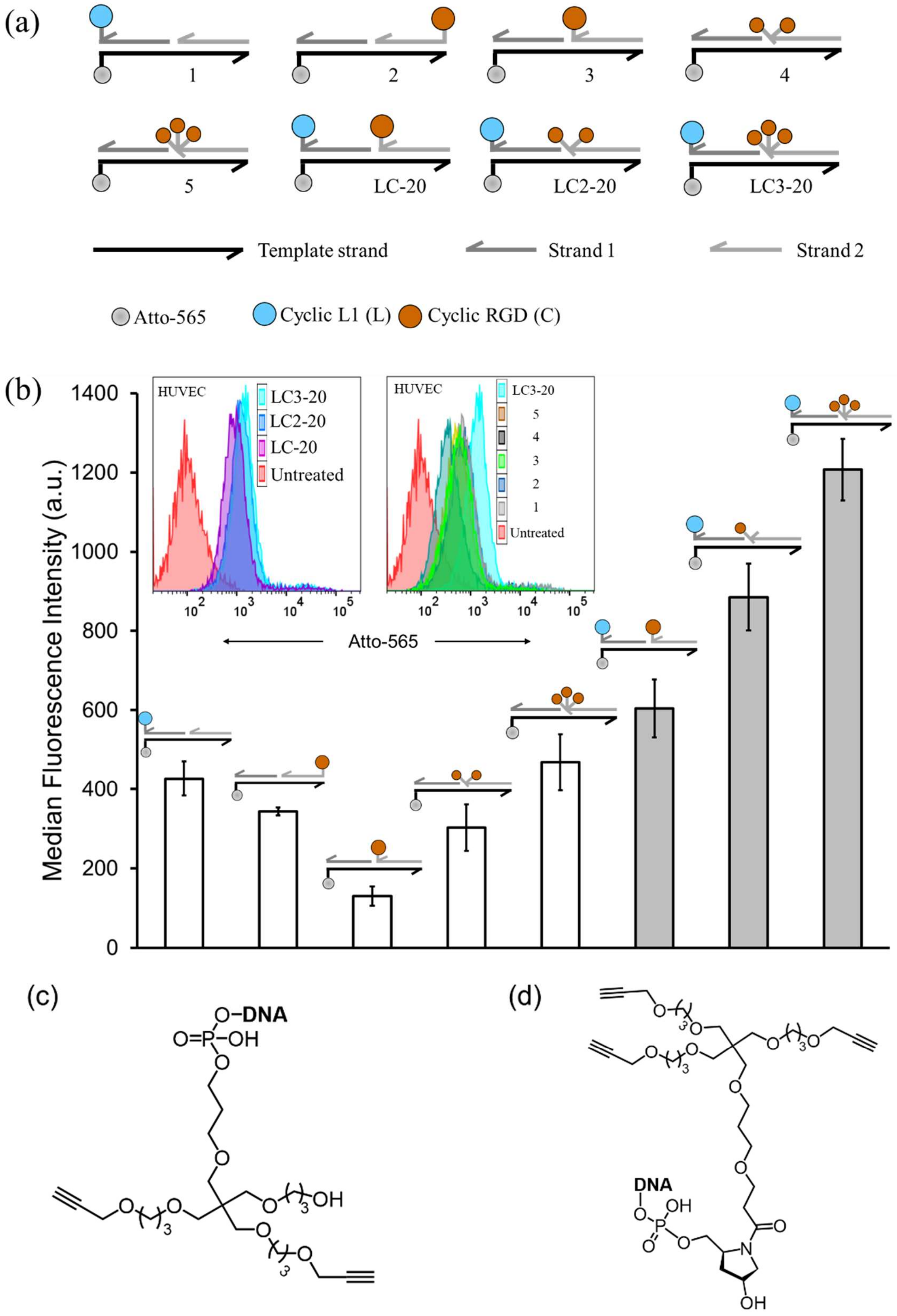

Fig. S40 Influence of multiple RGD within the strands of DNA; (a) cartoon representation of the multiple scaffolds; (b) plot of the median fluorescence intensity; inset shows representative flow cytometry images of the bi-specific and mono-specific probes, (conc$^{\underline{n}}$: 200 nM, incubation

time: 10 minutes at 37°C, 5 % CO_2 in PBS). Inset shows the flow cytometry data. Flow cytometry was performed with suspended cells, 10000 cells were counted in each case and the median value was plotted; structure of the alkyne (c) doubler and (d) trebler linker on 3´ position connected with DNA.

11. Fluorescence Microscopy

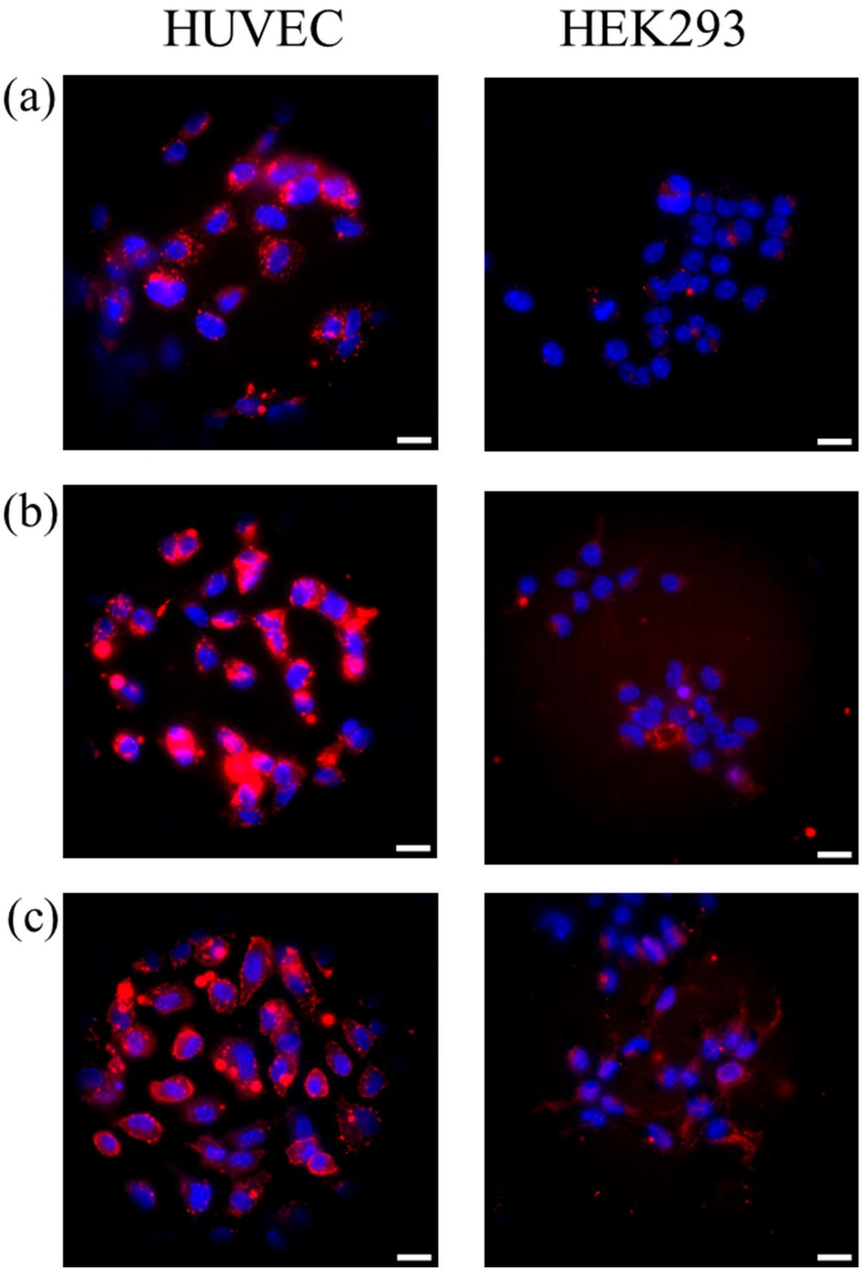

Fig. S41 Microscopy images of cells stained with 500 nM bispecific probes (a) LC21; (b) LC40 and (c) LC82, incubation time: 1 h, 4 °C, 5 % CO_2. Blue: nuclear labelling with Hoechst33342,

($\lambda ex = 350 \pm 50$ nm, $\lambda em = 460 \pm 50$ nm); Red: Signal from Atto-565 in TRITC channel ($\lambda ex = 575 \pm 25$ nm, $\lambda em > 593$ nm). Scale bar is 20 µm.

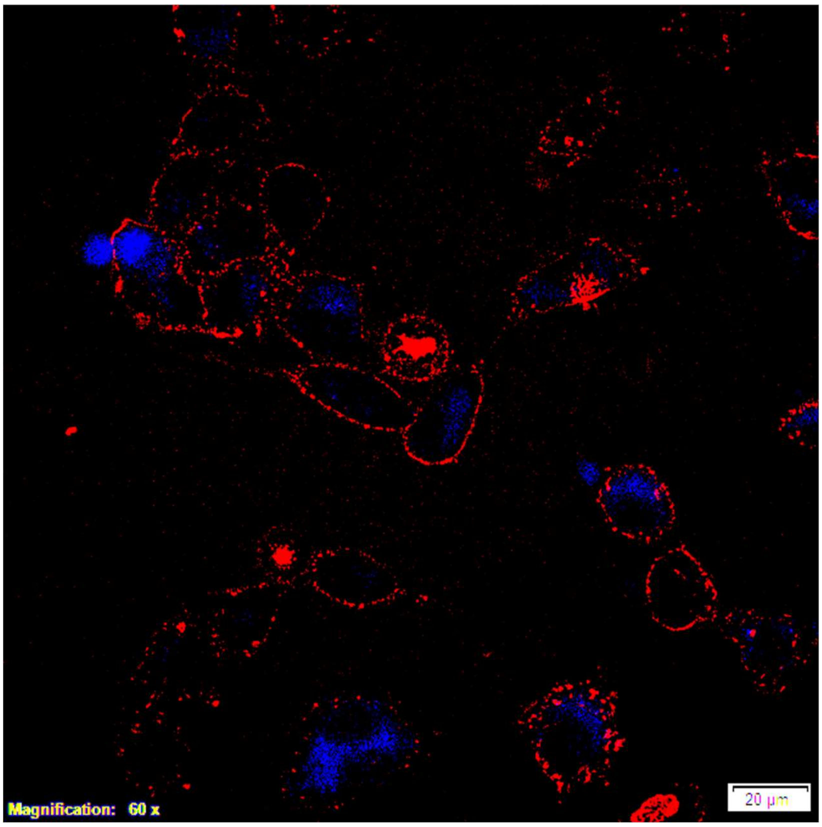

Fig. S42 Microscopy images of A549 cells after staining with OS21. Conditions: 1 µM OS21 in PBS, 30 minutes incubation time, 37 °C, 5 % CO_2. scale bar: 20 µm; medium: PBS, 50.000 cells seeded per well. Blue: nuclear labelling with Hoechst33342 ($\lambda ex = 350 \pm 50$ nm, $\lambda em = 460 \pm 50$ nm); Red: Signal of Atto-565 in TRITC channel ($\lambda ex = 575 \pm 25$ nm, $\lambda em > 593$ nm).

12. Concentration dependent flow cytometry

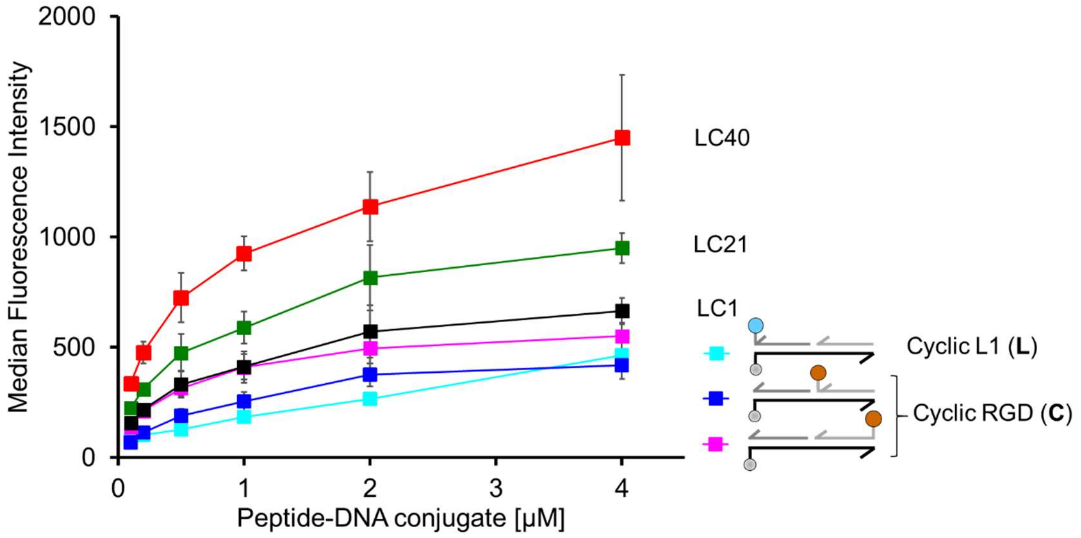

Fig. S43 Concentration dependent binding of monovalent and bispecific (LC1, LC21, LC40) peptide-DNA complexes to HUVECs assessed by flow cytometry. Conditions: 10 min incubation, 37 °C, 5 % CO_2, PBS.

13. Cytotoxicity of non-conjugated MMAE

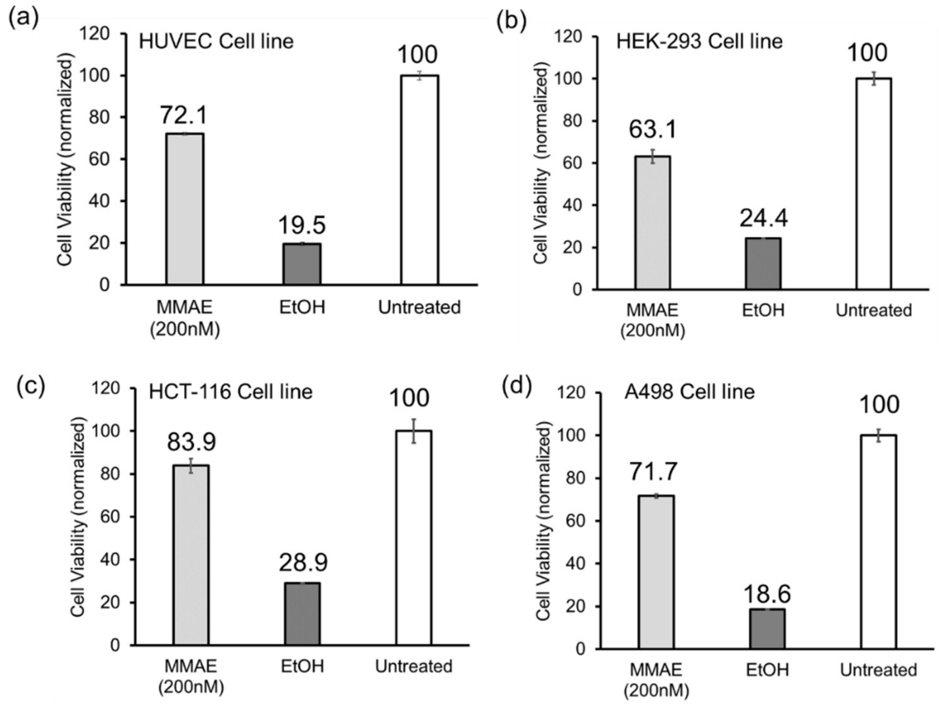

Fig. S44 Cytotoxic effect of MMAE on HUVEC, HEK-293, HCT-116 and A498 cells, Conditions: 10 min incubation in PBS at 37 °C, 5 % CO_2, afterwards, 10% Alamar blue^{TM} was used in respective full medium for 6 hrs.

14. Characterization of Cell Death

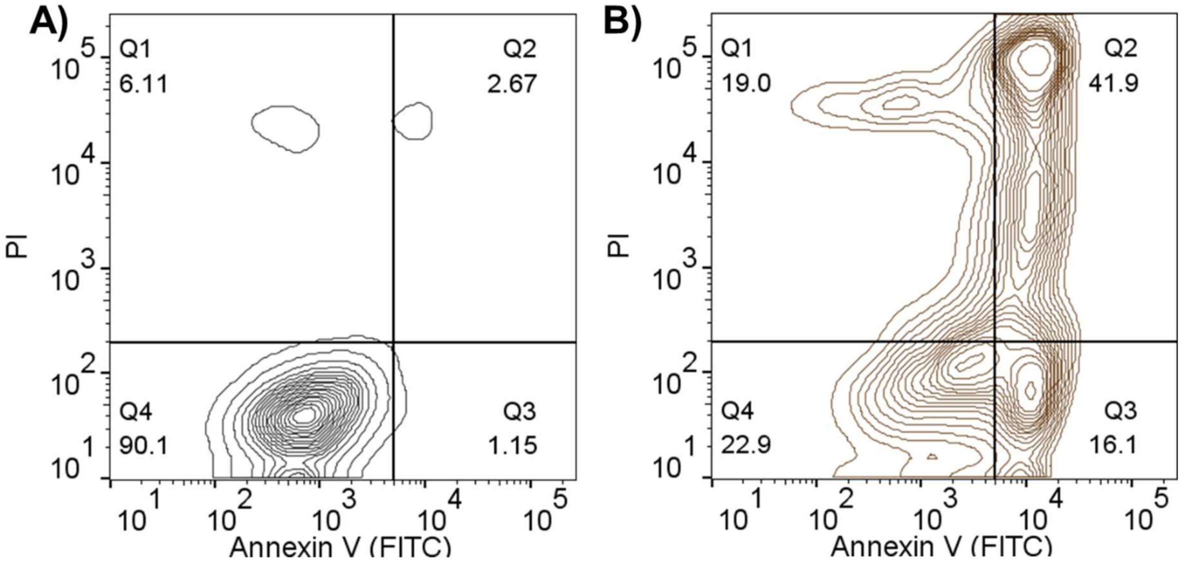

Fig. S45 Flow cytometry data for HUVEC cells stained with Annexin-V-FITC and PI. A) Untreated HUVECs and B) HUVECs treated with vcMMAE-LC40. Annexin-V-FITC, excitation: 488 nm, BP 527/32 and LP 507/5; PI, excitation: 640 nm, BP 660/10 [BP: Bandpass Filters and LP: Longpass Filters].

15. Flow Cytometry Analysis of Mixed Cell Populations

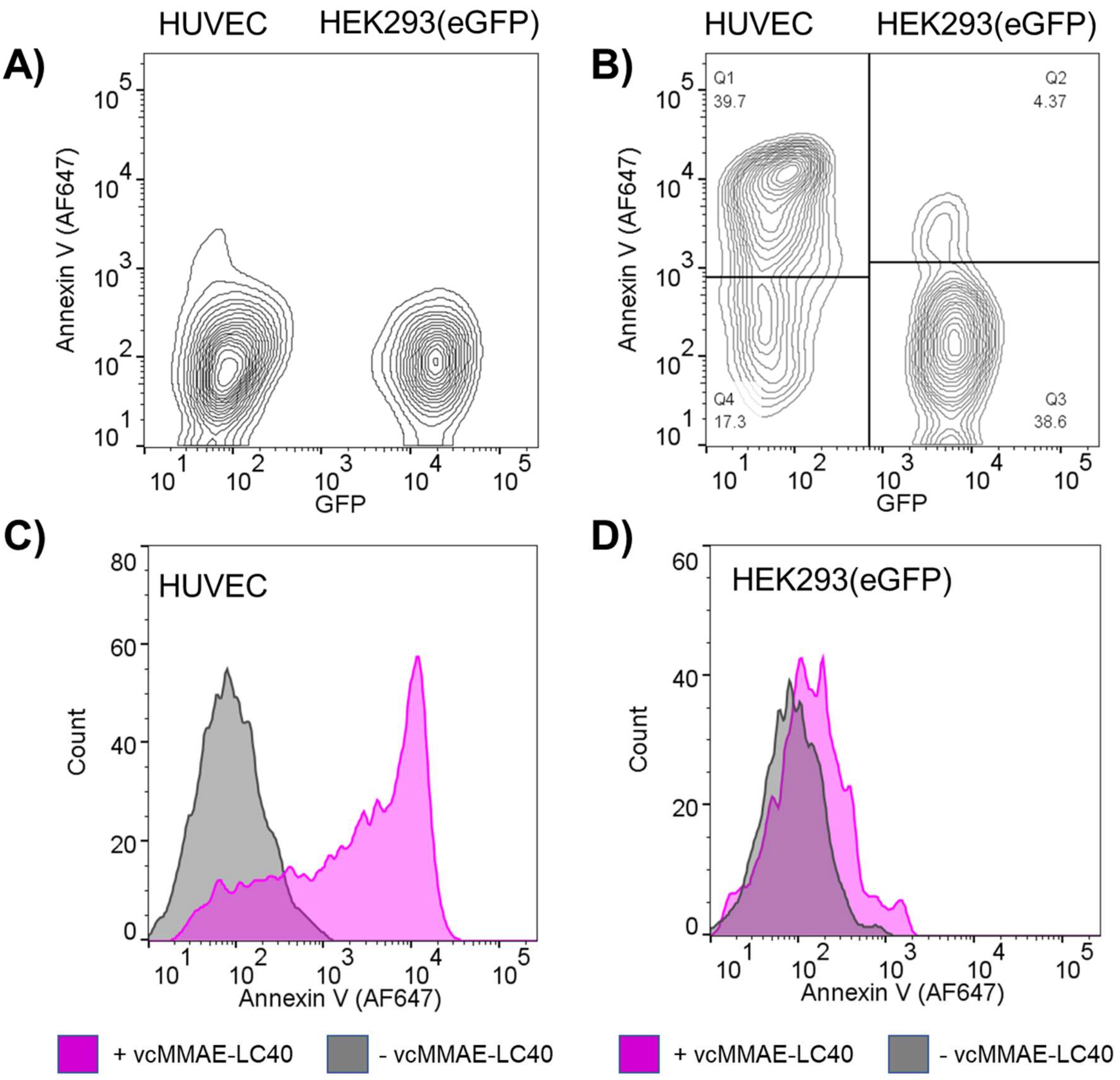

Fig. S46 Flow cytometry analysis of a mixture comprised of HUVECs and eGFP-MS2BP-expressing HEK-293 cells (HEK293(eGFP)) stained with AF647-labelled annexin V. Contour plots of A) untreated cells and B) cells treated with 200 nM vcMMAE-LC40. Histogram analysis of apoptosis assessed by annexin V staining of cell populations exhibiting C) low (HUVEC) and D) high (HEK293(eGFP)) GFP signals. GFP channel, excitation: 488 nm, BP 527/32 and LP 507/5; Annexin-V-AF647 channel, excitation: 640 nm, BP 660/10 [BP: Bandpass Filters and LP: Longpass Filters].

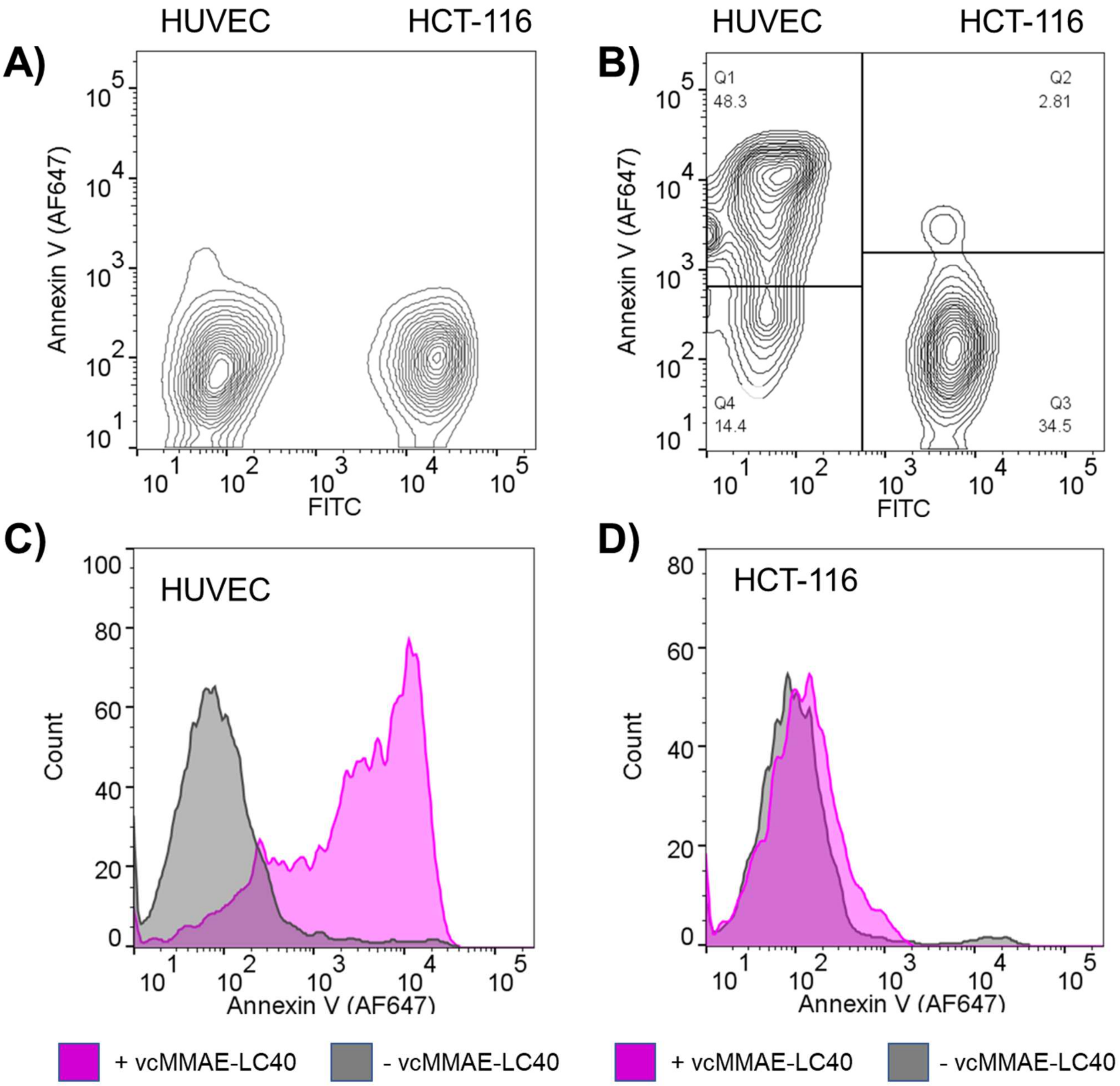

Fig. S47 Flow cytometry analysis of a mixture comprised of HUVECs and HCT-116 cells labelled with anti-EpCAM-FITC antibody stained with AF647-labelled annexin V. Contour plots of A) untreated cells and B) cells treated with 200 nM vcMMAE-LC40. Histogram analysis of apoptosis assessed by annexin V staining of cell populations exhibiting C) low (HUVEC) and D) high (HCT-116) FITC signals. FITC channel, excitation: 488 nm, BP 527/32 and LP 507/5; Annexin-V-AF647 channel, excitation: 640 nm, BP 660/10 [BP: Bandpass Filters and LP: Longpass Filters].

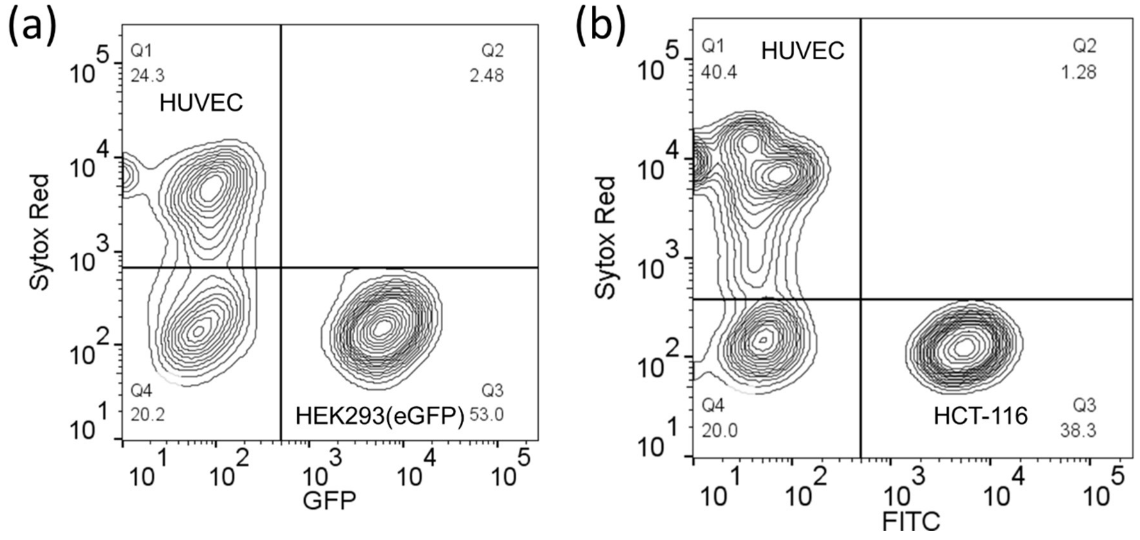

Fig. S48 Flow cytometry data for Sytox Red staining after vcMMAE-LC40 treatment (200 nM) of mixed cell populations including A) HUVECs and eGFP-MS2BP-expressing HEK-293 (HEK293(eGFP)) cells or B) HUVECs and anti-EpCAM-FITC antibody stained HCT-116 cells. The figures are enlarged versions of Fig. 6E and Fig. 6F, respectively.
